# Heterogeneous treatment effect estimation for observational data using model-based forests

**DOI:** 10.1177/09622802231224628

**Published:** 2024-02-08

**Authors:** Susanne Dandl, Andreas Bender, Torsten Hothorn

**Affiliations:** 1 Institut für Statistik, 9183 Ludwig-Maximilians-Universität München, Munich, Germany; 2 Munich Center for Machine Learning (MCML), Germany; 3 Institut für Epidemiologie, Biostatistik und Prävention, 27217 Universität Zürich, Zurich, Switzerland

**Keywords:** Heterogeneous treatment effects, personalized medicine, random forest, observational data, censored survival data, generalized linear model, transformation model

## Abstract

The estimation of heterogeneous treatment effects has attracted considerable interest in many disciplines, most prominently in medicine and economics. Contemporary research has so far primarily focused on continuous and binary responses where heterogeneous treatment effects are traditionally estimated by a linear model, which allows the estimation of constant or heterogeneous effects even under certain model misspecifications. More complex models for survival, count, or ordinal outcomes require stricter assumptions to reliably estimate the treatment effect. Most importantly, the noncollapsibility issue necessitates the joint estimation of treatment and prognostic effects. Model-based forests allow simultaneous estimation of covariate-dependent treatment and prognostic effects, but only for randomized trials. In this paper, we propose modifications to model-based forests to address the confounding issue in observational data. In particular, we evaluate an orthogonalization strategy originally proposed by Robinson (1988, Econometrica) in the context of model-based forests targeting heterogeneous treatment effect estimation in generalized linear models and transformation models. We found that this strategy reduces confounding effects in a simulated study with various outcome distributions. We demonstrate the practical aspects of heterogeneous treatment effect estimation for survival and ordinal outcomes by an assessment of the potentially heterogeneous effect of Riluzole on the progress of Amyotrophic Lateral Sclerosis.

## 1. Introduction

Over the past years, there has been emerging interest in methods to estimate heterogeneous treatment effects (HTEs) in various application fields. In healthcare, HTE estimation can be understood as a core principle driving personalized medicine. As opposed to average treatment effects, which assume a constant effect of a treatment on an outcome for the whole population, HTEs account for the heterogeneity in the effect for subgroups or individuals based on their characteristics. Most research on HTE estimation has mainly focused on continuous and binary response variables. These methods have typically built upon Rubin’s potential outcomes framework, a statistical approach to formulating and inferring causal effects in various designs.^1,2^

Traditionally, statistical models were used to estimate the treatment effect, but machine learning methods have been more and more adapted for these tasks over the past decade. Machine learning models rely on weaker assumptions and can automatically learn complex relationships such as higher order interaction effects, resulting in greater predictive performance in a variety of applications. In the case of continuous or binary responses, prominent methods to estimate HTEs are based on random forests,^3–8^ Bayesian additive regression trees (BART),^9,10^ or neural networks.^11–13^ Kuenzel et al.^14^ proposed general frameworks—T-learners, S-learners, U-learners, and X-learners—that base treatment effect estimates on arbitrary machine learning models. Chernozhukov et al.^15^ coined the term double/debiased machine learning models, which uses machine learning models for nuisance parameter estimation. The approach still relies on parametric models for estimating treatment effects, but Nie and Wager^16^ derived so-called R-learners that allow for arbitrary (nonparametric or semiparametric) models.

Beyond continuous or binary responses, research on machine learning methods for HTE estimation have primarily focused on (right-censored) survival data. Methods have been proposed based on BARTs,^17^ random forest-type methods,^18,19^ or deep learning approaches.^12,13^ Theoretically, any machine learning model for survival analysis—such as random survival forests^20^ or a Cox regression-based deep neural network (deepSurv)^21^—can estimate HTEs.^22^ These models can estimate survival or hazard functions in both treatment groups separately; HTEs are then defined as the difference in derived properties of the two functions, e.g. as differences in the median survival time. However, Hu et al.^22^ found that methods specifically designed for HTE estimation, like the adapted BART,^17^ produce more reliable estimates.

In general, for a continuous or binary outcome Y conditional on treatment w and covariates x , the conditional average treatment effect (CATE) τ(x) can be estimated from the model E(Y∣X=x,W=w)=μ(x)+τ(x)w even if the model is misspecified, e.g. when the prognostic effect μ(x) cannot be fully estimated due to missing covariate information. Beyond mean regression, stricter assumptions are necessary both for randomized and for observational studies to estimate HTEs. For example, under a true Cox model with survivor function exp(−exp(h(t)+μ(x)+τw)) with log-cumulative baseline hazard h(t) at time t and log-hazard ratio τ , the prognostic effect μ(x) must be specified correctly, even in a randomized trial. Estimated marginal log-hazard ratios $\hat{\tau }$ – i.e. when the model is fitted under the constraint μ(x)≡0 – are shrunken towards zero if this constraint is unrealistic.^23^ Naturally, this problem carries over to heterogeneous log-hazard ratios τ(x) .

Consequently, HTE estimation in more complex models requires the simultaneous estimation of both the prognostic part μ(x) and the predictive HTE τ(x) . Model-based forests allow simultaneous estimation of μ(x) and τ(x) ^24–29^ in the same forest model and have been demonstrated to perform at least on par with the best competitor in several independent studies evaluating HTE estimation in randomized trials.^30–33^ In a nutshell, model-based forests combine the parametric modeling framework with random forests to estimate individual treatment effects.^25^ By using generalized linear models and transformation models, model-based forests can be adapted for survival data,^24–26^ ordinal data,^27^ or clustered data.^28^

In observational studies, the treatment group assignment is not under control of the researcher and confounding effects could bias the estimation of HTEs. Robinson’s^34^ orthogonalization strategy has shown to be instrumental for achieving robustness to confounding effects for HTE estimation in observational studies.^5,35,36^ In this work, we propose and evaluate novel model-based forest variants based on the orthogonalization strategy that are suitable for HTE estimation in the observational setting. Compared to previous work, our work focuses on the utilization of model-based forests for HTE estimation and covers a richer class of models—(generalized) linear and transformation models. Simulation and application studies demonstrate the efficacy of our method for estimating HTEs under confounding biases yielding a versatile model framework applicable to a wide range of application cases. We contribute the first holistic approach to HTE estimation for observational studies which is applicable to many clinically relevant outcomes, including survival times, ordinal scores, binary events, or counts.

We review key components of model-based forests for HTE estimation in randomized trials in Section 2. In Section 3, we start introducing the orthogonalization approach by Robinson.^34^ We motivate previous developments using linear models^36^ and leverage adaptations to more complex models discussed by Gao and Hastie^35^ to define novel model-based forest variants suitable for HTE in the observational setting. These variants’ performances are empirically assessed in a simulation study with a range of outcome distributions in Section 4. Finally, in Section 5 presenting a re-analysis of the patient-specific effect of Riluzole in patients with amyotrophic lateral sclerosis (ALS), practical aspects of model estimation and interpretation are discussed.

## 2. Review of model-based forests for randomized trials

We are interested in estimating HTEs based on i.i.d. observations $\left(y_{i},x_{i},w_{i}\right)$ with i=1,…,n , where $y_{i}$ , $x_{i}$ and $w_{i}$ are realizations of the outcome Y , covariates X∈X , and control vs. treatment indicator W∈{0,1} . For the remainder of this manuscript (with the exception of equation (4)), we will omit the subscript i . Y(0) and Y(1) denote the potential outcomes under the two treatment conditions W∈{0,1} . Throughout this paper, we assume that X includes all relevant variables to explain heterogeneity both in the treatment effect and the outcome Y , and that the base model underlying model-based forests is correctly specified.

We review model-based forests for HTE estimation based on randomized trials as introduced by Seibold et al.^25^ and Korepanova et al.^26^ Within this section, we only consider settings where the treatment assignment is randomized and, therefore, follows a binomial model W∣X=x∼B(1,π(x)) with constant propensities π(x)≡π . We omit discussion of the abstract framework underlying model-based forests and instead discuss the important linear, generalized linear,^25^ and transformation models^26^ in detail.

### 2.1. Linear model

For a continuous outcome Y∈R with symmetric error distribution, a model-based forest might be defined based on the model

(1) (Y∣X=x,W=w)=μ(x)+τ(x)w+ϕZ

where the residuals are given by the error term ϕZ with E(Z|X,W)=0 and standard deviation ϕ>0 .^36^ We are mainly interested in estimating τ(x) , the treatment effect that depends on *predictive* variables in x . With model-based forests, however, we also obtain an estimated value for the prognostic effect μ(x) , which depends on *prognostic* variables in x . A variable might be predictive and prognostic at the same time. We refer to these situations as “overlays.”

Because we assume in this section that π(x)≡π applies, W⊥⊥X holds. Consequently, τ(x) can be interpreted as a CATE

(2) τ(x)=CATE(x)=E(Y(1)−Y(0)∣X=x)

on the absolute scale. To estimate $(\mu (x),\tau (x){)}^{⊤}$ the $L_{2}$ loss

(3) $$\ell (\mu (x),\tau (x))=\frac{1}{2}{\left(Y-\mu (x)-\tau (x)w\right)}^{2}$$

is minimized w.r.t. μ and τ using an ensemble of trees. Inspired by recursive partitioning techniques,^37,38^ split variable and split point selection are separated. Specifically in every node, μ and τ in the constant model E(Y∣W=w)=μ+τw are estimated using the observations in the node. The split variable is then the variable that has the lowest p -value for the bivariate permutation tests for the $H_{0}$ -hypothesis that μ and τ are constant and independent of any split variable. The cut-point is the point of the chosen split variable at which the score functions

$$s(\hat{\mu },\hat{\tau }):=(Y-\hat{\mu }-\hat{\tau }w)(1,w{)}^{⊤}$$

in the two resultant subgroups differ the most; details are available in Appendix 2 of Seibold et al.^25^

Once B∈N trees were fitted to subsamples of the training data, predictions for the treatment effect for a new observation x are obtained via local maximum likelihood aggregation.^5,29,39–41^ First, for the i th training sample, the frequency $\alpha_{i}(x)$ with which it falls in the same leaf as x over all B trees is measured. The obtained weighting vector $\left(\alpha_{1}(x),\ldots ,\alpha_{n}(x)\right)$ is used as an input for minimizing

(4) $$\left(\hat{\mu }(x),\hat{\tau }(x){)}^{⊤}=\underset{\mu ,\tau }{\arg\;\min}\;\sum_{i=1}^{n}\alpha_{i}(x)\ell_{i}(\mu ,\tau \right)$$

where $\ell_{i}$ denotes the loss for the i th sample. Model-based forests easily allow adaptions if HTEs for an outcome variable Y that is not well represented by equation (1) should be estimated. In this case, model-based forests can build on generalized linear models or transformation models in the recursive partitioning framework.^38^ As detailed in the following sections, the loss function ℓ in equation (3) changes from the squared error to the negative (partial) log-likelihood of some appropriate model.

### 2.2. Generalized linear models

When the conditional outcome distribution is better described through a generalized linear model

(Y∣X=x,W=w)∼ExpFam(θ(μ(x)+τ(x)w),ϕ)

with parameter θ depending on the additive function μ(x)+τ(x)w , the conditional mean

(5) $$g(E(Y|X=x,W=w))=\mu (x)+\tau (x)w=:\eta_{w}(x)$$

is linear on the scale of a link function g . Thus, the interpretation of τ(x) as CATE (2) generally no longer holds. Instead, the predictive effect is understood as the difference in natural parameters (DINA)^35^

(6) $$\tau (x)=\text{DINA}(x)=\eta_{1}(x)-\eta_{0}(x)$$

In contrast to the linear model case, HTEs τ(x) are now defined on relative scales, such as odds ratios in binary logistic regression models or multiplicative mean effects in a Poisson or Gaussian model with a log-link. The negative log-likelihood contribution of some observation (Y,x,w) is

ℓ(μ,τ,ϕ)=−log(f(Y∣θ(μ(x)+τ(x)w),ϕ))

with f as the conditional density of an exponential family distribution

f(Y∣θ(μ(x)+τ(x)w),ϕ)

Model-based trees and forests^38,24,25^ jointly estimate the prognostic effect μ(x) and the predictive effect τ(x) . The procedure simultaneously minimizes the negative log-likelihood with respect to μ(x) and τ(x) . In each node of the model-based forest, μ , τ , and potentially ϕ are estimated by minimizing

(7) ℓ(μ,τ,ϕ)=−log(f(Y∣θ(μ+τw),ϕ))

and regressing the bivariate gradient

$${\frac{\partial \ell (\mu ,\tau ,\phi )}{\partial (\mu ,\tau )}|}_{\hat{\mu },\hat{\tau },\hat{\phi }}$$

on x . This means that one is not explicitly looking for changes in the scale parameter ϕ , but this could be implemented by looking at the three-variate gradient

$${\frac{\partial \ell (\mu ,\tau ,\phi )}{\partial (\mu ,\tau ,\phi )}|}_{\hat{\mu },\hat{\tau },\hat{\phi }}$$

for example, in a heteroscedastic normal linear model

(Y∣X=x,W=w)=μ(x)+τ(x)w+ϕ(x)Z

After the tree fitting phase, an HTE is estimated with equation (4) with ℓ(μ,τ,ϕ) of equation (7) as the corresponding loss function.

Thus, model-based forests can be directly applied to estimate HTEs on relative scales for binary outcomes (binary logistic or probit regression, for example), counts (Poisson or quasi-Poisson regression), or continuous outcomes where a multiplicative effect is of interest (normal model with log-link).

### 2.3. Transformation models

More complex responses like ordered categorical or time-to-event outcomes are not covered by generalized linear models but can be analyzed using transformation models; corresponding model-based forests for survival analysis have been introduced by Korepanova et al.^26^ For some at least ordered outcome Y , we write the conditional distribution function as

(8) $$P(Y\leq y|X=x,W=w)=F(h(y)-\underset{=:\eta_{w}(x)}{\underbrace{\left(\mu (x)+\tau (x)w\right)}})$$

The transformation function h is monotone non-decreasing and the inverse link function F governs the interpretability of τ as log-odds ratios ( $F={\mathrm{logit}}^{-1}$ ), log-hazard ratios ( $F={\mathrm{cloglog}}^{-1}$ ), log-reverse time hazard ratios ( $F={\mathrm{loglog}}^{-1}$ ), or shift effects ( F=Φ , the cumulative distribution function of the standard normal). The shift term $\eta_{w}(x)$ differs between the two treatment groups w∈{0,1} . The distribution functions of the potential outcomes are F(h(y)−μ(x)) for Y(0) and F(h(y)−μ(x)−τ(x)) for Y(1) . The negative log-likelihood of a discrete or interval-censored observation ( $\underline{y},\bar{y}]$ (where $\underline{y}$ is the lower interval bound, $\bar{y}$ is the upper) is

$$\begin{array}{rl} \ell_{\text{Trafo}}(h,\mu ,\tau ) & =-\log\;(P(\underline{y}<Y\leq \bar{y}|X=x,W=w))\\ & =-\log\;(F(h(\bar{y})-\mu (x)-\tau (x)w)-F(h(\underline{y})-\mu (x)-\tau (x)w)) \end{array}$$

For a continuous datum y∈R , we obtain

$$\ell_{\text{Trafo}}(h,\mu ,\tau )=-\{\log\;(F^{\prime }(h(y)-\mu (x)-\tau (x)w))+\log\;(h^{\prime }(y))\};$$

details are given in Hothorn et al.^42^ Transformation forests apply the model-based recursive partitioning principle and estimate τ in each node along with the transformation function h (a “nuisance” parameter) by minimizing $\ell_{\text{Trafo}}(h,\mu \equiv 0,\tau )$ .^29^ Because h contains an intercept term, the parameter μ is not identified. We thus estimate the model under the constraint μ≡0 . Variable and cut-points are selected using the bivariate gradient

$${\frac{\partial \ell_{\text{Trafo}}(h,\mu \equiv 0,\tau )}{\partial (\mu ,\tau )}|}_{\mu =0,\hat{\tau }}$$

This model family includes proportional odds logistic regression (for ordered categorical, count or continuous outcomes), Box-Cox type models, Cox proportional hazards model, Weibull proportional hazards models for discrete and continuous outcomes, reverse time proportional hazards models relying on Lehmann alternatives, and many more.^42^ Forests for ordinal outcomes were evaluated by Buri and Hothorn,^27^ and a general approach to “transformation forests” is described in Hothorn and Zeileis.^29^

Application of the ideas underlying model-based forests allows HTEs to be estimated for such outcomes under all types of random censoring and truncation.^26^ For example, for Weibull distributed outcomes under right censoring, $h(y)=\nu_{1}+\nu_{2}\log\;(y)$ is chosen for the conditional distribution function in equation (8).^42^

In this case, we define Y as the event time, C as the censoring time and T=min(Y,C) as the observed time. For identification of τ(x) under potential censoring, the following assumption must hold^18^:

Assumption 1 **(Ignorable censoring)**

Censoring time C is independent of survival time Y conditional on treatment indicator W and covariates X

(Y(0),Y(1))⊥⊥C∣X=x,W=w

An important special case represents the Cox proportional hazards model, where the profile likelihood over the baseline hazard function defines the partial log-likelihood $\ell_{\text{PL}}(\mu ,\tau )$ with μ≡0 . The scores with respect to the constant μ≡0 are known as martingale residuals. Model-based forests for such models, and extensions to time-varying prognostic and predictive effects, are discussed in Korepanova et al.^26^

### 2.4. Noncollapsibility

As mentioned in the introduction, one problem with the Cox model is that misspecifications of prognostic effects μ(x) lead to biased estimates such that the estimated hazard ratios cannot be interpreted causally. This issue arises from the noncollapsiblity of the Cox model, the notion of which is characterized by the fact that in these models, the mean of the conditional effect estimates defined over covariates X does not coincide with the marginal effect over X . Because the noncollapsiblity of the Cox model arises from its nonadditivity of the hazard function, models such as a Weibull model (parameterized in form of an accelerated failure time model) do not suffer from this issue because they satisfy the additivity condition. Consequently, misspecifications of prognostic effects do not affect treatment effect estimates.^23^

The noncollapsibility issue is not limited to the Cox model but also affects members of the exponential family without identity or linear link functions. Without adjustments, effect estimates can only be interpreted causally if there is no treatment effect ( τ≡0 ) or there are no prognostic covariates.^43^

If this is not the case, specific methods are needed; ignoring the estimation of μ(x) at all and only focusing on τ(x) does not solve the problem. Conditioning on available prognostic variables is a common solution and is already applied by model-based forests because they estimate both the prognostic effect μ(x) and τ(x) . The ensemble of trees used to estimate these effects provides a high degree of flexibility and might therefore retain some of the potential complexity in the underlying μ(x) to mitigate misspecification. Whether conditioning resolves the noncollapsibility issue depends heavily on the assumption that all prognostic variables are known which is often not the case in the real world.^23^

For members of the exponential family and the Cox model, Gao and Hastie^35^ derived a method to account for noncollapsibility in the context of observational data with confounding effects. While we consider the noncollapsibility issue beyond the scope of this work, we briefly review the work of Gao and Hastie and discuss its applicability to model-based forests in Section A of the Supplemental Material.

## 3. Model-based forests for observational studies

In the previous section, we described model-based forests in the randomized setting under the assumption that π(x)≡π . In observational studies, in which the treatment group assignment is not under the control of the researcher, the propensity score (and therefore, the probability of being in the treatment group) often depends on covariates x

(9) π(x):=P(W=1∣X=x)=E(W∣X=x)

In this case, confounding effects could bias the estimation of treatment effects τ(x) , and stricter assumptions are necessary in order to interpret τ(x) causally.^44^

Assumption 2 **(Ignorability/Unconfoundedness)**

The treatment assignment is independent of the potential outcomes conditional on covariates x

(Y(0),Y(1))⊥⊥W∣X=x

Assumption 3 **(Positivity)**

The propensity score π(x) must be bounded away from 0 and 1

0<π(x)=P(W=1∣X=x)=E(W∣X=x)<1

Assumption 2 could be violated by an unmeasured confounder, while Assumption 3 could be violated if all observations in a certain group (defined via x ) are in the treatment group. Effects and potential solutions to these violations are well-studied in the literature.^45–48^

Dandl et al.^36^ showed for mean regression models that model-based forests are not robust to confounding effects and need further adaptions to estimate causal effects in case of observational data. One strategy for dealing with confounding effects is the orthogonalization strategy originally introduced by Robinson,^34^ which has received considerable attention in recent years.^15,5,16^ The reformulation of the linear model

(10) (Y∣X=x)=μ(x)+τ(x)W+ϕZ

to

(11) $$\begin{array}{r} (Y|X=x)=m(x)-m(x)+\mu (x)+\tau (x)W+\phi Z=m(x)+\tau (x)(W-\pi (x))+\phi Z \end{array}$$

given the conditional mean function

(12) m(x):=E(Y∣X=x)=μ(x)+τ(x)π(x)

motivates this approach.^5,36^

Overall, the orthogonalization strategy consists of two steps: First, nuisance parameters m(x)=E(Y∣X=x) and π(x)=P(W=1∣X=x) are estimated. Originally, Robinson^34^ used kernel estimators, but any machine learning method could be employed.^15,16^ Regressing $Y-\hat{m}(x)$ on $W-\hat{\pi }(x)$ then yields unbiased estimates for τ(x) . Subtracting $\hat{m}(x)$ and $\hat{\pi }(x)$ from Y and W , respectively, partially eliminates the association between X and Y and between X and W . The orthogonalization strategy has the distinct advantage over other methods against confounding—such as inverse propensity weighting and matching—that it is stable for extreme propensity scores and forgoes stratification.^35^

Robinson^34^ and Chernozhukov et al.^15^ use parametric models to estimate treatment effects based on residualized W and Y , but these models could be replaced by non-parametric or local parametric models^16,49^—such as model-based forests. For mean regression, Dandl et al.^36^ adapted the orthogonalization strategy to model-based forests. Their approach closely follows causal forests, which were the first to combine the orthogonalization strategy with tree-based estimators for τ(x) .

Gao and Hastie^35^ proposed extensions of Robinson’s strategy to members of the exponential family and the Cox model, where DINA (6) is of interest. Gao and Hastie^35^ assume $\tau (x)=x^{⊤}\beta$ and use parametric models to estimate τ(x) , but they conclude that non-parametric or local parametric models could be applied instead. We review model-based forests in combination with linear models for observational data in the next section and summarize the idea by Gao and Hastie^35^ in Section 3.2. On this basis, we assess how the orthogonalization strategy could be employed in model-based forests beyond mean regression with generalized linear models and transformation models as base models.

### 3.1. Review of Dandl et al.^36^

As noted above, Athey et al.^5^ were the first to combine the orthogonalization strategy of Robinson with tree-based estimators to estimate τ(x) . First, the marginal model m(x)=E(Y∣X=x) and propensity score π(x)=E(W∣X=x) are estimated by regression forests. Afterwards, causal forests estimate individual treatment effects τ(x) in the model

(13) $$(Y|X=x,W=w)=\hat{m}(x)+\tau (x)(w-\hat{\pi }(x))+\phi Z$$

using the “locally centered” outcomes $Y-\hat{m}(x)$ and treatment indicators $W-\hat{\pi }(x)$ .

Equation (13) shows that causal forests and model-based forests share common foundations for mean regression. The main difference is that the splitting scheme of model-based forests allows splitting according to heterogeneity in both treatment and prognostic effects, whereas causal forests only split with respect to heterogeneity in treatment effects (in equation (11), μ(x) cancels out).

Dandl et al.^36^ identified which elements of both approaches lead to improved performance in randomized trials and observational studies. They defined and evaluated the following blended versions of model-based forests and causal forests:

(1) *
$\text{mob}(\hat{W},\hat{Y})$ *, which uses model-based forests to estimate the model
$$E(Y|X=x,W=w)=\hat{m}(x)+\tilde{\mu }(x)+\tau (x)(w-\hat{\pi }(x))$$
  i.e. after centering the treatment indicator w and the outcome Y . Both parameters $\tilde{\mu }$ and τ are estimated simultaneously.
(2) *
$\text{mob}(\hat{W})$ *, which uses model-based forests to estimate the model
$$E(Y|X=x,W=w)=\mu (x)+\tau (x)(w-\hat{\pi }(x))$$
  i.e. after only centering the treatment indicator w but *not* outcome Y . Both μ and τ are estimated.
(3) *cfmob*, a method which uses model-based forests to estimate the model
$$E(Y|X=x,W=w)=\hat{m}(x)+\tau (x)(w-\hat{\pi }(x))$$
  i.e. after only centering the treatment indicator w and splitting only according to $\hat{\tau }$ . That is, only the parameters τ are estimated in this variant.

Their blended approaches competed with the original implementations of (uncentered) model-based forests and causal forests in an extensive simulation study. In case of confounding, the authors identified local centering of treatment indicator w and simultaneous estimation of both predictive *and* prognostic effects of the treatment indication (* $\text{mob}(\hat{W})$ *) as the key driver for good performance. Additionally, centering Y (* $\text{mob}(\hat{W},\hat{Y})$ *) is recommended, because it further improved performances in some cases. Splitting only according to $\hat{\tau }$ but not $\hat{\mu }$ (*cfmob*) resulted in lower predictive performance.

### 3.2. Review of Gao and Hastie (2022)

Robinson^34^ derived the orthogonalization strategy only for semi-parametric additive models with Y∈R . Gao and Hastie^35^ extended the idea to a broader class of distributions including the exponential family and Cox model.

Local centering of the treatment indicator works analogously to mean regression. First, propensity scores π(x)=P(W∣X=x) are estimated. The effects of the covariates X on the treatment assignment are then regressed out by subtracting $\hat{\pi }(x)$ from W .

Orthogonalization of Y is not straightforward due to the link function that relates the linear predictor $\eta_{w}(x)$ in equation (5) to the outcome Y . To understand how Gao and Hastie derived m(x) to center Y , we consider equation (10) as a model of the exponential family with identity link function g . Now we can rewrite equation (12) to

$$\begin{array}{rl} g(E(Y|X=x)) & =E_{W}(g(E(Y|X=x,W=w)))\\ & =\pi (x)\underset{=\eta_{1}(x)}{\underbrace{\left(\mu (x)+\tau (x)\right)}}+(1-\pi (x))\underset{=\eta_{0}(x)}{\underbrace{\mu (x)}}\\ & =\mu (x)+\pi (x)\tau (x)=m(x) \end{array}$$

Accordingly, Gao and Hastie derived g(E(Y∣X=x)) for all other distributions of the exponential family by

(14) $$m(x)=\pi (x)\eta_{1}(x)+(1-\pi (x))\eta_{0}(x)$$

We can regard the estimated m(x) as an offset in the linear predictor

$$\hat{m}(x)+\tau (x)(W-\hat{\pi }(x))$$

Note that equation (14) states that (only) for the Gaussian distribution we can directly estimate m(x)=E(Y∣X=x) without estimating $\eta_{0}(x)$ and $\eta_{1}(x)$ . We can also derive $\hat{m}(x)$ for transformation models based on the definition of $\eta_{0}$ and $\eta_{1}$ in equation (8). As mentioned in Section 2.4, compared to the difference in conditional means, the DINA additionally suffers from the noncollapsibility issue.^50^ Gao and Hastie^35^ also extend the Robinson strategy to tackle not only the confounding but also the noncollapsibility issue for members of the exponential family (without a linear or log-link function, otherwise confounding is not an issue) and the Cox model. While it is beyond the scope of this work to address the noncollapsibility issue, Section A of the Supplemental Material briefly summarizes and discusses the applicability of Gao and Hastie’s approach to model-based forests.

### 3.3. Novel model-based forests for observational data

As stated above, our main goal is to assess how the orthogonalization strategy proposed for continuous outcomes could be extended to models beyond mean regression, specifically generalized linear models and transformation models. Based on Dandl et al.^36^ and Gao and Hastie^35^ we propose two different versions of model-based forests, which should be more robust against confounding. Following Dandl et al.,^36^ we formulate research questions for these versions, which we aim to answer empirically in Section 4. An overview of all proposed versions is given in Table 1.

**Table 1.** Overview of proposed model-based forest versions.

Method	Linear predictor	Definitions
*Naive*	μ(x)+τ(x)w
${\mathrm{Robinson}}_{\hat{W}}$	$\mu (x)+\tau (x)(w-\hat{\pi }(x))$	π(x)=P(W=1|X=x)
*Robinson*	$\hat{m}(x)+\tilde{\mu }(x)+\tau (x)(w-\hat{\pi }(x))$	$m(x)=\pi (x)\eta_{1}(x)-(1-\pi (x))\eta_{0}(x)$

The first version of model-based forests directly applies Robinson’s orthogonalization strategy: First, we estimate propensities π(x) as well as $\eta_{0}(x)$ and $\eta_{1}(x)$ to derive $\hat{m}(x)$ . Then, we update the linear predictor of equation (5) by centering W by $\hat{\pi }(x)$ and by adding the offset $\hat{m}(x)$ . For generalized linear models, we obtain

$$g(E(Y|X=x,W=w))=\hat{m}(x)+\tilde{\mu }(x)+\tau (x)(w-\hat{\pi }(x))$$

and for the conditional distribution function of equation (8) in case of transformation models

$$F[h(y)-\{\hat{m}(x)+\tilde{\mu }(x)+\tau (x)(w-\hat{\pi }(x)\}]$$

Based on the updated models, both prognostic and predictive effects $\tilde{\mu }(x)$ and τ(x) are simultaneously estimated by model-based forests.

In the simulation study and practical example in Sections 4 and 5, we use regression forests to estimate π(x) and gradient boosting machines (with tailored loss functions) to estimate $\eta_{0}$ and $\eta_{1}$ . In the following, we denote this version of model-based forests as *Robinson* forests in recognition of Robinson^34^ while model-based forests without centering W and without offset $\hat{m}(x)$ are called *Naive* forests.

Similar to Dandl et al.–who saw an improvement in performance when only centering W (compared to the naive model-based forests)—we define an approach called ${\mathrm{Robinson}}_{\hat{W}}$ forests that applies model-based forests to models with linear predictors

$$\mu (x)+\tau (x)(w-\hat{\pi }(x))$$

## 4. Empirical evaluation

In a simulation study, we aim to compare the different model-based forest versions (Table 1) by answering the following research questions:

RQ 1 To what extent does centering W by $\hat{\pi }(x)$ and including $\hat{m}(x)$ as an offset (*Robinson*) affect the performance of model-based forests in the presence of confounding?
RQ 2 Do centered treatment indicator model-based forests ( ${\mathrm{Robinson}}_{\hat{W}}$ ) perform better than uncentered model-based forests in the presence of confounding?
RQ 3 Are model-based forests with centered treatment indicators ( ${\mathrm{Robinson}}_{\hat{W}}$ ) relevantly outperformed by model-based forests with $\hat{m}(x)$ as an additional offset (*Robinson*) in the presence of confounding?

The study includes different outcome types, different predictive and prognostic effects, and a varying number of observations and covariates. Model-based forests were fitted with the model4you R add-on package.^51^ Similar to Dandl et al.,^36^ we base our study settings on the four setups (A, B, C and D) of Nie and Wager.^16^ In addition, in Section B of the Supplemental Material, we show the results for simulation settings first proposed by Wager and Athey^49^ and later reused by Athey et al.^5^

### 4.1. Data generating process

Given P={10,20} , for Setup A, we sampled $X∼U([0,1{]}^{P})$ . For all other setups, we used $X∼N(0,1_{P\times P})$ . The treatment indicator was binomially distributed with W∣X=x∼B(1,π(x)) . The propensity function π(x) differed for the four considered setups:

$$\begin{array}{r} \pi (x)=\left\{ \begin{array}{l} \pi_{A}(x_{1},x_{2})=\max\{0.1,\min\{\sin\;(\pi x_{1}x_{2}),1-0.1\}\}\\ \pi_{B}\equiv 0.5\\ \pi_{C}(x_{2},x_{3})=1/(1+\exp\;(x_{2}+x_{3}))\\ \pi_{D}(x_{1},x_{2})=1/(1+\exp\;(-x_{1})+\exp\;(-x_{2})) \end{array}\right. \end{array}$$

π(x)≡0.5 in Setup B implies a randomized study. The treatment effect function τ(⋅) and the prognostic effect function μ(⋅) also differed between the setups

$$\begin{array}{r} \tau (x)=\left\{ \begin{array}{l} \tau_{A}(x_{1},x_{2})=(x_{1}+x_{2})/2\\ \tau_{B}(x_{1},x_{2})=x_{1}+\log\;(1+\exp\;(x_{2}))\\ \tau_{C}\equiv 1\\ \tau_{D}(x_{1},x_{2},x_{3},x_{4},x_{5})=\max\{x_{1}+x_{2}+x_{3},0\}-\max\{x_{4}+x_{5},0\} \end{array}\right. \end{array}$$

$$\begin{array}{r} \mu (x)=\left\{ \begin{array}{l} \mu_{A}(x_{1},x_{2},x_{3},x_{4},x_{5})=\sin\;(\pi x_{1}x_{2})+2(x_{3}-0.5{)}^{2}+x_{4}+0.5x_{5}\\ \mu_{B}(x_{1},x_{2},x_{3})=\max\{x_{1}+x_{2},x_{3},0\}+\max\{x_{4}+x_{5},0\}\\ \mu_{C}(x_{1},x_{2},x_{3})=2\log\;(1+\exp\;(x_{1}+x_{2}+x_{3}))\\ \mu_{D}(x_{1},x_{2},x_{3},x_{4},x_{5})=(\max\{x_{1}+x_{2}+x_{3},0\}+\max\{x_{4}+x_{5},0\})/2 \end{array}\right. \end{array}$$

Setup A has extensive confounding that must be eliminated before estimating an easily predictable treatment effect function τ(x) . Setup B needs no confounding adjustment for reliable estimation of τ . Although Setup C contains strong confounding, the propensity score function is easier to estimate than the prognostic effect, while the treatment effect is constant. In Setup D, the treatment and control arms are unrelated, and therefore, learning the conditional expected outcomes of both arms jointly is not beneficial.^16,36^

To assess robustness of the methods to missing prognostic covariates, we additionally created Setup A’ from Setup A by removing covariate $X_{3}$ from the training data. Therefore, the DGP of Setup A and Setup A’ are identical, the only difference being that the training data did not contain $X_{3}$ although $X_{3}$ affects the prognostic effect.

We studied four different simulation models

$$(Y|X=x,W=w)∼\left\{ \begin{array}{rl} & N\;(\mu (x)+\tau (x)(w-0.5),1)\;\left(15a\right)\\ & B\;(1,\text{expit}(\mu (x)+\tau (x)(w-0.5)))\;\left(15b\right)\\ & M\;\text{with}\log\;(O(y_{k}|x,w))=\vartheta_{k}-\mu (x)-\tau (x)(w-0.5)\;\left(15c\right)\\ & W\;\text{with}\log\;(H(y|x,w))=2\log\;(y)-\mu (x)-\tau (x)(w-0.5)\;\left(15d\right) \end{array}\right.$$

Model (15a) is a normal linear regression model, model (15b) is a binary logistic regression model, model (15c) is a 4-nomial model with log-odds function $\vartheta_{k}-\mu (x)-\tau (x)(w-0.5)$ with threshold parameters $\vartheta_{k}=\text{logit}(k/4)$ for k=1,2,3 , and model (15d) is a Weibull model with log-cumulative hazard function 2log(y)−μ(x)−τ(x)(w−0.5) . We added 50% random right-censoring to the Weibull-generated data. Additionally, we applied a Cox proportional hazards model to the Weibull data to determine if the performance of model-based forests degrades when the forests do not take the true underlying model as their base model.

Due to w−0.5 in all scenarios, half of the (negative) predictive effect τ(x) was added to the prognostic effect. We refer to the implied scenario—where one variable which is both prognostic (impact in μ(x) ) and predictive (impact in τ(x) ) exists—as overlay. Apart from Setup C in which the treatment effect is constant and independent of any covariate, overlay was present for all scenarios.

Like Dandl et al.,^36^ we compared all study settings and outcome types for a varying number of samples N∈{800,1600} and dimensions P∈{10,20} . All model-based forests were grown with the same hyperparameter options specified in Section 7. We used random forests as implemented in the grf package to estimate π(x) for centering W .^52^ To estimate $\eta_{0}(x)$ and $\eta_{1}(x)$ to derive $\hat{m}(x)$ , we relied on different tree-based estimators depending on the outcome type. For normally distributed outcomes (models (15a)), we used grf regression forests.^52^ For all other outcomes, we relied on gradient boosting machines (with adapted loss functions) as implemented in mboost and gbm.^53,54^ The employed distribution varied depending on the outcome type.

**Figure 1.**
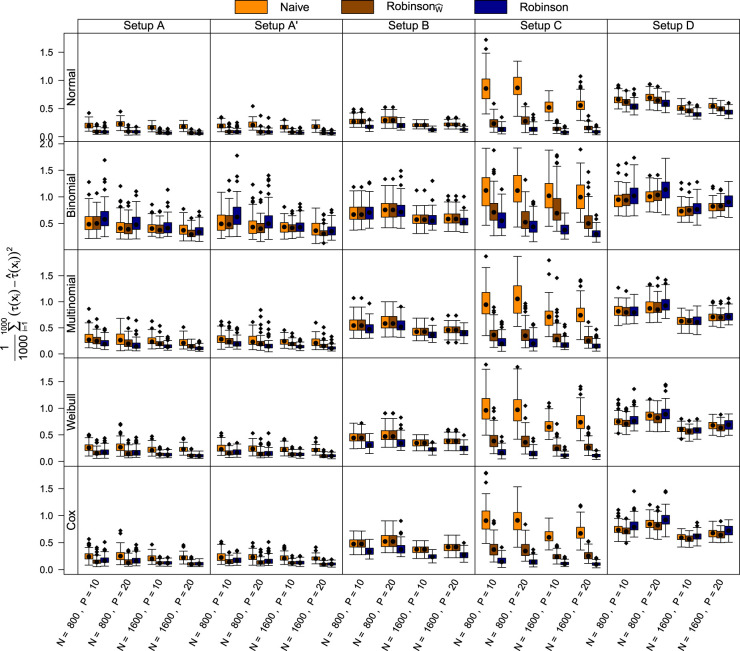
Model-based forest results for the empirical study (Section 4), Cox means a Cox model applied to the Weibull data. For the Weibull and Cox model, treatment effects τ(x) are estimated as conditional log hazard ratios. Direct comparison of model-based forests without centering (*Naive*), model-based forests with local centering according to Robinson^34^ of Y and W (originally proposed) (*Robinson*) or only of W ( ${\mathrm{Robinson}}_{\hat{W}}$ ).

In accordance with Dandl et al.,^36^ we evaluated the models with respect to the mean squared error $E_{X}\{(\hat{\tau }(X)-\tau (X){)}^{2}\}$ , bias $E_{X}\{\hat{\tau }(X)-\tau (X)\}$ and standard error $\sqrt{Var_{X}\{\hat{\tau }(X)-\tau (X)}\}$ on a test sample of size 1000 . The results for the mean squared error are shown in Figure 1 and were statistically analyzed by means of a normal linear mixed model with a log-link. The model explained the estimated mean squared error for $\hat{\tau }(x)$ by a four-way interaction of the data generating process, sample size N , dimension P , and random forest variant. We estimated the mean squared error ratios between different model-based forest versions according to the research questions stated in Section 4. The corresponding tables are given in Tables 2 to 4. In the Supplemental Material, the results for the bias and standard error are presented in Figures S. 2 and S. 3, as well as the statistical analysis of the relative efficiency in Tables S. 4 to S. 6.

**Table 2.** Results of **RQ 1** for the experimental setups in Section 4.

			Mean squared error ratio for **RQ 1**: Robinson vs. Naive				
DGP	N	P	Normal	Binomial	Multinomial	Weibull	Cox
Setup A	800	10	*0.465 (0.423, 0.511)*	**1.173 (1.051, 1.309)**	*0.690 (0.630, 0.756)*	*0.672 (0.610, 0.740)*	*0.712 (0.651, 0.780)*
		20	*0.396 (0.360, 0.437)*	**1.161 (1.020, 1.321)**	*0.600 (0.541, 0.665)*	*0.605 (0.548, 0.668)*	*0.654 (0.597, 0.717)*
	1600	10	*0.414 (0.363, 0.471)*	1.043 (0.900, 1.209)	*0.582 (0.513, 0.659)*	*0.580 (0.509, 0.661)*	*0.589 (0.519, 0.668)*
		20	*0.341 (0.296, 0.392)*	0.898 (0.757, 1.065)	*0.502 (0.429, 0.588)*	*0.471 (0.406, 0.546)*	*0.495 (0.430, 0.568)*
Setup A’	800	10	*0.481 (0.436, 0.531)*	**1.234 (1.112, 1.369)**	*0.675 (0.615, 0.741)*	*0.703 (0.634, 0.778)*	*0.730 (0.663, 0.804)*
		20	*0.418 (0.379, 0.460)*	**1.208 (1.069, 1.366)**	*0.647 (0.579, 0.723)*	*0.671 (0.602, 0.748)*	*0.714 (0.645, 0.791)*
	1600	10	*0.423 (0.373, 0.479)*	1.011 (0.875, 1.168)	*0.593 (0.520, 0.677)*	*0.595 (0.525, 0.674)*	*0.607 (0.538, 0.685)*
		20	*0.358 (0.312, 0.410)*	0.928 (0.785, 1.098)	*0.521 (0.449, 0.606)*	*0.497 (0.426, 0.579)*	*0.515 (0.445, 0.595)*
Setup B	800	10	*0.643 (0.609, 0.680)*	1.020 (0.933, 1.116)	*0.868 (0.831, 0.906)*	*0.692 (0.654, 0.732)*	*0.703 (0.669, 0.739)*
		20	*0.658 (0.626, 0.691)*	0.976 (0.897, 1.062)	*0.906 (0.870, 0.942)*	*0.716 (0.681, 0.753)*	*0.731 (0.700, 0.763)*
	1600	10	*0.603 (0.558, 0.651)*	0.981 (0.878, 1.095)	*0.852 (0.804, 0.902)*	*0.657 (0.608, 0.709)*	*0.656 (0.613, 0.702)*
		20	*0.588 (0.546, 0.634)*	0.912 (0.815, 1.020)	*0.869 (0.823, 0.916)*	*0.648 (0.604, 0.696)*	*0.653 (0.614, 0.694)*
Setup C	800	10	*0.153 (0.144, 0.163)*	*0.473 (0.434, 0.517)*	*0.250 (0.233, 0.267)*	*0.174 (0.160, 0.189)*	*0.176 (0.162, 0.191)*
		20	*0.156 (0.147, 0.166)*	*0.358 (0.323, 0.397)*	*0.219 (0.204, 0.234)*	*0.157 (0.143, 0.172)*	*0.161 (0.146, 0.176)*
	1600	10	*0.154 (0.139, 0.171)*	*0.360 (0.317, 0.409)*	*0.260 (0.239, 0.284)*	*0.181 (0.160, 0.205)*	*0.187 (0.166, 0.211)*
		20	*0.157 (0.143, 0.172)*	*0.299 (0.257, 0.348)*	*0.216 (0.195, 0.238)*	*0.147 (0.129, 0.168)*	*0.152 (0.133, 0.173)*
Setup D	800	10	*0.818 (0.802, 0.834)*	**1.110 (1.040, 1.183)**	0.996 (0.969, 1.025)	**1.036 (1.008, 1.064)**	**1.085 (1.058, 1.112)**
		20	*0.851 (0.835, 0.867)*	**1.126 (1.061, 1.196)**	**1.054 (1.028, 1.081)**	**1.055 (1.030, 1.080)**	**1.099 (1.075, 1.124)**
	1600	10	*0.783 (0.762, 0.804)*	1.075 (0.989, 1.170)	0.994 (0.958, 1.031)	0.968 (0.934, 1.003)	1.029 (0.996, 1.063)
		20	*0.803 (0.784, 0.823)*	**1.131 (1.050, 1.218)**	1.016 (0.984, 1.050)	1.021 (0.990, 1.052)	**1.076 (1.046, 1.107)**

Comparison of mean squared errors for $\hat{\tau }(x)$ in the different scenarios. Estimates and simultaneous 95 % confidence intervals were obtained from a normal linear mixed model with log-link. Cells printed in bold font correspond to a superior reference of the *Naive* model-based forests, and cells printed in italics indicate an inferior reference.

**Table 3.** Results of **RQ 2** for the experimental setups in Section 4.

			Mean squared error ratio for **RQ 2**: ${\mathrm{Robinson}}_{\hat{W}}$ vs. Naive				
DGP	N	P	Normal	Binomial	Multinomial	Weibull	Cox
Setup A	800	10	1.029 (0.913, 1.159)	*0.819 (0.732, 0.916)*	**1.259 (1.145, 1.385)**	0.924 (0.822, 1.040)	*0.844 (0.753, 0.946)*
		20	1.060 (0.937, 1.199)	*0.784 (0.684, 0.899)*	**1.282 (1.147, 1.433)**	0.935 (0.827, 1.057)	*0.835 (0.742, 0.941)*
	1600	10	1.126 (0.959, 1.323)	0.914 (0.786, 1.062)	**1.370 (1.198, 1.566)**	1.067 (0.914, 1.246)	1.015 (0.872, 1.182)
		20	1.164 (0.977, 1.386)	0.887 (0.733, 1.073)	**1.301 (1.090, 1.553)**	1.064 (0.885, 1.278)	0.994 (0.833, 1.186)
Setup A’	800	10	1.036 (0.917, 1.170)	*0.800 (0.721, 0.889)*	**1.267 (1.149, 1.397)**	0.911 (0.805, 1.030)	*0.857 (0.760, 0.965)*
		20	1.062 (0.941, 1.199)	*0.766 (0.673, 0.870)*	**1.323 (1.178, 1.486)**	0.915 (0.802, 1.044)	*0.847 (0.746, 0.962)*
	1600	10	1.140 (0.979, 1.327)	0.930 (0.801, 1.079)	**1.351 (1.173, 1.555)**	1.026 (0.883, 1.191)	0.972 (0.838, 1.126)
		20	1.109 (0.934, 1.318)	0.870 (0.722, 1.049)	**1.350 (1.144, 1.593)**	1.039 (0.859, 1.257)	0.971 (0.807, 1.169)
Setup B	800	10	**1.555 (1.471, 1.644)**	0.980 (0.896, 1.072)	**1.152 (1.103, 1.204)**	**1.445 (1.365, 1.529)**	**1.423 (1.354, 1.495)**
		20	**1.520 (1.447, 1.598)**	1.025 (0.942, 1.115)	**1.104 (1.061, 1.149)**	**1.396 (1.329, 1.468)**	**1.368 (1.310, 1.429)**
	1600	10	**1.658 (1.534, 1.792)**	1.019 (0.912, 1.138)	**1.174 (1.109, 1.244)**	**1.524 (1.411, 1.646)**	**1.525 (1.425, 1.633)**
		20	**1.700 (1.578, 1.832)**	1.097 (0.981, 1.227)	**1.151 (1.092, 1.215)**	**1.542 (1.437, 1.655)**	**1.532 (1.441, 1.628)**
Setup C	800	10	**1.871 (1.747, 2.005)**	**1.378 (1.250, 1.520)**	**1.577 (1.459, 1.705)**	**2.332 (2.133, 2.549)**	**2.389 (2.185, 2.611)**
		20	**2.081 (1.949, 2.222)**	**1.296 (1.147, 1.464)**	**1.718 (1.591, 1.856)**	**2.566 (2.324, 2.833)**	**2.612 (2.366, 2.883)**
	1600	10	**1.774 (1.579, 1.993)**	**2.629 (2.310, 2.992)**	**1.759 (1.597, 1.937)**	**2.199 (1.925, 2.511)**	**2.142 (1.878, 2.443)**
		20	**1.817 (1.635, 2.019)**	**1.802 (1.526, 2.128)**	**1.674 (1.498, 1.872)**	**2.542 (2.210, 2.923)**	**2.566 (2.232, 2.951)**
Setup D	800	10	**1.136 (1.113, 1.160)**	*0.910 (0.854, 0.970)*	0.992 (0.965, 1.021)	*0.916 (0.891, 0.942)*	*0.883 (0.860, 0.906)*
		20	**1.098 (1.077, 1.119)**	*0.898 (0.847, 0.953)*	*0.942 (0.919, 0.966)*	*0.909 (0.887, 0.931)*	*0.881 (0.861, 0.901)*
	1600	10	**1.147 (1.115, 1.179)**	0.950 (0.874, 1.033)	0.994 (0.958, 1.031)	0.965 (0.930, 1.000)	*0.923 (0.892, 0.954)*
		20	**1.126 (1.098, 1.156)**	*0.890 (0.826, 0.958)*	0.972 (0.940, 1.004)	*0.922 (0.893, 0.952)*	*0.888 (0.863, 0.914)*

Comparison of mean squared errors for $\hat{\tau }(x)$ in the different scenarios.Estimates and simultaneous 95 % confidence intervals were obtained from a normal linear mixed model with log-link. Cells printed in bold font correspond to a superior reference of the *Naive* model-based forests, and cells printed in italics indicate an inferior reference.

**Table 4.** Results of **RQ 3** for the experimental setups in Section 4.

			Mean squared error ratio for **RQ 3**: Robinson vs. ${\mathrm{Robinson}}_{\hat{W}}$				
DGP	N	P	Normal	Binomial	Multinomial	Weibull	Cox
Setup A	800	10	0.972 (0.863, 1.095)	**1.221 (1.092, 1.366)**	*0.794 (0.722, 0.874)*	1.082 (0.962, 1.217)	**1.185 (1.057, 1.327)**
		20	0.944 (0.834, 1.068)	**1.275 (1.113, 1.462)**	*0.780 (0.698, 0.872)*	1.069 (0.946, 1.209)	**1.197 (1.062, 1.349)**
	1600	10	0.888 (0.756, 1.043)	1.094 (0.941, 1.273)	*0.730 (0.639, 0.835)*	0.937 (0.803, 1.094)	0.985 (0.846, 1.147)
		20	0.859 (0.721, 1.024)	1.128 (0.932, 1.365)	*0.768 (0.644, 0.917)*	0.940 (0.782, 1.130)	1.006 (0.843, 1.200)
Setup A’	800	10	0.965 (0.854, 1.090)	**1.249 (1.125, 1.387)**	*0.789 (0.716, 0.871)*	1.098 (0.971, 1.242)	**1.167 (1.036, 1.315)**
		20	0.942 (0.834, 1.063)	**1.306 (1.149, 1.485)**	*0.756 (0.673, 0.849)*	1.093 (0.957, 1.248)	**1.181 (1.040, 1.341)**
	1600	10	0.877 (0.754, 1.022)	1.075 (0.926, 1.248)	*0.740 (0.643, 0.852)*	0.975 (0.839, 1.132)	1.029 (0.888, 1.193)
		20	0.902 (0.759, 1.071)	1.150 (0.954, 1.386)	*0.741 (0.628, 0.874)*	0.962 (0.796, 1.164)	1.029 (0.855, 1.239)
Setup B	800	10	*0.643 (0.608, 0.680)*	1.020 (0.933, 1.116)	*0.868 (0.831, 0.906)*	*0.692 (0.654, 0.733)*	*0.703 (0.669, 0.739)*
		20	*0.658 (0.626, 0.691)*	0.976 (0.897, 1.062)	*0.905 (0.870, 0.942)*	*0.716 (0.681, 0.753)*	*0.731 (0.700, 0.763)*
	1600	10	*0.603 (0.558, 0.652)*	0.981 (0.879, 1.096)	*0.852 (0.804, 0.902)*	*0.656 (0.608, 0.709)*	*0.656 (0.613, 0.702)*
		20	*0.588 (0.546, 0.634)*	0.912 (0.815, 1.020)	*0.868 (0.823, 0.916)*	*0.648 (0.604, 0.696)*	*0.653 (0.614, 0.694)*
Setup C	800	10	*0.534 (0.499, 0.572)*	*0.726 (0.658, 0.800)*	*0.634 (0.587, 0.686)*	*0.429 (0.392, 0.469)*	*0.419 (0.383, 0.458)*
		20	*0.481 (0.450, 0.513)*	*0.772 (0.683, 0.872)*	*0.582 (0.539, 0.629)*	*0.390 (0.353, 0.430)*	*0.383 (0.347, 0.423)*
	1600	10	*0.564 (0.502, 0.633)*	*0.380 (0.334, 0.433)*	*0.569 (0.516, 0.626)*	*0.455 (0.398, 0.519)*	*0.467 (0.409, 0.533)*
		20	*0.550 (0.495, 0.611)*	*0.555 (0.470, 0.655)*	*0.597 (0.534, 0.668)*	*0.393 (0.342, 0.453)*	*0.390 (0.339, 0.448)*
Setup D	800	10	*0.880 (0.862, 0.898)*	**1.099 (1.031, 1.172)**	1.008 (0.980, 1.037)	**1.092 (1.062, 1.123)**	**1.133 (1.104, 1.162)**
		20	*0.911 (0.894, 0.928)*	**1.113 (1.049, 1.181)**	**1.062 (1.035, 1.089)**	**1.101 (1.074, 1.128)**	**1.136 (1.110, 1.162)**
	1600	10	*0.872 (0.848, 0.897)*	1.052 (0.968, 1.144)	1.006 (0.970, 1.043)	1.037 (1.000, 1.075)	**1.084 (1.048, 1.121)**
		20	*0.888 (0.865, 0.911)*	**1.124 (1.044, 1.210)**	1.029 (0.996, 1.063)	**1.085 (1.051, 1.120)**	**1.126 (1.094, 1.159)**

Comparison of mean squared errors for $\hat{\tau }(x)$ in the different scenarios. Estimates and simultaneous 95 % confidence intervals were obtained from a normal linear mixed model with log-link. Cells printed in bold font correspond to a superior reference of ${\mathrm{Robinson}}_{\hat{W}}$ , and cells printed in italics indicate an inferior reference.

### 4.2. Results

The results for the normal distribution coincide with the results obtained by Dandl et al.^36^ summarized in Section 3.1. To some degree, they also hold for the other distributions. The boxplots are not directly comparable between different data generating processes because of different signal-to-noise ratios. In general, a more informative outcome (binary < ordered < right-censored < exact normal), more data (higher N ), and less noise (lower P ) leads to better results. Using a Cox model compared to a Weibull model (last two rows of Figure 1) did not lead to a major decrease in performance, although knowledge of the true functional form of the transformation function did not enter the Cox modeling process.

For Setup A, model-based forests without centering (*Naive*) were unable to cope with complex confounding, but solely centering of the treatment indicator ( ${\mathrm{Robinson}}_{\hat{W}}$ ) was valuable. Additionally adding $\hat{m}(x)$ as an offset (*Robinson*) did not further improve the results for the normal, binomial, and Weibull distributions, but an improvement was observed for the multinomial distribution. Suppression of $X_{3}$ in the training dataset (Setup A’) had little to no effect on the ranking of the methods. Slight deterioration in the performance of all methods was only observed for the binomial data with the logistic regression model as a base model. This is expected since this model is noncollapsible (see Section 2.4 for details).

For Setup B, the *Robinson* forests performed slightly better in disentangling the more complicated prognostic and predictive effects compared to *Naive* and ${\mathrm{Robinson}}_{\hat{W}}$ model-based forests. An exception is the binomial model: ${\mathrm{Robinson}}_{\hat{W}}$ forests performed similarly to *Robinson* forests.

In Setup C, over all distributions, uncentered model-based forests (*Naive*) failed to overcome the strong confounding effect and therefore did not provide accurate estimates for the treatment effect. The performance was fundamentally improved by centering the treatment indicator ( ${\mathrm{Robinson}}_{\hat{W}}$ ) and was further improved by additionally adding $\hat{m}(x)$ as an offset (*Robinson*).

In Setup D – with unrelated treatment and control arms – all methods had a higher mean squared error than in the other setups, as jointly modeling the expected conditional outcomes for both arms has no benefit. Apart from the normal distributions, *Robinson* forests were inferior to the ${\mathrm{Robinson}}_{\hat{W}}$ and *Naive* model-based forests.

The empirical evidence of our simulation study can be summarized as follows: If confounding was present, model-based forests performed better when centering W by $\hat{\pi }(x)$ ( ${\mathrm{Robinson}}_{\hat{W}}$ ) compared to not centering W (*Naive*). Adding $\hat{m}(x)$ as an offset (*Robinson*) further improved the performance – especially in cases with very strong confounding.

## 5. Effect of Riluzole on progression of ALS

ALS is a progressive nervous system disease causing loss of muscle control. The status of the disease as well as the rate of progression is commonly evaluated by the ALS functional rating scale (ALSFRS).^55,56^ Here, physical abilities such as speaking, handwriting, and walking are assessed and rated on a scale from 0 (inability) to 4 (normal ability). In 1995, the FDA approved the first drug to manage and slow progression of ALS, named Riluzole. The largest database for study results on the effect of Riluzole offers the Pooled Resource Open-Access Clinical Trials (PROACT) database – initiated by the non-profit organization Prize4Life (http://www.prize4life.org). The data comes from different randomized and observational studies not disclosed in the data. Thus, the assumption of random treatment assignment is quite hard to justify in an analysis. Patient characteristics and treatment group sizes might vary greatly between the centers, which affect both the probability of receiving treatment as well as the outcome. To account for these potential confounding effects, we compared the treatment effects estimated by the naive model-based forests to the ones estimated with local centering by Robinson. As in Section 4, we use random forests to estimate the propensity scores to center W and gradient boosting machines (with adapted loss functions) to estimate the values of the linear predictors $\eta_{0}(x)$ and $\eta_{1}(x)$ to center Y . Model-based forests, random forests, and gradient boosting machines rely on the hyperparameter values stated in Section 7. As for Seibold et al.^25^ and Korepanova et al.,^26^ 16 phase II and phase III randomized trials and one observational study from the PROACT database serve as a training dataset. We analyze the effect of Riluzole with respect to two outcome variables: Survival time and the handwriting ability score approximately 6 months after treatment—an item of the ALSFRS. We omitted observations with missing outcome values. As splitting variables, Seibold et al.^25^ used demographic, medical history, and family history data, which were informative in the sense that not more than half of their values were missing.

### 5.1. Survival Time

**Figure 2.**
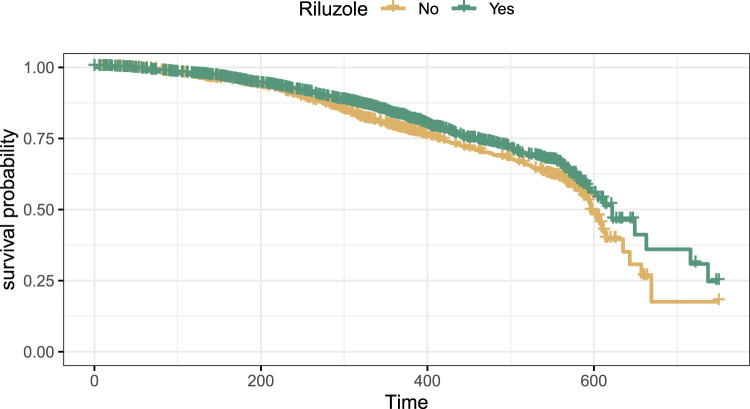
Kaplan-Meier curves of survival probability for both treatment arms.

The dataset for the survival time contains 3306 observations and 18 covariates. Of the 3306 observations, 2199 received Riluzole. Because very few patients had event times that exceed those of the others by a factor of two, we artificially censored five observations with (censoring or event) times of more than 750 days. The Kaplan-Meier estimates of survival probabilities for both treatment arms of the preprocessed dataset are shown in Figure 2. Overall, the estimated survival curves are very close to each other, and the treated group has only a slight survival advantage compared to the untreated group. As a base model, we use a Cox proportional hazards model. We compared treatment effects from two approaches: The naive uncentered model-based forests (*Naive*) and the model-based forest with Robinson’s orthogonalization (*Robinson*).

#### 5.1.1. Personalized models

For the naive model-based forests, the underlying Cox proportional hazards base model for the survival outcome T was, on the hazard scale,

$$\lambda (t)=\lambda_{0}(t)\exp\;(\mu +\tau w)$$

Because $\lambda_{0}(t)$ contains an intercept term, μ is not identified (and was constraint to μ≡0 ). The treatment effect τ is the log-hazard ratio of the treated versus untreated patients and our aim is to replace a constant marginal effect τ with a heterogeneous (and thus conditional) log-hazard ratio τ(x) and, simultaneously, to estimate prognostic effects μ(x) .

**Figure 3.**
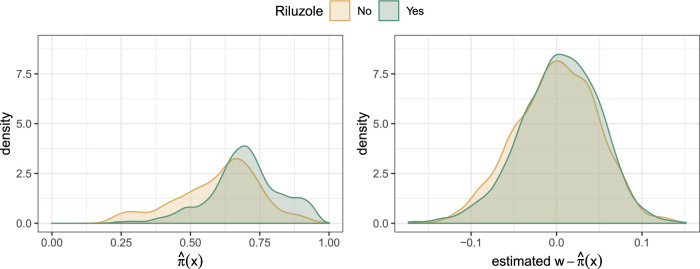
Distribution of estimated propensities $\hat{\pi }(x)$ (left) and estimated propensities of the centered treatment indicators (right, Robinson’s strategy) as estimated by regression forests for the two treatment groups.

For Robinson’s strategy, we first centered the treatment indicator W by estimating the propensity scores π(x)=P(W∣X=x) using a regression forest. Figure 3 compares the distributions of estimated propensity scores (left) and of the estimated centered treatment $W-\hat{\pi }(x)$ (Robinson’s strategy, right), both obtained from regression forests. We can already see a decent overlap of propensity scores in the two treatment arms without centering, but the overlap increases if the strategy by Robinson was applied.

In addition to centering W , Robinson’s strategy requires the estimation of m(x) to use as an offset (see Section 3). As in Section 4, we used gradient boosting machines (with the negative log partial likelihood of the Cox proportional hazards model as a loss) to estimate the natural parameters $\eta_{0}(x)$ and $\eta_{1}(x)$ for the control and treatment group, respectively.^57^ The offset m(x) for each observation is equal to the sum of natural parameter estimates weighted by $\hat{\pi }(x)$ (see equation (14)). The final base model for model-based forests using Robinson’s orthogonalization is

$$\lambda_{R}(t)=\lambda_{0}(t)\exp\;(\mu +\tau (w-\hat{\pi }(x))+\hat{m}(x))$$

#### 5.1.2. Model-based forests

**Figure 4.**
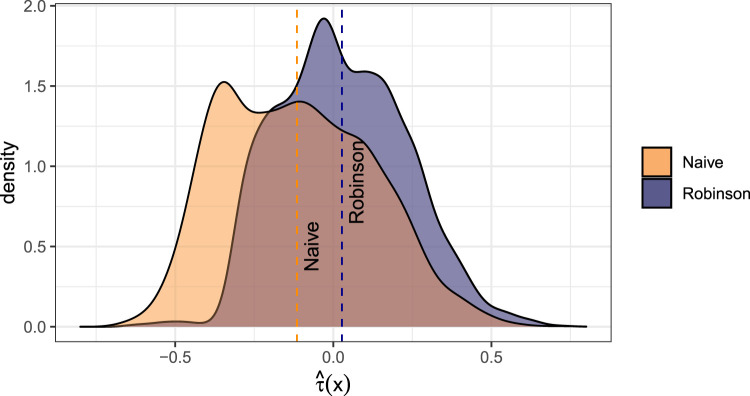
Kernel density estimates of the personalized treatment estimates for the naive model-based forest (*Naive*) and for the model-based forest with Robinson orthogonalization (*Robinson*).

The corresponding base models serve as an input for the model-based forests to estimate personalized effects of Riluzole. Figure 4 compares the kernel density estimates of τ(x) for each forest version (*Naive* and *Robinson*). The naive approach reveals that on average the treatment reduced the hazard compared to no treatment, whereas the model-based forest with centering according to Robinson obtained weaker effects of Riluzole with more mass centered around 0.

A meta-analysis of previous studies by Andrews et al.,^58^ also yielded a mixed picture: only eight of the 15 studies meeting their inclusion criteria showed a statistically significant increase of median survival time due to Riluzole.

Over all strategies, for both approaches there were some patients for which Riluzole was estimated to increase the hazard. The dependency plots in Figures S. 6 and S. 7 in the Supplemental Material provide indications of the characteristics of the group of harmed individuals. For example, both the naive and centering approach agree that for patients with atrophy or fasciculation, Riluzole intake would increase the hazard. The estimated effects differed most between the uncentered forest (*Naive*) and the orthogonalized forest (*Robinson*) for the covariate sex (Figure S. 6 (c)), the covariate of whether patients swallow, and for the covariate specifying whether cases in the same generation exist (Figure S. 7 (f) and (i)).

For the variables time onset treatment, age, height and weakness the dependency plots (Figure S. 6 (a), (d), (e) and Figure S. 7 (g)) of the *Naive* forest agree with the ones of Seibold et al.^25^: For middle-aged people with a longer time between disease onset and start of treatment, lower height, and no weakness, the treatment appears to be more beneficial. By considering confounding effects due to orthogonalization (*Robinson* forests), these effects diminished. For Korepanova et al.^26^ the effect of Riluzole was also rather weak and showed low heterogeneity across covariates.

### 5.2. Handwriting ability score

**Figure 5.**
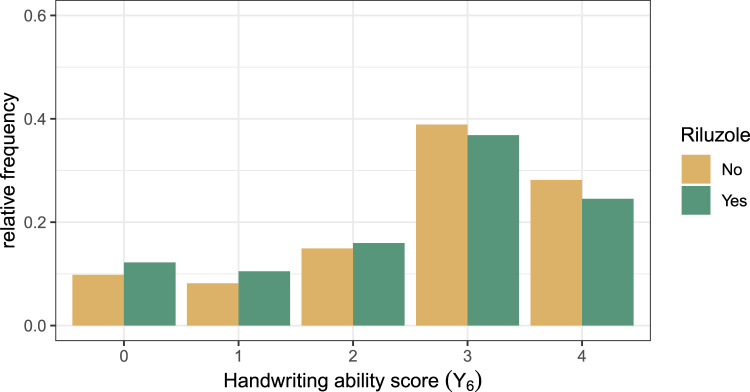
Relative frequency distribution plot of the handwriting ability score ( $Y_{6}$ ) (left) and of changes of the handwriting ability score over 6 months ( $Y_{6}-Y_{0}$ ) (right) for both treatment arms. Frequencies were calculated relative to the treatment indicator.

The dataset for the handwriting ability score—an ordinal outcome with five categories—contains 2538 observations and 58 covariates. Besides the covariate age, all covariates had missing values (but less than 50 % of the values were missing per variable enforced by the preprocessing step stated at the beginning of this section). Of the 2538 observations, 1754 received Riluzole, and 784 did not. Figure 5 displays the frequency of the ability scores for both treatment groups. Most of the patients have an ability score of 3 or 4 (normal ability); only a few have ability scores less than 2. Note that the plot shows the conditional proportions given the treatment indicator. We chose a proportional odds logistic regression model as a base model for the model-based forests—once without further adaptions (*Naive*), and once parameterized with centered W and with an offset (*Robinson*).

**Figure 6.**
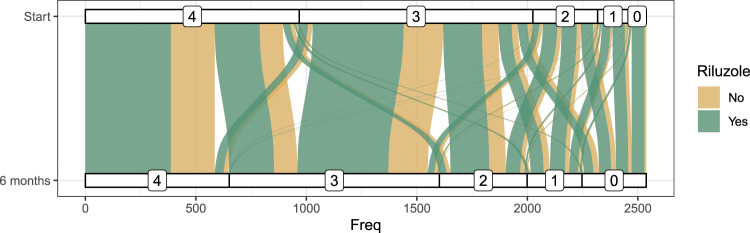
Alluvial plot of the progression of the handwriting ability score over 6 months for both treatment arms.

In addition to the handwriting ability score after 6 months, the ability score values at treatment start are also available. In the following, we denote $Y_{6}$ as the handwriting score after 6 months and $Y_{0}$ as the handwriting score at the beginning of the treatment period. To account for the ability level at treatment start, $Y_{0}$ served as an additional splitting variable for both model-based forests (*Naive* and *Robinson*) and was included in X . The alluvial plot in Figure 6 breaks down the change in each ability class over 6 months. Overall, for most patients, the handwriting ability remained constant over the 6 months or worsened slightly. Rarely, patients experienced a progression to both extremes (0 to 4, or 4 to 0). These results hold regardless of whether patients received Riluzole or not.

#### 5.2.1. Personalized models

The proportional odds logistic regression model for the naive model-based forests is defined as^59,60^

$$\mathrm{logit}\;(P(Y_{6}\leq k|X=x,W=w,Y_{0}=y_{0}))=\vartheta_{k}(x,y_{0})-\tau (x,y_{0})w$$

with k∈{0,…,3} as the ordinal ability score classes. The parameters $\vartheta_{k}$ are increasing thresholds, depending on covariates x and the initial score $y_{0}$ . Due to the proportional odds assumption, the treatment effect $\tau (x,y_{0})$ is the same for all scores k . Negative $\tau (x,y_{0})$ indicate a negative effect of Riluzole, as treated patients are expected to have a higher odds of low writing ability scores compared to untreated patients.

We first applied regression forests to estimate propensity scores $\pi (x,y_{0})$ and a gradient boosting machine (with adapted loss functions for the proportional odds model) to estimate the natural parameters $\eta_{0}(x,y_{0})$ and $\eta_{1}(x,y_{0})$ . The personalized model for the model-based forest with Robinson orthogonalization was specified as

$$\mathrm{logit}\;(P(Y_{6}\leq k|X=x,W=w,Y_{0}=y_{0}))=\vartheta_{k}(x,y_{0})-[\hat{m}(x,y_{0})+\tau (x,y_{0})\{w-\hat{\pi }(x,y_{0})\}]$$

with $\hat{m}(x,y_{0})$ as defined in equation (14).

**Figure 7.**
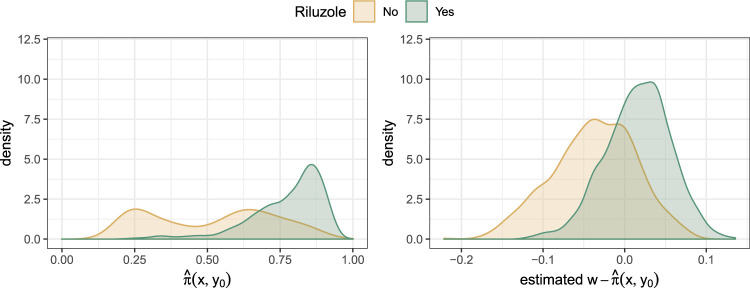
Estimates returned by the regression forest for orthogonalization of the treatment indicator: left for original W as an outcome in the random forest such that it estimates propensity scores $\pi (x,y_{0})$ ; right for the centered treatment indicator $W-\hat{\pi }(x,y_{0})$ as an outcome in the random forest.

Figure 7 compares the estimated treatment indicators with W as the outcome in the random forest without centering (left), with $\left(W-\hat{\pi }(x,y_{0})\right)$ as the outcome in the random forest (right). Before centering, there is a lack of overlap of the propensity scores; the distribution of $\hat{\pi }$ for the control group is bimodal, and the distribution for the treatment group is heavily left-skewed. After centering, the distributions of the estimated $W-\hat{\pi }(x,y_{0})$ for the treatment groups move closer together and have a similar unimodal shape. However, there is still a lack of overlap of the groups, which indicates that important covariates to explain the remaining heterogeneity in the two treatment groups seem to be missing.

#### 5.2.2. Model-based forests

**Figure 8.**
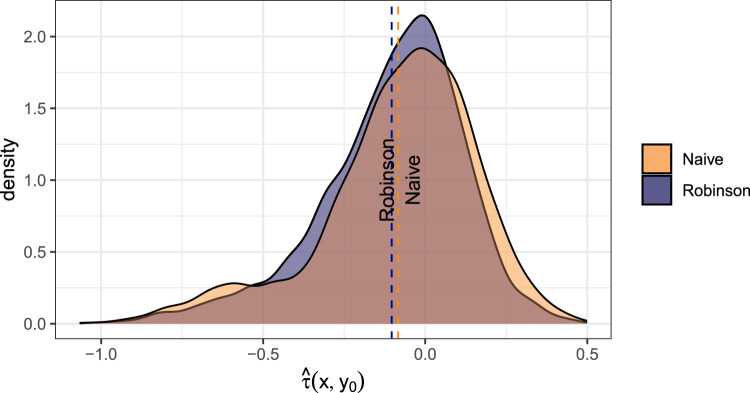
Kernel density estimates of the personalized treatment estimates for the naive model-based forest (*Naive*) vs. the forest with Robinson orthogonalized (*Robinson*).

The proportional odds logistic regression models served as a base model for the (*Naive* and *Robinson*) model-based forests to derive personalized treatment effects. Figure 8 displays the kernel density estimates of $\tau (x,y_{0})$ for each forest version (*Naive* and *Robinson*).

Both random forests estimate on average a negative effect of Riluzole. Naive model-based forest estimated on average a log-odds of $\bar{\tau }=-0.08$ , which indicates that treated patients have a 0.08 points higher log-odds for low writing scores than untreated patients. The distribution of $\hat{\tau }(x,y_{0})$ for the model-based forest relying on the Robinson orthogonalization is slightly shifted to the left ( $\bar{\tau }=-0.10$ ). For a larger subgroup of patients, the naive approach estimates a negative effect of Riluzole ( $-1\leq \tau (x,y_{0})\leq -0.5$ ), meaning that patients receiving treatment with Riluzole have higher odds of low writing scores than untreated patients. According to the dependency plots (Figures S. 8 to S. 13 in the Supplemental Material), this subgroup could be identified as having low initial ability scores (left side of Figure S. 8 (a)). For all other splitting variables, the distributions of estimated treatment effects are very similar.

## 6. Discussion and outlook

HTE estimation is a challenging problem, especially for observational studies and even more when the outcome cannot be modeled by a linear model. Covering diverse outcomes is not straightforward due to the noncollapsibility issue. Measures like the hazard ratios are shrunken towards zero and are incomparable between individuals if the full model including prognostic effects is not estimated. Model-based forests allow for the full model estimation given randomized trial data. In this work, we investigated several versions of model-based forests for the estimation of potentially complex HTEs τ(x) based on observational data with various outcome types based on the orthogonalization strategy by Robinson.^34^ These investigations suggest the following workflow for model-based forests: (1) Estimate propensities π(x) using some machine learning procedure (binary random forests are a good default), (2) center the treatment indicator $w-\hat{\pi }(x)$ for each observation, (3) set up an appropriate model for the outcome conditioning on the centered treatment and – if possible – add an offset for centering Y , (4) use model-based forests to estimate predictive and prognostic effects τ(x) and μ(x) simultaneously. Notably, τ(x) is the CATE only in specific models, especially a linear or log-linear model. We demonstrate these steps by estimating the individual effects of Riluzole for ALS patients using survival times and ordinal ability scores as outcomes.

Many conceptual and technical issues remain to be addressed, for example the question how model-based forests perform for survival data for which the censoring procedure is not randomized but depends on X , or how ( k -fold) cross-fitting influences the performance, where only one part of the data is used to estimate nuisance parameters and the other part to estimate τ(x) .^15^ We leave investigations to these questions to future research.

Last but not least, we want to emphasize that all approaches for estimating HTEs – including those presented in this work – rely on strong and typically untestable assumptions. For example, for models beyond mean regression, $\hat{\tau }(x)$ cannot be expected to be robust against missing covariates or other violations of model assumptions due to noncollapsibility. Consequently, results from these approaches in practical applications should be evaluated with the utmost caution, reservation, and humility.

## 7. Computational details

For all computations, we used R version 4.1.1,^61^ with the following add-on packages: model4you,^62^ trtf,^63^ partykit,^64^ grf,^52^ mboost,^53^ and gbm.^54^

Model-based forests were always grown with M =500 trees (model4you::pmforest default) with a minimum node size of node =14 , number of chosen variables per split mtry =P , and subsampling. These settings were also used by Dandl et al.^36^ Transformation forests implemented in the trtf package fitted the Weibull transformation forests of Section 4.^63,29^

Propensity scores π(x) were estimated with grf (honest) regression forests with 125 trees, a minimum node size of 5, and subsampling. Natural parameters $\eta_{0}(x)$ and $\eta_{1}(x)$ and probability of not being censored were estimated with gradient boosting machines implemented in the mboost or gbm packages. The used maximum tree depth was 2 (default of mboost::blackboost), and a loss function that differed depending on the outcome type was also employed.^53,54^

Ratios and confidence intervals presented in Table 2 were calculated using generalized linear mixed models of the glmmTMB package.^65^ Post hoc inference relied on the multcomp package.^66^

All study settings are available in a dedicated R package called htesim.^67^ It is published on Github: https://github.com/dandls/htesim.

## Supplemental Material

sj-pdf-1-smm-10.1177_09622802231224628 - Supplemental material for Heterogeneous treatment effect estimation for observational data using model-based forests Supplemental material, sj-pdf-1-smm-10.1177_09622802231224628 for Heterogeneous treatment effect estimation for observational data using model-based forests by Susanne Dandl, Andreas Bender and Torsten Hothorn in Statistical Methods in Medical Research
