# Supplementary material for: Heterogeneous treatment effect estimation for observational data using model-based forests
Source: Stat Methods Med Res. 2024 Feb 8;33(3):392–413. doi: 10.1177/09622802231224628 (PMC10981193; doi:10.1177/09622802231224628)
Supplement: sj-pdf-1-smm-10.1177_09622802231224628 - Supplemental material for Heterogeneous treatment effect estimation for observational data using model-based forests [file sj-pdf-1-smm-10.1177_09622802231224628.pdf]

Supplementary Material: Heterogeneous Treatment Effect Estimation for Observational Data using Model-Based Forests

Susanne Dandl
LMU Munich, MCML

Andreas Bender
LMU Munich, MCML

Torsten Hothorn
Universität Zürich

Abstract

Additional simulation study results including statistical analyses, as well as dependence plots for the application example displaying the effect of Riluzole on progression of ALS.

Keywords: Heterogeneous treatment effects, personalized medicine, random forest, observational data, censored survival data, generalized linear model, transformation model.

A. Noncollapsibility

As mentioned in Section 2.4, for members of the exponential family without an identity or linear link function the marginal and conditional treatment effects are not collapsible. This means that the mean of the conditional treatment effects given a covariate are not equal to the marginal treatment effect estimate over the same covariate (Greenland, Pearl, and Robins 1999). This happens if the covariate conditioned on is associated with the outcome of interest. Caution is necessary on multiple stages of the estimation process of $\tau(\boldsymbol{x})$ as soon as we condition on other covariates, for example, because these covariates are assumed to be sufficient to control for confounding (Daniel, Zhang, and Farewell 2021).

In case of Robinson's orthogonalization, misspecification of $m(\boldsymbol{x})$ translates into biased estimators for $\tau(\boldsymbol{x})$, even under randomized treatments. This also applies if one ignores the estimation of $\mu(\boldsymbol{x})$ at all and only concentrates on $\tau(\boldsymbol{x})$. This is not the case for the linear model (identity link function) since misspecifications are absorbed in the additive error term and do not influence the estimation of $\tau(\boldsymbol{x})$ (Gao and Hastie 2022).

A.1. Review Gao and Hastie (2022)

Gao and Hastie (2022) extended the orthogonalization strategy of Robinson (1988) to improve robustness to both confounding and noncollapsibility. The authors propose

$$a(\boldsymbol{x}) = \frac{\pi(\boldsymbol{x})\frac{\partial\gamma(\eta_1(\boldsymbol{x}))}{\partial\eta}}{\pi(\boldsymbol{x})\frac{\partial\gamma(\eta_1(\boldsymbol{x}))}{\partial\eta} + (1-\pi(\boldsymbol{x}))\frac{\partial\gamma(\eta_0(\boldsymbol{x}))}{\partial\eta}} \tag{1}$$

and

$$\nu(\boldsymbol{x}) = a(\boldsymbol{x})n_1(\boldsymbol{x}) + (1-a(\boldsymbol{x}))n_0(\boldsymbol{x})$$

instead of $\pi(\boldsymbol{x})$ (equation (9)) and $m(\boldsymbol{x})$ (equation (14)), respectively, where $\gamma(\eta)$ denotes the inverse of the canonical link function. Its derivative is equal to the variance function of the exponential family. Therefore, $a(\boldsymbol{x})$ is larger if an observation is likely to be treated (which also holds for Robinson's orthogonalization) or if the response variance is higher under treatment compared to no treatment. As a consequence of the latter, the influence of spuriously influential natural parameter values is reduced for more robustness to misspecifications (Gao and Hastie 2022).

For Gaussian responses, $a(\boldsymbol{x}) = \pi(\boldsymbol{x})$ and $\nu(\boldsymbol{x}) = m(\boldsymbol{x})$ holds, while for other distributions the terms differ. For example, for Bernoulli distributed Y, the closed form $a(\boldsymbol{x})$ is

$$a(\boldsymbol{x}) = \frac{\pi(\boldsymbol{x})}{\pi(\boldsymbol{x}) + (1-\pi(\boldsymbol{x}))\frac{p_0(\boldsymbol{x})(1-p_0(\boldsymbol{x}))}{p_1(\boldsymbol{x})(1-p_1(\boldsymbol{x}))}} \tag{2}$$

where $p_w(\boldsymbol{x}) = \mathbb{P}(Y = 1|\boldsymbol{X} = \boldsymbol{x}, W = w)$.

The noncollapsibility issue is not only present for distributions of the exponential family. Also the Cox model suffers from noncollapsibility (Greenland 1996; Aalen, Cook, and Røysland 2015). This is in contrast to accelerated failure time models (such as the Weibull proportional hazards model), which can be rewritten as location-scale models and therefore are indeed collapsible (Aalen *et al.* 2015). For the Cox model, Gao and Hastie remark that with knowledge of the baseline hazard function and without censoring, the cumulative hazard function follows an exponential distribution. For the exponential distribution, $a(\boldsymbol{x})$ and $\nu(\boldsymbol{x})$ are equal to $\pi(\boldsymbol{x})$ and $m(\boldsymbol{x})$ (Gao and Hastie 2022).

In case of random censoring, the probability of not being censored under both treatment arms needs to be considered for the estimation of $a(\boldsymbol{x})$ and $\nu(\boldsymbol{x})$

$$a(\boldsymbol{x}) = \frac{\pi(\boldsymbol{x})\mathbb{P}(C \geq Y|\boldsymbol{X} = \boldsymbol{x}, W = 1)}{\pi(\boldsymbol{x})\mathbb{P}(C \geq Y|\boldsymbol{X} = \boldsymbol{x}, W = 1) + (1-\pi(\boldsymbol{x}))\mathbb{P}(C \geq Y|\boldsymbol{X} = \boldsymbol{x}, W = 0)} \tag{3}$$

$$\nu(\boldsymbol{x}) = a(\boldsymbol{x})\eta_1(\boldsymbol{x}) + (1 - a(\boldsymbol{x}))\eta_0(\boldsymbol{x}). \tag{4}$$

The nuisance parameter $a(\boldsymbol{x})$ is larger if an observation is likely to be treated or likely to be not censored. Consequently, the influence of likely to be not censored observations for the estimation of $\tau(\boldsymbol{x})$ is increased. Above's $a(\boldsymbol{x})$ and $\nu(\boldsymbol{x})$ guarantee protection to misspecified nuisance parameter if the baseline hazard is known. If it is unknown and the partial likelihood is used – this is not guaranteed. Despite this lack of guarantee, Gao and Hastie, 2022, obtained promising results in their simulation study (Gao and Hastie 2022).

A.2. Strategies against confounding and noncollapsibility

An interesting question is if replacing $\hat{\pi}(\boldsymbol{x})$ and $\hat{m}(\boldsymbol{x})$ by $\hat{a}(\boldsymbol{x})$ and $\hat{\nu}(\boldsymbol{x})$, respectively, also helps to additionally tackle noncollapsibility when applying model-based forests. We can update the linear predictor for model-based forests in case of generalized linear models to

$$g(\mathbb{E}(Y \mid \boldsymbol{X} = \boldsymbol{x}, W = w)) = \hat{\nu}(\boldsymbol{x}) + \tilde{\mu}(\boldsymbol{x}) + \tau(\boldsymbol{x})(w - \hat{a}(\boldsymbol{x})).$$

Gao and Hastie additionally derived estimators for $a(\boldsymbol{x})$ and $\nu(\boldsymbol{x})$ for the Cox model which – compared to the Weibull model – is not collapsible. For the Cox model, the natural parameter of equation (8) could be updated to

$$\eta_w(\boldsymbol{x}) = \hat{\nu}(\boldsymbol{x}) + \tau(\boldsymbol{x})(w - \hat{a}(\boldsymbol{x}))$$

with $a(\boldsymbol{x})$ and $\nu(\boldsymbol{x})$ as defined in equations (3) and (4).

We call this version of model-based forests in the following *Gao* approach. Before we apply model-based forests, we need to estimate $\pi(\boldsymbol{x})$, $\eta_0(\boldsymbol{x})$, $\eta_1(\boldsymbol{x})$ as well as $\frac{\partial v(\eta_1(\boldsymbol{x}))}{\partial \eta}$ for exponential families and $\mathbb{P}(C \geq Y|\boldsymbol{X} = \boldsymbol{x}, W = w)$ for Cox models. As in Section 3.3, we state some research questions that are empirically inspected in the upcoming section.

RQ 4: How do model-based forests centered according to Gao and Hastie (*Gao*) perform compared to model-based forest with Robinson strategy (*Robinson*) for the simulation settings of Section 4?

Similar to RQ 2, we could solely center W by $a(\boldsymbol{x})$ without including an offset. We call this approach $Gao_{\hat{W}}$ in the following.

RQ 5: How do model-based forest with solely centered W by $\hat{a}(\boldsymbol{x})$ ($Gao_{\hat{W}}$) perform compared to model-based forests with solely centered W by $\hat{\pi}(\boldsymbol{x})$ $Robinson_{\hat{W}}$ for the simulation study settings of Section 4?

Table S. 1: Updated overview of proposed model-based forest versions (Table 1) for observational data.

Method	Linear Predictor	Definitions
Naive	$\mu(\boldsymbol{x}) + \tau(\boldsymbol{x})\ w$	
$Robinson_{\hat{W}}$	$\mu(\boldsymbol{x}) + \tau(\boldsymbol{x})(w - \hat{\pi}(\boldsymbol{x}))$	$\pi(\boldsymbol{x}) = \mathbb{P}(W = 1 \vert \boldsymbol{X} = \boldsymbol{x})$
Robinson	$\tilde{\mu}(\boldsymbol{x}) + \tau(\boldsymbol{x})(w - \hat{\pi}(\boldsymbol{x})) + \hat{m}(\boldsymbol{x})$	$m(\boldsymbol{x}) = \pi(\boldsymbol{x})\eta_1(\boldsymbol{x}) - (1 - \pi(\boldsymbol{x}))\eta_0(\boldsymbol{x})$
$Gao_{\hat{W}}$	$\mu(\boldsymbol{x}) + \tau(\boldsymbol{x})(w - \hat{a}(\boldsymbol{x}))$	$a(\boldsymbol{x}) = \frac{\pi(\boldsymbol{x})\frac{\partial\gamma(\eta_1(\boldsymbol{x}))}{\partial\eta}}{\pi(\boldsymbol{x})\frac{\partial\gamma(\eta_1(\boldsymbol{x}))}{\partial\eta} + (1-\pi(\boldsymbol{x}))\frac{\partial\gamma(\eta_0(\boldsymbol{x}))}{\partial\eta}}$
Gao	$\tilde{\mu}(\boldsymbol{x}) + \tau(\boldsymbol{x})(w - \hat{a}(\boldsymbol{x})) + \hat{\nu}(\boldsymbol{x})$	$\nu(\boldsymbol{x}) = a(\boldsymbol{x})n_1(\boldsymbol{x}) + (1 - a(\boldsymbol{x}))n_0(\boldsymbol{x})$

Note: for the Cox model $a(\boldsymbol{x}) = \frac{\pi(\boldsymbol{x})\mathbb{P}(C \geq Y|\boldsymbol{X}=\boldsymbol{x}, W=1)}{\pi(\boldsymbol{x})\mathbb{P}(C \geq Y|\boldsymbol{X}=\boldsymbol{x}, W=1) + (1-\pi(\boldsymbol{x}))\mathbb{P}(C \geq Y|\boldsymbol{X}=\boldsymbol{x}, W=0)}$ is used.

A.3. Data-generating process

To investigate the research questions of Section A.2., we compared the performance of model-based forests with Gao's strategy proposed in Section A.2 (*Gao* and $Gao_{\hat{W}}$) to model-based forests with Robinson's startegy (*Robinson* and $Robinson_{\hat{W}}$) for settings A, B, C, D described in Section 4. Because we expect that the strategy of Gao is especially valuable for settings with misspecified prognostic effect, e.g. because prognostic covariates are missing, we additionally created Setup A' from Setup A by removing covariate $\boldsymbol{X}_3$ from the training data. Therefore, the DGP of Setup A and Setup A' are identical, the only difference being that the training data did not contain $\boldsymbol{X}_3$ although $\boldsymbol{X}_3$ affects the prognostic effect.

Because the normal linear model and Weibull model are collapsible and Gao's strategy is equal to Robinson's strategy (Sections 2.4 and A.1), we applied our proposed approaches based on Gao and Hastie (2022) only to the binomial model and the Cox model. Transformation models such as the proportional odds model for multinomial data were not covered by the authors.

We used the same model-based forest parameter setup and evaluation scheme as in Section 4.

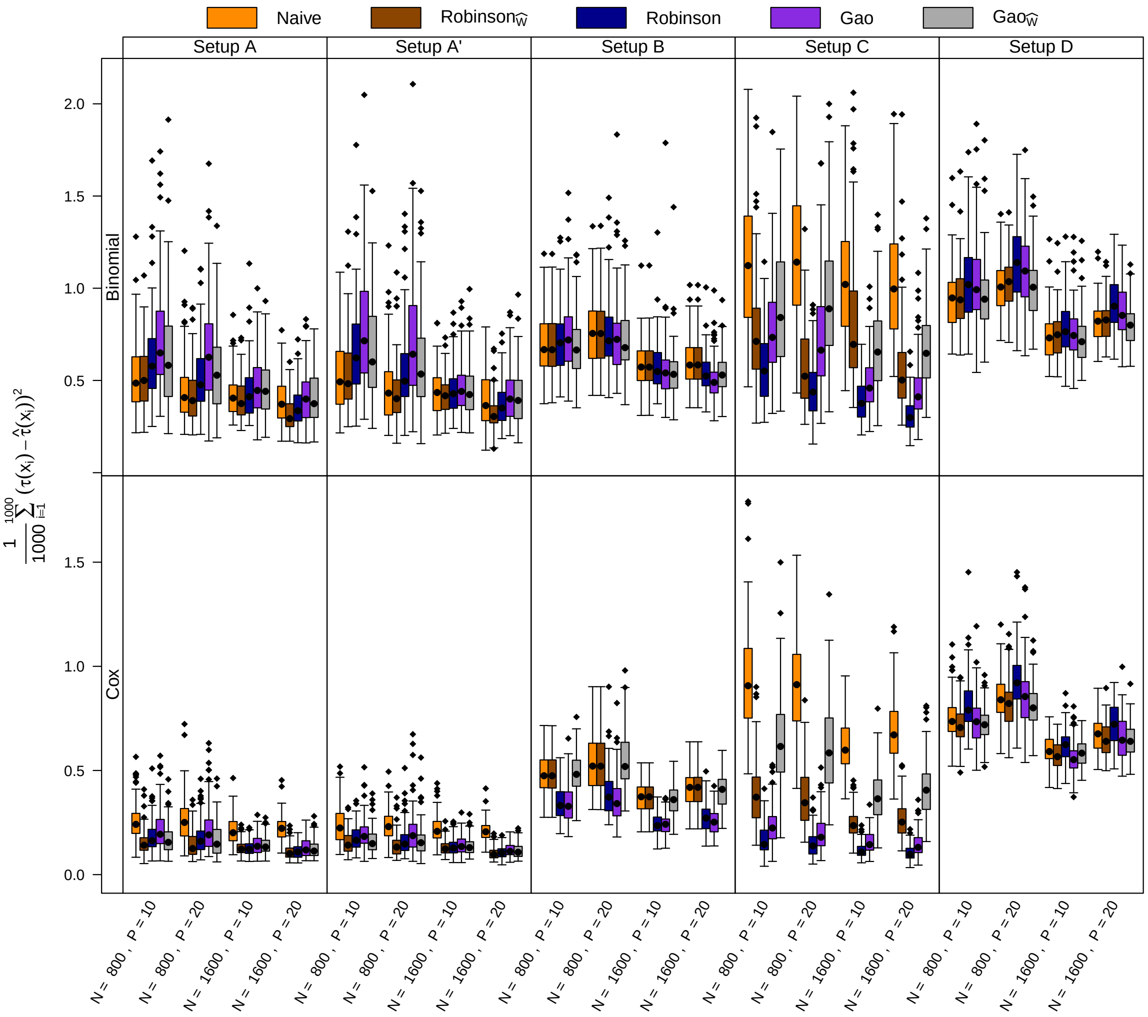

Figure S. 1: Model-based forest results for the empirical study (Section 4), Cox means a Cox model applied to the Weibull data. For the Cox model, treatment effects $\tau(\boldsymbol{x})$ are estimated as conditional log hazard ratios. Direct comparison of model-based forests without centering (Naive), model-based forests with local centering according to Robinson (1988) or Gao and Hastie (2022) of Y and W (originally proposed) (*Robinson*, *Gao*) or only of W (*Robinson*$_{\widehat{W}}$, *Gao*$_{\widehat{W}}$).

Table S. 2: Results of **RQ 4** for the experimental setups in Section 4. Comparison of mean squared errors for $\hat{\tau}(\boldsymbol{x})$ in the different scenarios. Estimates and simultaneous 95 % confidence intervals were obtained from a normal linear mixed model with log-link. Cells printed in bold font correspond to a superior reference of *Robinson* forests, cells printed in italics indicate an inferior reference.

			Mean squared error ratio for RQ 4: Gao vs. Robinson	
DGP	N	P	Binomial	Cox
Setup A	800	10	**1.258 (1.152, 1.373)**	**1.203 (1.077, 1.344)**
		20	**1.307 (1.180, 1.449)**	**1.307 (1.170, 1.461)**
	1600	10	1.067 (0.933, 1.220)	1.121 (0.947, 1.326)
		20	**1.183 (1.009, 1.387)**	1.155 (0.955, 1.398)
Setup A'	800	10	**1.201 (1.105, 1.304)**	**1.140 (1.011, 1.285)**
		20	**1.354 (1.233, 1.487)**	**1.272 (1.127, 1.435)**
	1600	10	1.047 (0.915, 1.199)	1.055 (0.895, 1.243)
		20	**1.184 (1.014, 1.382)**	1.114 (0.911, 1.362)
Setup B	800	10	1.042 (0.958, 1.134)	0.984 (0.920, 1.052)
		20	0.987 (0.909, 1.073)	*0.906 (0.853, 0.963)*
	1600	10	0.987 (0.885, 1.100)	0.977 (0.889, 1.074)
		20	0.926 (0.824, 1.042)	0.922 (0.845, 1.006)
Setup C	800	10	**1.388 (1.263, 1.524)**	**1.417 (1.261, 1.592)**
		20	**1.616 (1.448, 1.804)**	**1.401 (1.228, 1.598)**
	1600	10	**1.276 (1.104, 1.476)**	**1.360 (1.146, 1.615)**
		20	**1.485 (1.255, 1.758)**	**1.400 (1.163, 1.686)**
Setup D	800	10	0.996 (0.939, 1.057)	*0.916 (0.889, 0.943)*
		20	0.965 (0.913, 1.020)	*0.925 (0.902, 0.949)*
	1600	10	0.964 (0.890, 1.044)	*0.910 (0.875, 0.946)*
		20	0.948 (0.884, 1.015)	*0.907 (0.877, 0.938)*

Table S. 3: Results of **RQ 5** for the experimental setups in Section 4. Comparison of mean squared errors for $\hat{\tau}(\boldsymbol{x})$ in the different scenarios. Estimates and simultaneous 95 % confidence intervals were obtained from a normal linear mixed model with log-link. Cells printed in bold font correspond to a superior reference of $\textit{Robinson}_{\hat{W}}$ forests, cells printed in italics indicate an inferior reference.

			Mean squared error ratio for RQ 5: $\text{Gao}_{\hat{W}}$ vs. $\text{Robinson}_{\hat{W}}$	
DGP	N	P	Binomial	Cox
Setup A	800	10	**1.299 (1.168, 1.445)**	1.127 (0.986, 1.288)
		20	**1.425 (1.255, 1.618)**	**1.190 (1.038, 1.366)**
	1600	10	**1.162 (1.009, 1.339)**	1.110 (0.940, 1.310)
		20	**1.339 (1.128, 1.589)**	1.144 (0.944, 1.386)
Setup A'	800	10	**1.261 (1.139, 1.397)**	1.096 (0.952, 1.263)
		20	**1.427 (1.264, 1.610)**	**1.195 (1.033, 1.382)**
	1600	10	1.096 (0.950, 1.264)	1.060 (0.896, 1.255)
		20	**1.305 (1.101, 1.548)**	1.114 (0.906, 1.370)
Setup B	800	10	0.988 (0.904, 1.079)	1.005 (0.959, 1.053)
		20	0.959 (0.883, 1.042)	1.037 (0.995, 1.081)
	1600	10	0.947 (0.849, 1.056)	0.968 (0.910, 1.031)
		20	0.905 (0.811, 1.009)	0.982 (0.929, 1.038)
Setup C	800	10	**1.228 (1.141, 1.323)**	**1.636 (1.561, 1.715)**
		20	**1.658 (1.524, 1.804)**	**1.585 (1.510, 1.664)**
	1600	10	*0.716 (0.660, 0.776)*	**1.552 (1.437, 1.677)**
		20	**1.272 (1.149, 1.408)**	**1.588 (1.481, 1.702)**
Setup D	800	10	1.011 (0.948, 1.079)	1.004 (0.973, 1.037)
		20	0.981 (0.923, 1.042)	0.987 (0.960, 1.016)
	1600	10	0.969 (0.891, 1.054)	1.027 (0.987, 1.069)
		20	0.970 (0.899, 1.048)	1.003 (0.968, 1.039)

A.4. Results

For Setup A, solely centering W by $\hat{a}(\boldsymbol{x})$ ($Gao_{\hat{W}}$) achieved better results than additionally adding the offset $\hat{\nu}(\boldsymbol{x})$ (Gao). Model-based forests with Robinson's strategy (*Robinson*, $Robinson_{\hat{W}}$) overall performed better than model-based forests with Gao's strategy (Gao, $Gao_{\hat{W}}$). Surpressing X_3 in the training dataset (Setup A'), did not deteriorate the performance of all methods such that the ranking of methods was retained.

For Setup B, model-based forests centered by *Gao* and *Robinson* model-based forests performed akin for binary outcomes. Also $Robinson_{\hat{W}}$ and $Gao_{\hat{W}}$ model-based forests achieved similar performance.

In Setup C, Gao's strategy for the Cox and logistic regression model overall fare worse than Robinson's strategy. In Setup D, $Gao_{\hat{W}}$ forests performed as good as $Robinson_{\hat{W}}$ forests for the Cox and logistic regression models. Notably, for the Cox model, *Gao* forests outperformed *Robinson* forests.

Overall, the orthogonalization strategy of Gao for the exponential family – that aims at addressing the noncollapsibility issue – did not perform as well as expected. Our expectation was that the strategy would reduce the effect of overfitting the marginal effect $\hat{m}(\boldsymbol{x})$ on the treatment effect estimate. Overall, however, the estimation of additional nuisance parameters tended to worsen the performance results on average – at least for the binomial model. For the Cox model, Gao's strategy, which additionally takes the probability for not getting censored into account, did not worsen performance. Further experiments are necessary in which the censoring probability is not constant but depends on covariates $\boldsymbol{x}$.

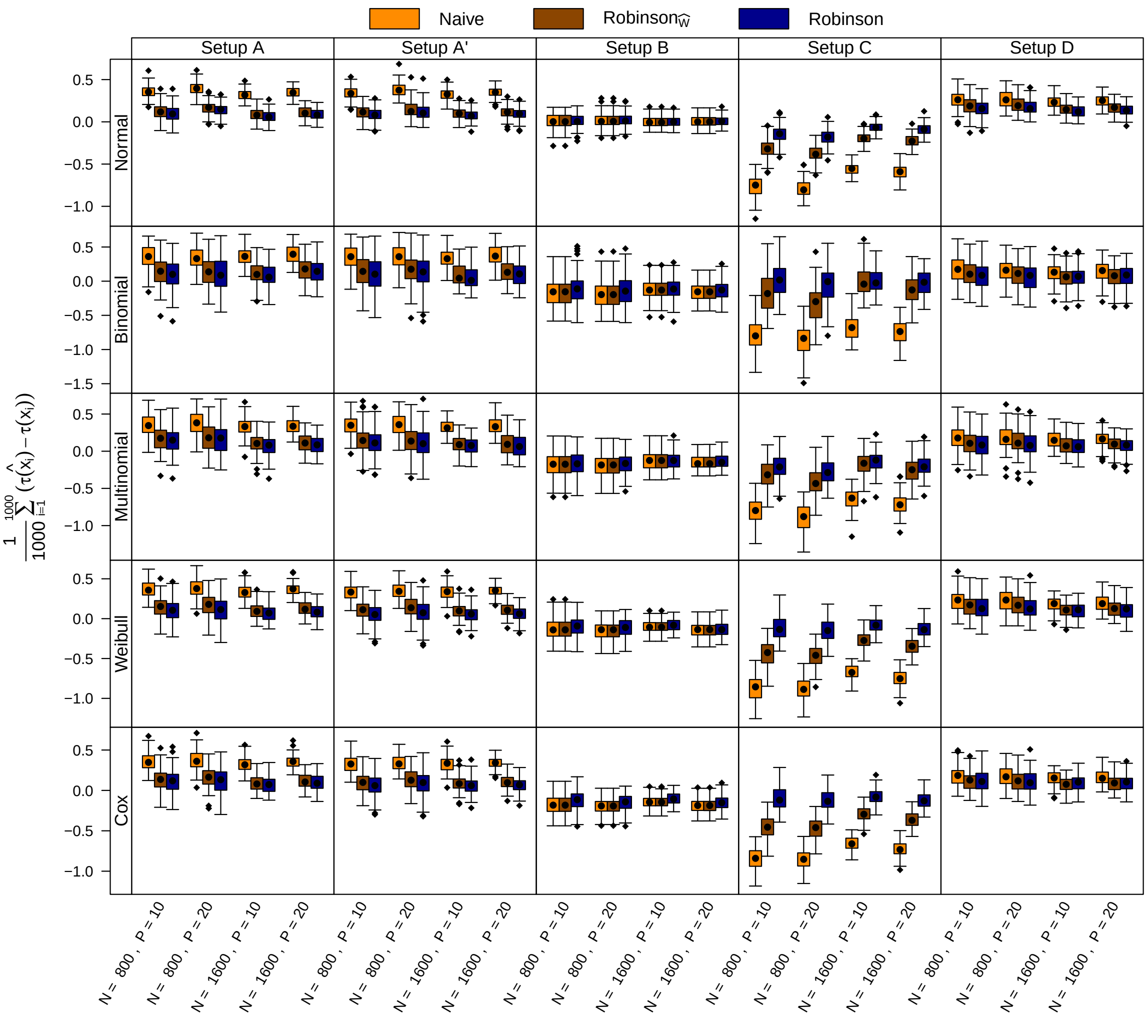

Figure S. 2: Model-based forest results w.r.t. bias for the empirical study (Section 4), Cox means a Cox model applied to the Weibull data. For the Weibull and Cox model, treatment effects $\tau(\boldsymbol{x})$ are estimated as conditional log hazard ratios. Direct comparison of model-based forests without centering (*Naive*), model-based forests with local centering according to Robinson (1988) of Y and W (originally proposed) (*Robinson*) or only of W (*Robinson*$_{\widehat{W}}$).

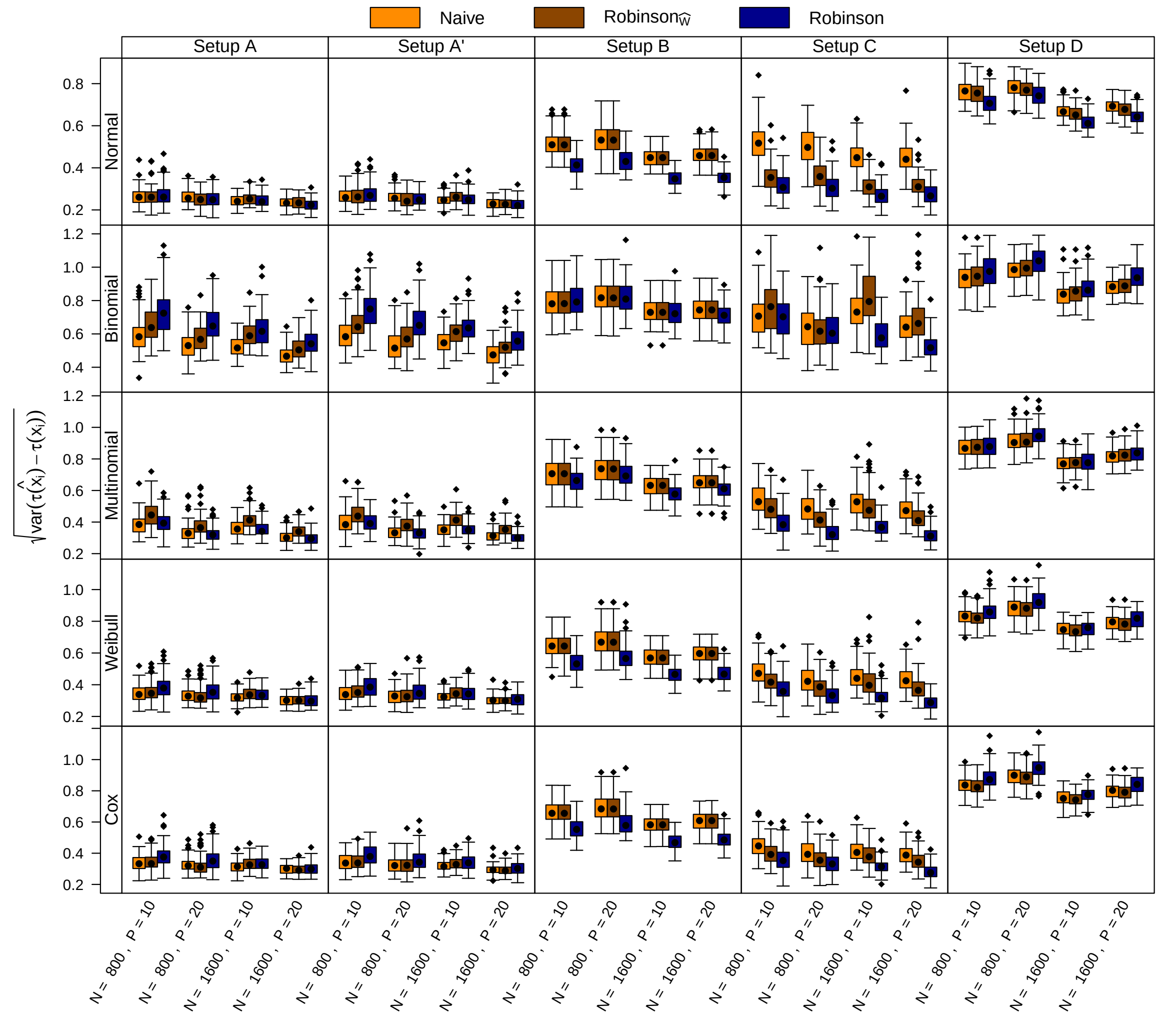

Figure S. 3: Model-based forest results w.r.t. the standard error for the empirical study (Section 4), Cox means a Cox model applied to the Weibull data. For the Weibull and Cox model, treatment effects $\tau(\boldsymbol{x})$ are estimated as conditional log hazard ratios. Direct comparison of model-based forests without centering (*Naive*), model-based forests with local centering according to Robinson (1988) of Y and W (originally proposed) (*Robinson*) or only of W (*Robinson*$_{\widehat{W}}$).

Table S. 4: Results of **RQ 1** for the experimental setups in Section 4. Comparison based on the relative efficiency for $\hat{\tau}(\boldsymbol{x})$ in the different scenarios. Estimates and simultaneous 95 % confidence intervals were obtained from a normal linear mixed model with log-link. Cells printed in bold font correspond to a superior reference of the *Naive* model-based forests, and cells printed in italics indicate an inferior reference.

			Mean squared error ratio for **RQ 1**: Robinson vs. Naive				
DGP	N	P	Normal	Binomial	Multinomial	Weibull	Cox
Setup A	800	10	1.080 (0.958, 1.217)	**1.552 (1.398, 1.724)**	1.088 (0.980, 1.207)	**1.271 (1.123, 1.439)**	**1.302 (1.156, 1.467)**
		20	0.959 (0.841, 1.093)	**1.563 (1.371, 1.783)**	0.907 (0.783, 1.051)	**1.227 (1.071, 1.406)**	**1.275 (1.120, 1.452)**
	1600	10	1.015 (0.874, 1.179)	**1.499 (1.304, 1.724)**	0.957 (0.841, 1.088)	1.139 (0.976, 1.329)	1.126 (0.971, 1.307)
		20	0.916 (0.772, 1.085)	**1.376 (1.152, 1.644)**	0.950 (0.794, 1.137)	1.015 (0.846, 1.219)	1.026 (0.862, 1.222)
Setup A'	800	10	1.113 (0.989, 1.253)	**1.598 (1.443, 1.770)**	1.000 (0.903, 1.107)	**1.254 (1.112, 1.415)**	**1.286 (1.146, 1.444)**
		20	0.961 (0.843, 1.095)	**1.633 (1.436, 1.858)**	0.977 (0.845, 1.130)	**1.233 (1.071, 1.419)**	**1.271 (1.113, 1.452)**
	1600	10	1.042 (0.903, 1.201)	**1.351 (1.186, 1.539)**	1.002 (0.877, 1.143)	**1.173 (1.013, 1.358)**	**1.175 (1.019, 1.355)**
		20	0.961 (0.809, 1.142)	**1.428 (1.205, 1.693)**	0.940 (0.789, 1.120)	1.094 (0.911, 1.313)	1.109 (0.931, 1.320)
Setup B	800	10	*0.638 (0.611, 0.667)*	1.029 (0.959, 1.103)	*0.866 (0.836, 0.897)*	*0.687 (0.654, 0.722)*	*0.708 (0.678, 0.740)*
		20	*0.654 (0.629, 0.679)*	0.984 (0.921, 1.051)	*0.906 (0.878, 0.935)*	*0.709 (0.679, 0.740)*	*0.736 (0.708, 0.764)*
	1600	10	*0.598 (0.563, 0.635)*	0.979 (0.901, 1.064)	*0.846 (0.809, 0.886)*	*0.652 (0.611, 0.696)*	*0.658 (0.621, 0.697)*
		20	*0.583 (0.551, 0.618)*	*0.918 (0.843, 0.999)*	*0.866 (0.830, 0.902)*	*0.628 (0.590, 0.668)*	*0.644 (0.610, 0.681)*
Setup C	800	10	*0.357 (0.334, 0.381)*	0.985 (0.901, 1.076)	*0.561 (0.519, 0.607)*	*0.588 (0.534, 0.647)*	*0.640 (0.582, 0.705)*
		20	*0.366 (0.342, 0.392)*	0.932 (0.836, 1.039)	*0.464 (0.416, 0.517)*	*0.582 (0.517, 0.655)*	*0.657 (0.585, 0.737)*
	1600	10	*0.344 (0.315, 0.377)*	*0.612 (0.547, 0.685)*	*0.511 (0.467, 0.560)*	*0.506 (0.447, 0.573)*	*0.579 (0.511, 0.655)*
		20	*0.360 (0.330, 0.393)*	*0.642 (0.559, 0.738)*	*0.434 (0.384, 0.492)*	*0.436 (0.375, 0.508)*	*0.508 (0.436, 0.592)*
Setup D	800	10	*0.870 (0.856, 0.884)*	**1.134 (1.080, 1.190)**	**1.029 (1.007, 1.052)**	**1.083 (1.058, 1.108)**	**1.111 (1.087, 1.135)**
		20	*0.902 (0.889, 0.916)*	**1.142 (1.093, 1.194)**	**1.086 (1.065, 1.107)**	**1.102 (1.079, 1.124)**	**1.123 (1.102, 1.144)**
	1600	10	*0.845 (0.827, 0.864)*	**1.090 (1.025, 1.160)**	1.025 (0.997, 1.054)	1.007 (0.977, 1.037)	**1.052 (1.024, 1.081)**
		20	*0.863 (0.846, 0.881)*	**1.153 (1.091, 1.218)**	**1.049 (1.023, 1.075)**	**1.064 (1.037, 1.092)**	**1.101 (1.076, 1.127)**

Table S. 5: Results of **RQ 2** for the experimental setups in Section 4. Comparison of on the relative efficiency for $\hat{\tau}(\boldsymbol{x})$ in the different scenarios. Estimates and simultaneous 95 % confidence intervals were obtained from a normal linear mixed model with log-link. Cells printed in bold font correspond to a superior reference of the *Naive* model-based forests, and cells printed in italics indicate an inferior reference.

			Mean squared error ratio for **RQ 2**: Robinson$_{\hat{W}}$ vs. Naive				
DGP	N	P	Normal	Binomial	Multinomial	Weibull	Cox
Setup A	800	10	0.949 (0.843, 1.068)	*0.801 (0.732, 0.877)*	**1.289 (1.179, 1.409)**	*0.847 (0.752, 0.954)*	*0.796 (0.708, 0.894)*
		20	0.965 (0.841, 1.106)	*0.746 (0.663, 0.839)*	**1.416 (1.239, 1.618)**	*0.811 (0.708, 0.929)*	*0.759 (0.665, 0.867)*
	1600	10	1.081 (0.937, 1.248)	*0.886 (0.788, 0.996)*	**1.397 (1.246, 1.566)**	1.019 (0.883, 1.176)	0.987 (0.858, 1.136)
		20	1.110 (0.937, 1.314)	0.855 (0.728, 1.004)	**1.331 (1.131, 1.567)**	0.992 (0.827, 1.190)	0.953 (0.800, 1.137)
Setup A'	800	10	0.959 (0.856, 1.075)	*0.783 (0.718, 0.855)*	**1.295 (1.182, 1.418)**	*0.852 (0.759, 0.957)*	*0.812 (0.726, 0.909)*
		20	0.957 (0.835, 1.097)	*0.744 (0.664, 0.832)*	**1.374 (1.208, 1.563)**	*0.856 (0.748, 0.981)*	*0.818 (0.718, 0.931)*
	1600	10	1.075 (0.940, 1.230)	0.917 (0.818, 1.028)	**1.379 (1.229, 1.548)**	0.975 (0.851, 1.117)	0.938 (0.819, 1.073)
		20	1.043 (0.878, 1.239)	*0.836 (0.718, 0.974)*	**1.390 (1.188, 1.627)**	0.949 (0.793, 1.135)	0.900 (0.756, 1.072)
Setup B	800	10	**1.567 (1.500, 1.636)**	0.972 (0.907, 1.043)	**1.155 (1.115, 1.196)**	**1.455 (1.385, 1.529)**	**1.412 (1.352, 1.475)**
		20	**1.530 (1.472, 1.591)**	1.017 (0.951, 1.086)	**1.104 (1.069, 1.139)**	**1.410 (1.351, 1.472)**	**1.359 (1.308, 1.412)**
	1600	10	**1.672 (1.575, 1.776)**	1.021 (0.939, 1.110)	**1.182 (1.129, 1.237)**	**1.535 (1.438, 1.638)**	**1.519 (1.434, 1.610)**
		20	**1.714 (1.618, 1.817)**	**1.089 (1.001, 1.185)**	**1.155 (1.108, 1.205)**	**1.593 (1.496, 1.696)**	**1.552 (1.469, 1.640)**
Setup C	800	10	**1.295 (1.199, 1.399)**	**1.391 (1.284, 1.505)**	**1.476 (1.359, 1.604)**	**1.326 (1.195, 1.470)**	**1.227 (1.105, 1.363)**
		20	**1.451 (1.343, 1.567)**	1.039 (0.930, 1.160)	**1.545 (1.375, 1.738)**	**1.342 (1.181, 1.524)**	**1.220 (1.078, 1.382)**
	1600	10	**1.380 (1.242, 1.534)**	**2.887 (2.606, 3.199)**	**2.062 (1.882, 2.258)**	**1.802 (1.586, 2.046)**	**1.502 (1.319, 1.710)**
		20	**1.377 (1.245, 1.524)**	**1.920 (1.681, 2.193)**	**1.865 (1.639, 2.122)**	**1.849 (1.577, 2.168)**	**1.569 (1.336, 1.842)**
Setup D	800	10	**1.120 (1.102, 1.139)**	*0.908 (0.866, 0.952)*	0.985 (0.964, 1.006)	*0.906 (0.885, 0.928)*	*0.885 (0.866, 0.904)*
		20	**1.083 (1.066, 1.100)**	*0.897 (0.858, 0.936)*	*0.931 (0.914, 0.949)*	*0.893 (0.875, 0.912)*	*0.878 (0.862, 0.895)*
	1600	10	**1.135 (1.110, 1.160)**	0.952 (0.896, 1.012)	0.993 (0.966, 1.021)	*0.964 (0.935, 0.993)*	*0.928 (0.903, 0.954)*
		20	**1.115 (1.092, 1.138)**	*0.888 (0.841, 0.937)*	*0.967 (0.943, 0.990)*	*0.914 (0.890, 0.939)*	*0.889 (0.868, 0.910)*

Table S. 6: Results of **RQ 3** for the experimental setups in Section 4. Comparison on the relative efficiency for $\hat{\tau}(\boldsymbol{x})$ in the different scenarios. Estimates and simultaneous 95 % confidence intervals were obtained from a normal linear mixed model with log-link. Cells printed in bold font correspond to a superior reference of $Robinson_{\hat{W}}$, and cells printed in italics indicate an inferior reference.

			Mean squared error ratio for **RQ 3**: Robinson vs. Robinson$_{\hat{W}}$				
DGP	N	P	Normal	Binomial	Multinomial	Weibull	Cox
Setup A	800	10	1.053 (0.936, 1.186)	**1.249 (1.140, 1.367)**	*0.776 (0.710, 0.848)*	**1.181 (1.048, 1.330)**	**1.256 (1.118, 1.412)**
		20	1.037 (0.904, 1.189)	**1.341 (1.191, 1.509)**	*0.706 (0.618, 0.807)*	**1.233 (1.076, 1.413)**	**1.317 (1.154, 1.503)**
	1600	10	0.925 (0.801, 1.067)	**1.129 (1.004, 1.269)**	*0.716 (0.639, 0.802)*	0.981 (0.851, 1.132)	1.013 (0.881, 1.166)
		20	0.901 (0.761, 1.067)	1.170 (0.996, 1.374)	*0.751 (0.638, 0.884)*	1.008 (0.840, 1.210)	1.049 (0.879, 1.251)
Setup A'	800	10	1.042 (0.930, 1.168)	**1.276 (1.169, 1.394)**	*0.772 (0.705, 0.846)*	**1.174 (1.045, 1.318)**	**1.231 (1.100, 1.378)**
		20	1.045 (0.911, 1.198)	**1.345 (1.202, 1.505)**	*0.728 (0.640, 0.828)*	**1.168 (1.020, 1.337)**	**1.223 (1.074, 1.392)**
	1600	10	0.930 (0.813, 1.064)	1.091 (0.973, 1.223)	*0.725 (0.646, 0.814)*	1.026 (0.895, 1.175)	1.067 (0.932, 1.220)
		20	0.959 (0.807, 1.139)	**1.196 (1.027, 1.392)**	*0.719 (0.615, 0.842)*	1.054 (0.881, 1.261)	1.111 (0.933, 1.322)
Setup B	800	10	*0.638 (0.611, 0.667)*	1.028 (0.959, 1.103)	*0.866 (0.836, 0.897)*	*0.687 (0.654, 0.722)*	*0.708 (0.678, 0.740)*
		20	*0.653 (0.629, 0.679)*	0.984 (0.921, 1.051)	*0.906 (0.878, 0.935)*	*0.709 (0.679, 0.740)*	*0.736 (0.708, 0.764)*
	1600	10	*0.598 (0.563, 0.635)*	0.979 (0.901, 1.065)	*0.846 (0.809, 0.886)*	*0.652 (0.611, 0.695)*	*0.658 (0.621, 0.698)*
		20	*0.583 (0.550, 0.618)*	*0.918 (0.844, 0.999)*	*0.865 (0.830, 0.902)*	*0.628 (0.590, 0.668)*	*0.644 (0.610, 0.681)*
Setup C	800	10	*0.772 (0.715, 0.834)*	*0.719 (0.664, 0.779)*	*0.677 (0.624, 0.736)*	*0.754 (0.680, 0.837)*	*0.815 (0.734, 0.905)*
		20	*0.689 (0.638, 0.745)*	0.963 (0.862, 1.075)	*0.647 (0.576, 0.727)*	*0.745 (0.656, 0.847)*	*0.819 (0.724, 0.928)*
	1600	10	*0.724 (0.652, 0.805)*	*0.346 (0.313, 0.384)*	*0.485 (0.443, 0.531)*	*0.555 (0.489, 0.630)*	*0.666 (0.585, 0.758)*
		20	*0.726 (0.656, 0.803)*	*0.521 (0.456, 0.595)*	*0.536 (0.471, 0.610)*	*0.541 (0.461, 0.634)*	*0.637 (0.543, 0.749)*
Setup D	800	10	*0.893 (0.878, 0.908)*	**1.101 (1.050, 1.155)**	1.015 (0.994, 1.037)	**1.104 (1.078, 1.130)**	**1.130 (1.106, 1.154)**
		20	*0.923 (0.909, 0.938)*	**1.115 (1.068, 1.165)**	**1.074 (1.054, 1.094)**	**1.119 (1.097, 1.143)**	**1.139 (1.118, 1.161)**
	1600	10	*0.881 (0.862, 0.901)*	1.050 (0.988, 1.116)	1.007 (0.980, 1.035)	**1.038 (1.007, 1.070)**	**1.078 (1.049, 1.108)**
		20	*0.897 (0.879, 0.916)*	**1.126 (1.067, 1.189)**	**1.035 (1.010, 1.060)**	**1.094 (1.065, 1.123)**	**1.125 (1.098, 1.152)**

B. Empirical evaluation based on Wager and Athey (2018)

We evaluated the performance of our proposed model-based forest versions also with the study setting of Wager and Athey (2018), which were later reused by Athey, Tibshirani, and Wager (2019). Given uniformly distributed covariates $\boldsymbol{X} \sim U([0,1]^P)$ of dimensionality $P \in \{10, 20\}$ and a binomially distributed treatment indicator $W \mid \boldsymbol{X} = \boldsymbol{x} \sim \mathrm{B}(1, \pi(\boldsymbol{x}))$, the propensity function $\pi(\cdot)$ either did or did not depend on $\boldsymbol{x}$

$$\pi(\boldsymbol{x}) = \begin{cases} \pi \equiv 0.5 \\ \pi(x_1) = 1/4(1 + \beta_{2,4}(x_1)) \\ \pi(x_3) = 1/4(1 + \beta_{2,4}(x_3)) \\ \pi(x_4) = 1/4(1 + \beta_{2,4}(x_4)) \end{cases}$$

where $\beta_{2,4}$ is the β-density with shape 2 and scale 4. The probability $\pi \equiv 0.5$ indicates no confounding and thus a randomized trial. The treatment effect function $\tau(\cdot)$ was either 0 (no treatment effect) or depended on a smooth interaction function of x_1 and x_2

$$\tau(\boldsymbol{x}) = \begin{cases} \tau \equiv 0 \\ \tau(x_1, x_2) = \prod_{p=1,2} \left(1 + (1 + \exp(-20\,(x_p - 1/3)))^{-1}\right). \end{cases}$$

The prognostic effect function $\mu(\cdot)$ was either 0 (no prognostic effect) or linear in x_1 or x_3

$$\mu(\boldsymbol{x}) = \begin{cases} \mu \equiv 0 \\ \mu(x_1) = 2x_1 - 1 \\ \mu(x_3) = 2x_3 - 1. \end{cases}$$

We studied four different simulation models

$$(Y \mid \boldsymbol{X} = \boldsymbol{x}, W = w) \sim \begin{cases} \mathrm{N}(\mu(\boldsymbol{x}) + \tau(\boldsymbol{x})w, 1) & \text{(5a)} \\ \mathrm{B}(1, \mathrm{expit}(\mu(\boldsymbol{x}) + \tau(\boldsymbol{x})w)) & \text{(5b)} \\ \mathrm{M} \text{ with } \log(O(y_k \mid \boldsymbol{x}, w)) = \vartheta_k - \mu(\boldsymbol{x}) - \tau(\boldsymbol{x})w & \text{(5c)} \\ \mathrm{W} \text{ with } \log(H(y \mid \boldsymbol{x}, w)) = 2\log(y) - \mu(\boldsymbol{x}) - \tau(\boldsymbol{x})w & \text{(5d)} \end{cases}$$

Model (5a) is a normal linear regression model, model (5b) a binary logistic regression model, model (5c) is a 4-nomial model with log-odds function $\vartheta_k - \mu(\boldsymbol{x}) - \tau(\boldsymbol{x})w$ with threshold parameters $\vartheta_k = \mathrm{logit}(k/4)$ for $k = 1, 2, 3$, and model (5d) is a Weibull model with log-cumulative hazard function $2\log(y) - \mu(\boldsymbol{x}) - \tau(\boldsymbol{x})w$. We added 50 % random right-censoring to the Weibull-generated data and also applied a Cox proportional hazards model in addition to the Weibull model.

For the additive predictor $\mu(\boldsymbol{x}) + \tau(\boldsymbol{x})w$ we considered the 16 scenarios as specified in Table S. 7. Compared to Part A of this table, in Part B half of the (negative) predictive effect is added to the prognostic effect. We term the implied scenario where at least one variable exists which is both prognostic (impact in $\mu(\boldsymbol{x})$) and predictive (impact in $\tau(\boldsymbol{x})$) as overlay. $W(x_1)$, $W(x_3)$ and $W(x_4)$ depict that W was drawn from a Bernoulli distribution with $\pi(x_1)$, $\pi(x_3)$ or $\pi(x_4)$, respectively.

In Part A of Table S. 7, the prognostic term and the predictive term are separate and there is only overlay of prognostic and predictive effects when both terms depend on x_1, *i.e.* x_1 is both prognostic and predictive in this scenario. The treatment assignment probability may

Table S. 7: Experimental setup B. Confounding is present for non-constant propensities $\pi(\boldsymbol{x})$, an instrumental variable impacts $\pi(\boldsymbol{x})$ exclusively, heterogeneity of the treatment effect $\tau(\boldsymbol{x})$ is present when τ is non-constant, and overlay refers to variables being prognostic (impact in $\mu(\boldsymbol{x})$) and predictive (impact in $\tau(\boldsymbol{x})$) at the same time.

	Additive Predictor	Confounding	Instrument	Heterogeneity	Overlay
Part A	$\mu(x_3) + 0 \cdot W(x_3)$	yes	no	no	no
	$\tau(x_1, x_2)W$	no	no	yes	no
	$\mu(x_1) + \tau(x_1, x_2)W(x_1)$	yes	no	yes	yes
	$\mu(x_1) + \tau(x_1, x_2)W$	no	no	yes	yes
	$\mu(x_3) + \tau(x_1, x_2)W$	no	no	yes	no
	$\mu(x_3) + \tau(x_1, x_2)W(x_3)$	yes	no	yes	no
	$\tau(x_1, x_2)W(x_3)$	no	yes	yes	no
	$\mu(x_3) + \tau(x_1, x_2)W(x_4)$	no	yes	yes	no
Part B	$\mu(x_3) + 0 \cdot (W(x_3) - 0.5)$	yes	no	no	no
	$\tau(x_1, x_2)(W - 0.5)$	no	no	yes	yes
	$\mu(x_1) + \tau(x_1, x_2)(W(x_1) - 0.5)$	yes	no	yes	yes
	$\mu(x_1) + \tau(x_1, x_2)(W - 0.5)$	no	no	yes	yes
	$\mu(x_3) + \tau(x_1, x_2)(W - 0.5)$	no	no	yes	yes
	$\mu(x_3) + \tau(x_1, x_2)(W(x_3) - 0.5)$	yes	no	yes	yes
	$\tau(x_1, x_2)(W(x_3) - 0.5)$	no	yes	yes	yes
	$\mu(x_3) + \tau(x_1, x_2)(W(x_4) - 0.5)$	no	yes	yes	yes

depend on x_1, x_3, or x_4. In the third scenario, x_1 is a predictive confounder (with impact on μ, τ, and π) and in the last two scenarios, x_3 and x_4 can be understood as instruments with direct impact on treatment assignment but without direct impact on the response. In Part B of this table, half of the predictive effect is added to the prognostic effect, so there is always overlay of both types of effects.

Again, we used random forests to estimate $\pi(\boldsymbol{x})$ and gradient boosting machines to estimate $\eta_0(\boldsymbol{x})$ and $\eta_1(\boldsymbol{x})$ as described in Section 4. We also applied the same performance assessment (mean squared error evaluated on 1000 test samples). The results are presented in Figures S. 4 and S. 5. The results for the statistical analysis of RQ 1 to RQ 3 based on a normal linear mixed model are presented in Table S. 8 to S. 10.

Results

For the normal distribution (first row of Figures S. 4 and S. 5), model-based forests with centered W (*Robinson*$_{\hat{W}}$) performed better than naive model-based forests without centering in case of confounding (columns 1 and 6). If predictive covariates were also prognostic (column 3), the effect of local centering on performance diminished. In case of variables that only influence the treatment assignment but not the outcome (column 7 and 8), solely centering W led to biased results. Especially in this scenario, additional adding $\hat{m}(\boldsymbol{x})$ as an offset (*Robinson*) is recommended. However, also in all other scenarios *Robinson* model-based forests perform at least as well as *Robinson*$_{\hat{W}}$ forests – except for the setup without a prognostic effect ($\mu(\boldsymbol{x}) \equiv 0$, column 2, see also Table reftab:lmeradaptive3).

We obtained similar results for the other distributions as shown in Figures S. 4 and S. 5.

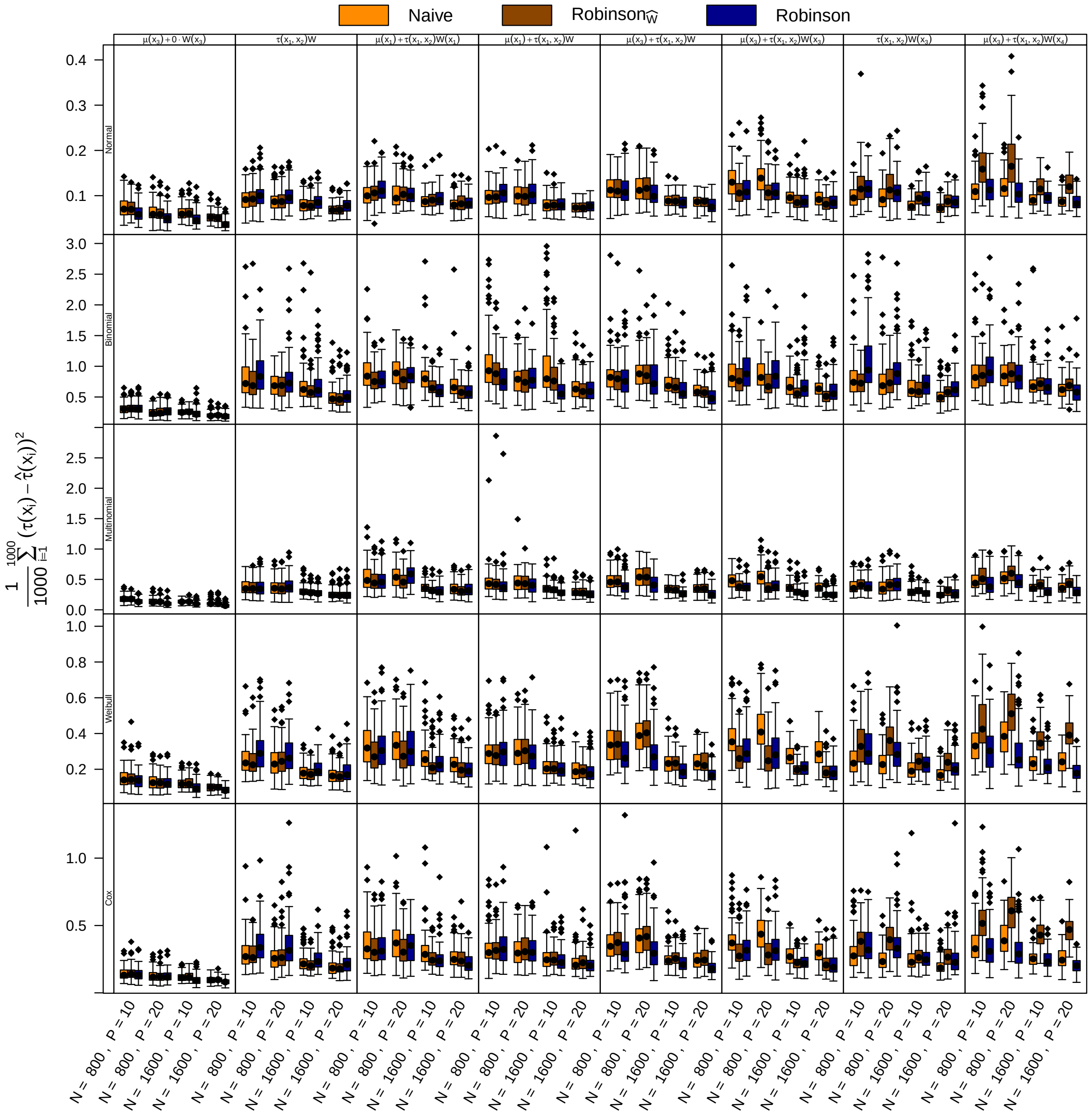

Figure S. 4: Model-based forest results for Part A (Table S. 7), Cox means a Cox model applied to the Weibull data. For the Weibull and Cox model, treatment effects $\tau(\boldsymbol{x})$ are estimated as conditional log hazard ratios. Direct comparison of model-based forests without centering (Naive), model-based forests with local centering according to Robinson (1988) of Y and W (*Robinson*) or only of W (*Robinson*$_{\widehat{W}}$).

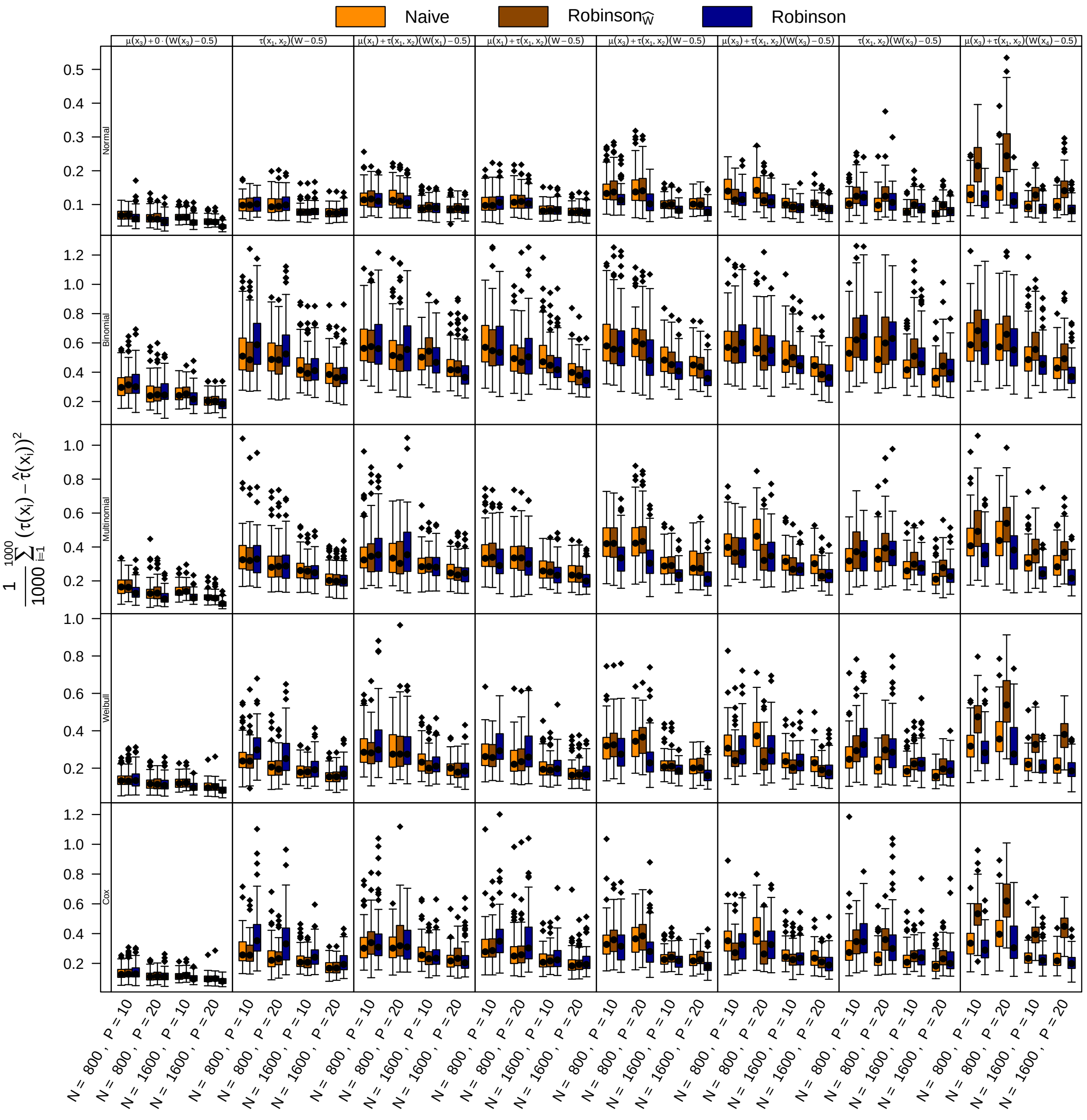

Figure S. 5: Model-based forest results for Part B (Table S. 7), Cox means a Cox model applied to the Weibull data. For the Weibull and Cox model, treatment effects $\tau(\boldsymbol{x})$ are estimated as conditional log hazard ratios. Direct comparison of model-based forests without centering (Naive), model-based forests with local centering according to Robinson (1988) of Y and W (*Robinson*) or only of W (*Robinson*$_{\widehat{W}}$).

Table S. 8: Results of **RQ 1** for the experimental setups in Section B. Comparison of mean squared errors for $\hat{\tau}(\boldsymbol{x})$ in the different scenarios. Estimates and simultaneous 95 % confidence intervals were obtained from a normal linear mixed model with log-link. Cells printed in bold font correspond to a superior reference of the naive model-based forests, cells printed in italics indicate an inferior reference of naive model-based forests.

				Mean squared error ratio for RQ 1: Robinson vs. Naive				
Part	DGP	N	P	Normal	Binomial	Multinomial	Weibull	Cox
A	$\mu(x_3) + 0 \cdot W(x_3)$	800	10	*0.837 (0.762, 0.919)*	1.025 (0.713, 1.474)	*0.766 (0.660, 0.888)*	0.927 (0.769, 1.118)	0.975 (0.759, 1.254)
			20	*0.808 (0.723, 0.903)*	1.123 (0.726, 1.735)	*0.761 (0.624, 0.928)*	0.931 (0.755, 1.149)	0.975 (0.738, 1.287)
		1600	10	*0.795 (0.707, 0.894)*	0.912 (0.570, 1.462)	*0.742 (0.601, 0.915)*	0.821 (0.641, 1.053)	0.848 (0.609, 1.180)
			20	*0.697 (0.601, 0.809)*	0.942 (0.526, 1.686)	*0.696 (0.533, 0.907)*	0.829 (0.613, 1.123)	0.852 (0.569, 1.276)
	$\tau(x_1, x_2)W$	800	10	**1.106 (1.036, 1.180)**	**3.295 (2.924, 3.714)**	0.976 (0.918, 1.037)	**1.222 (1.111, 1.344)**	**1.220 (1.098, 1.356)**
			20	**1.094 (1.023, 1.171)**	**1.210 (1.044, 1.402)**	1.047 (0.986, 1.112)	**1.127 (1.019, 1.248)**	**1.379 (1.234, 1.541)**
		1600	10	**1.085 (1.005, 1.171)**	1.012 (0.878, 1.167)	*0.897 (0.827, 0.972)*	1.080 (0.938, 1.245)	1.093 (0.946, 1.262)
			20	**1.128 (1.034, 1.231)**	1.021 (0.826, 1.261)	1.004 (0.918, 1.098)	1.108 (0.954, 1.287)	1.140 (0.969, 1.342)
	$\mu(x_1) + \tau(x_1, x_2)W(x_1)$	800	10	**1.118 (1.054, 1.185)**	*0.805 (0.706, 0.917)*	*0.924 (0.885, 0.966)*	0.970 (0.895, 1.052)	0.916 (0.833, 1.008)
			20	0.988 (0.929, 1.051)	0.943 (0.831, 1.070)	1.032 (0.990, 1.074)	*0.923 (0.853, 0.998)*	0.912 (0.831, 1.002)
		1600	10	**1.078 (1.005, 1.156)**	*0.188 (0.157, 0.225)*	*0.872 (0.814, 0.935)*	*0.875 (0.787, 0.973)*	*0.805 (0.712, 0.910)*
			20	1.049 (0.970, 1.133)	*0.633 (0.532, 0.753)*	0.981 (0.919, 1.048)	*0.845 (0.747, 0.956)*	*0.857 (0.742, 0.990)*
	$\mu(x_1) + \tau(x_1, x_2)W$	800	10	**1.076 (1.011, 1.146)**	*0.465 (0.408, 0.531)*	0.964 (0.917, 1.013)	1.013 (0.927, 1.106)	1.059 (0.956, 1.173)
			20	1.063 (1.000, 1.130)	0.991 (0.862, 1.139)	*0.877 (0.831, 0.926)*	*0.911 (0.832, 0.997)*	0.990 (0.889, 1.104)
		1600	10	1.025 (0.947, 1.109)	*0.307 (0.259, 0.363)*	*0.795 (0.738, 0.857)*	0.946 (0.834, 1.072)	0.901 (0.788, 1.031)
			20	1.027 (0.943, 1.119)	0.918 (0.769, 1.096)	*0.880 (0.809, 0.958)*	0.933 (0.812, 1.072)	0.863 (0.740, 1.007)
	$\mu(x_3) + \tau(x_1, x_2)W$	800	10	0.984 (0.931, 1.041)	**1.166 (1.032, 1.318)**	*0.802 (0.760, 0.846)*	*0.821 (0.753, 0.894)*	0.951 (0.860, 1.052)
			20	*0.875 (0.827, 0.926)*	0.897 (0.789, 1.020)	*0.800 (0.763, 0.839)*	*0.690 (0.635, 0.751)*	*0.724 (0.654, 0.801)*
		1600	10	0.951 (0.883, 1.023)	0.946 (0.807, 1.110)	*0.776 (0.716, 0.840)*	*0.820 (0.723, 0.930)*	0.880 (0.759, 1.019)
			20	*0.902 (0.834, 0.976)*	0.848 (0.693, 1.038)	*0.741 (0.685, 0.802)*	*0.688 (0.598, 0.790)*	*0.735 (0.622, 0.868)*
	$\mu(x_3) + \tau(x_1, x_2)W(x_3)$	800	10	*0.865 (0.821, 0.911)*	**1.161 (1.031, 1.308)**	*0.778 (0.736, 0.823)*	*0.820 (0.757, 0.888)*	*0.844 (0.765, 0.931)*
			20	*0.764 (0.726, 0.804)*	1.102 (0.974, 1.248)	*0.747 (0.711, 0.786)*	*0.717 (0.663, 0.774)*	*0.731 (0.666, 0.803)*
		1600	10	0.944 (0.882, 1.011)	0.867 (0.744, 1.011)	*0.744 (0.690, 0.803)*	*0.748 (0.666, 0.840)*	*0.804 (0.698, 0.926)*
			20	*0.918 (0.855, 0.986)*	0.982 (0.821, 1.174)	*0.697 (0.645, 0.754)*	*0.632 (0.559, 0.715)*	*0.701 (0.608, 0.809)*
	$\tau(x_1, x_2)W(x_3)$	800	10	**1.213 (1.142, 1.289)**	**1.537 (1.372, 1.722)**	1.013 (0.954, 1.076)	**1.265 (1.154, 1.386)**	**1.221 (1.096, 1.360)**
			20	**1.148 (1.080, 1.220)**	**1.349 (1.189, 1.530)**	**1.179 (1.112, 1.250)**	**1.335 (1.207, 1.477)**	**1.468 (1.301, 1.657)**
		1600	10	**1.227 (1.138, 1.323)**	**1.178 (1.011, 1.372)**	*0.916 (0.844, 0.993)*	**1.150 (1.015, 1.304)**	0.965 (0.843, 1.105)
			20	**1.200 (1.106, 1.301)**	**1.278 (1.052, 1.553)**	**1.152 (1.051, 1.262)**	**1.265 (1.094, 1.462)**	**1.366 (1.167, 1.599)**
	$\mu(x_3) + \tau(x_1, x_2)W(x_4)$	800	10	1.017 (0.961, 1.076)	**1.191 (1.063, 1.334)**	*0.877 (0.833, 0.924)*	*0.913 (0.843, 0.990)*	0.942 (0.854, 1.040)
			20	*0.907 (0.857, 0.960)*	0.999 (0.880, 1.135)	*0.901 (0.860, 0.943)*	*0.780 (0.720, 0.845)*	*0.810 (0.736, 0.891)*
		1600	10	1.048 (0.978, 1.123)	*0.841 (0.722, 0.980)*	*0.847 (0.788, 0.909)*	0.946 (0.841, 1.063)	0.932 (0.811, 1.071)
			20	0.967 (0.898, 1.042)	0.943 (0.785, 1.133)	*0.847 (0.790, 0.910)*	*0.752 (0.661, 0.857)*	*0.782 (0.669, 0.913)*
B	$\mu(x_3) + 0 \cdot (W(x_3) - 0.5)$	800	10	0.907 (0.820, 1.002)	1.033 (0.925, 1.154)	*0.820 (0.723, 0.930)*	1.028 (0.867, 1.219)	1.069 (0.850, 1.346)
			20	*0.818 (0.727, 0.921)*	1.047 (0.918, 1.194)	*0.739 (0.627, 0.872)*	0.980 (0.797, 1.205)	1.025 (0.773, 1.359)
		1600	10	*0.783 (0.693, 0.885)*	0.896 (0.772, 1.041)	*0.746 (0.634, 0.879)*	0.827 (0.662, 1.032)	0.869 (0.642, 1.177)
			20	*0.705 (0.597, 0.831)*	0.908 (0.755, 1.092)	*0.643 (0.503, 0.821)*	0.801 (0.610, 1.050)	0.833 (0.577, 1.204)
	$\tau(x_1, x_2)(W - 0.5)$	800	10	1.013 (0.948, 1.082)	**1.112 (1.050, 1.177)**	0.973 (0.922, 1.026)	**1.222 (1.123, 1.330)**	**1.414 (1.286, 1.555)**
			20	1.037 (0.971, 1.107)	**1.118 (1.047, 1.194)**	0.987 (0.928, 1.050)	**1.313 (1.189, 1.449)**	**1.440 (1.291, 1.606)**
		1600	10	1.039 (0.956, 1.129)	0.964 (0.890, 1.043)	0.964 (0.896, 1.036)	1.102 (0.974, 1.245)	**1.147 (1.004, 1.311)**
			20	1.033 (0.947, 1.127)	0.971 (0.888, 1.062)	0.983 (0.900, 1.074)	1.140 (0.987, 1.317)	**1.240 (1.047, 1.468)**
	$\mu(x_1) + \tau(x_1, x_2)(W(x_1) - 0.5)$	800	10	0.954 (0.901, 1.011)	**1.074 (1.016, 1.135)**	**1.082 (1.028, 1.139)**	**1.130 (1.048, 1.218)**	1.094 (0.999, 1.198)
			20	*0.931 (0.879, 0.986)*	**1.131 (1.063, 1.203)**	**1.140 (1.083, 1.201)**	1.032 (0.953, 1.118)	1.069 (0.970, 1.179)
		1600	10	1.017 (0.944, 1.096)	0.994 (0.926, 1.068)	0.987 (0.924, 1.054)	0.947 (0.854, 1.051)	0.944 (0.835, 1.067)
			20	0.974 (0.900, 1.053)	0.935 (0.858, 1.018)	0.977 (0.904, 1.057)	0.984 (0.870, 1.113)	1.029 (0.892, 1.187)
	$\mu(x_1) + \tau(x_1, x_2)(W - 0.5)$	800	10	1.024 (0.962, 1.089)	0.969 (0.916, 1.024)	*0.874 (0.827, 0.923)*	**1.152 (1.061, 1.250)**	**1.179 (1.078, 1.289)**
			20	*0.940 (0.884, 1.000)*	**1.071 (1.004, 1.143)**	*0.942 (0.889, 0.997)*	**1.192 (1.091, 1.303)**	**1.257 (1.138, 1.388)**
		1600	10	1.002 (0.925, 1.085)	*0.871 (0.809, 0.937)*	*0.902 (0.838, 0.971)*	1.028 (0.917, 1.153)	1.101 (0.963, 1.259)
			20	0.976 (0.895, 1.064)	*0.871 (0.794, 0.956)*	*0.867 (0.796, 0.944)*	1.001 (0.872, 1.149)	1.039 (0.892, 1.212)
	$\mu(x_3) + \tau(x_1, x_2)(W - 0.5)$	800	10	*0.821 (0.780, 0.865)*	0.957 (0.905, 1.012)	*0.792 (0.752, 0.833)*	*0.872 (0.805, 0.944)*	*0.903 (0.820, 0.994)*
			20	*0.705 (0.669, 0.744)*	*0.845 (0.793, 0.900)*	*0.728 (0.691, 0.766)*	*0.730 (0.671, 0.793)*	*0.775 (0.702, 0.856)*
		1600	10	*0.850 (0.790, 0.915)*	*0.840 (0.777, 0.909)*	*0.787 (0.732, 0.847)*	*0.863 (0.766, 0.974)*	0.935 (0.810, 1.081)
			20	*0.791 (0.734, 0.851)*	*0.805 (0.737, 0.878)*	*0.721 (0.668, 0.779)*	*0.780 (0.682, 0.892)*	*0.807 (0.687, 0.948)*
	$\mu(x_3) + \tau(x_1, x_2)(W(x_3) - 0.5)$	800	10	*0.810 (0.770, 0.853)*	**1.084 (1.022, 1.149)**	*0.927 (0.882, 0.973)*	0.979 (0.908, 1.056)	0.927 (0.844, 1.017)
			20	*0.720 (0.684, 0.759)*	*0.911 (0.857, 0.968)*	*0.757 (0.721, 0.794)*	*0.787 (0.733, 0.844)*	*0.783 (0.719, 0.853)*
		1600	10	*0.884 (0.825, 0.948)*	*0.909 (0.844, 0.978)*	*0.870 (0.814, 0.930)*	0.946 (0.855, 1.045)	0.945 (0.831, 1.075)
			20	*0.829 (0.772, 0.890)*	*0.847 (0.776, 0.925)*	*0.755 (0.701, 0.813)*	*0.830 (0.738, 0.934)*	*0.832 (0.718, 0.965)*
	$\tau(x_1, x_2)(W(x_3) - 0.5)$	800	10	**1.152 (1.085, 1.222)**	**1.277 (1.206, 1.353)**	**1.082 (1.023, 1.144)**	**1.300 (1.200, 1.409)**	**1.212 (1.101, 1.334)**
			20	**1.107 (1.040, 1.179)**	**1.228 (1.154, 1.306)**	**1.127 (1.069, 1.190)**	**1.470 (1.335, 1.618)**	**1.498 (1.335, 1.680)**
		1600	10	**1.132 (1.045, 1.226)**	**1.136 (1.054, 1.225)**	1.040 (0.970, 1.116)	**1.228 (1.095, 1.377)**	1.123 (0.980, 1.286)
			20	**1.126 (1.032, 1.227)**	**1.122 (1.026, 1.226)**	**1.096 (1.006, 1.194)**	**1.233 (1.077, 1.410)**	**1.262 (1.078, 1.477)**
	$\mu(x_3) + \tau(x_1, x_2)(W(x_4) - 0.5)$	800	10	*0.864 (0.819, 0.911)*	1.021 (0.966, 1.080)	*0.823 (0.784, 0.864)*	*0.907 (0.838, 0.982)*	*0.891 (0.806, 0.986)*
			20	*0.718 (0.682, 0.755)*	0.976 (0.921, 1.035)	*0.843 (0.806, 0.883)*	*0.863 (0.805, 0.925)*	*0.850 (0.780, 0.925)*
		1600	10	0.935 (0.869, 1.006)	*0.903 (0.841, 0.971)*	*0.790 (0.737, 0.846)*	0.935 (0.837, 1.045)	0.954 (0.833, 1.093)
			20	*0.861 (0.801, 0.925)*	*0.897 (0.822, 0.978)*	*0.762 (0.706, 0.821)*	0.899 (0.799, 1.011)	0.911 (0.786, 1.055)

Table S. 9: Results of **RQ 2** for the experimental setups in Section B. Comparison of mean squared errors for $\hat{\tau}(\boldsymbol{x})$ in the different scenarios. Estimates and simultaneous 95 % confidence intervals were obtained from a normal linear mixed model with log-link. Cells printed in bold font correspond to a superior reference of the naive model-based forests, cells printed in italics indicate an inferior reference of naive model-based forests.

			Mean squared error ratio for RQ 2: Robinson$_{\hat{W}}$ vs. Naive				
DGP	N	P	Normal	Binomial	Multinomial	Weibull	Cox
$\mu(x_3) + 0 \cdot W(x_3)$	800	10	**1.182 (1.076, 1.298)**	1.012 (0.709, 1.446)	**1.284 (1.106, 1.491)**	1.109 (0.922, 1.335)	1.063 (0.831, 1.361)
		20	**1.186 (1.059, 1.328)**	0.917 (0.598, 1.408)	**1.304 (1.069, 1.591)**	1.018 (0.821, 1.263)	0.987 (0.744, 1.310)
	1600	10	**1.277 (1.137, 1.436)**	1.131 (0.711, 1.800)	**1.375 (1.116, 1.693)**	1.238 (0.967, 1.584)	1.224 (0.883, 1.695)
		20	**1.392 (1.197, 1.618)**	1.084 (0.608, 1.930)	**1.430 (1.096, 1.866)**	1.203 (0.889, 1.629)	1.200 (0.804, 1.790)
$\tau(x_1, x_2)W$	800	10	*0.913 (0.856, 0.974)*	*0.537 (0.499, 0.577)*	0.998 (0.939, 1.061)	*0.785 (0.712, 0.866)*	*0.787 (0.706, 0.876)*
		20	*0.913 (0.853, 0.976)*	*0.808 (0.695, 0.938)*	0.954 (0.898, 1.013)	0.906 (0.820, 1.002)	*0.766 (0.688, 0.854)*
	1600	10	*0.920 (0.853, 0.993)*	*0.822 (0.703, 0.961)*	1.060 (0.976, 1.151)	0.879 (0.760, 1.017)	*0.816 (0.700, 0.952)*
		20	*0.888 (0.814, 0.969)*	0.938 (0.756, 1.164)	0.989 (0.904, 1.081)	0.877 (0.753, 1.022)	*0.826 (0.697, 0.978)*
$\mu(x_1) + \tau(x_1, x_2)W(x_1)$	800	10	0.948 (0.896, 1.004)	1.000 (0.867, 1.153)	0.959 (0.916, 1.005)	*0.898 (0.824, 0.979)*	0.986 (0.892, 1.089)
		20	1.038 (0.977, 1.103)	0.941 (0.823, 1.076)	*0.817 (0.781, 0.854)*	*0.918 (0.843, 0.999)*	0.941 (0.851, 1.040)
	1600	10	0.951 (0.887, 1.018)	1.006 (0.785, 1.289)	1.026 (0.954, 1.103)	0.911 (0.810, 1.025)	0.972 (0.848, 1.114)
		20	1.001 (0.928, 1.080)	0.959 (0.782, 1.175)	*0.924 (0.862, 0.991)*	1.020 (0.894, 1.164)	1.148 (0.993, 1.327)
$\mu(x_1) + \tau(x_1, x_2)W$	800	10	*0.938 (0.881, 0.998)*	**1.826 (1.593, 2.093)**	**1.107 (1.055, 1.161)**	0.964 (0.881, 1.054)	0.932 (0.841, 1.033)
		20	0.945 (0.889, 1.004)	0.984 (0.854, 1.133)	**1.096 (1.037, 1.158)**	**1.108 (1.013, 1.213)**	1.052 (0.946, 1.170)
	1600	10	0.978 (0.904, 1.058)	**2.057 (1.722, 2.456)**	**1.196 (1.108, 1.291)**	1.036 (0.912, 1.177)	1.051 (0.916, 1.205)
		20	0.975 (0.895, 1.063)	1.018 (0.848, 1.221)	**1.134 (1.042, 1.234)**	1.058 (0.919, 1.217)	1.115 (0.955, 1.303)
$\mu(x_3) + \tau(x_1, x_2)W$	800	10	1.018 (0.963, 1.076)	*0.823 (0.727, 0.933)*	**1.257 (1.191, 1.326)**	**1.231 (1.130, 1.340)**	**1.107 (1.003, 1.221)**
		20	**1.147 (1.084, 1.213)**	1.064 (0.933, 1.214)	**1.267 (1.208, 1.329)**	**1.473 (1.356, 1.601)**	**1.435 (1.298, 1.586)**
	1600	10	1.049 (0.975, 1.130)	1.014 (0.863, 1.192)	**1.276 (1.179, 1.382)**	**1.217 (1.072, 1.380)**	**1.172 (1.013, 1.355)**
		20	**1.108 (1.024, 1.199)**	1.138 (0.927, 1.397)	**1.358 (1.256, 1.469)**	**1.479 (1.288, 1.698)**	**1.434 (1.217, 1.690)**
$\mu(x_3) + \tau(x_1, x_2)W(x_3)$	800	10	0.971 (0.918, 1.027)	*0.794 (0.702, 0.898)*	1.061 (0.998, 1.127)	*0.894 (0.815, 0.980)*	0.938 (0.841, 1.045)
		20	0.980 (0.925, 1.038)	*0.777 (0.679, 0.889)*	0.946 (0.893, 1.003)	*0.877 (0.798, 0.964)*	0.904 (0.809, 1.011)
	1600	10	0.989 (0.922, 1.061)	*0.819 (0.686, 0.977)*	**1.127 (1.039, 1.222)**	0.961 (0.841, 1.099)	0.996 (0.852, 1.164)
		20	0.972 (0.902, 1.049)	0.843 (0.692, 1.026)	1.002 (0.916, 1.096)	1.021 (0.884, 1.181)	1.006 (0.854, 1.186)
$\tau(x_1, x_2)W(x_3)$	800	10	1.043 (0.988, 1.100)	*0.570 (0.502, 0.646)*	**1.074 (1.014, 1.138)**	1.027 (0.948, 1.112)	1.055 (0.961, 1.159)
		20	**1.103 (1.045, 1.165)**	*0.721 (0.634, 0.820)*	0.982 (0.930, 1.036)	**1.155 (1.065, 1.251)**	**1.116 (1.018, 1.223)**
	1600	10	1.014 (0.948, 1.084)	*0.774 (0.660, 0.910)*	**1.140 (1.053, 1.235)**	1.072 (0.958, 1.200)	1.056 (0.925, 1.206)
		20	1.021 (0.949, 1.098)	0.883 (0.736, 1.058)	**1.151 (1.063, 1.246)**	**1.158 (1.028, 1.305)**	1.028 (0.901, 1.173)
$\mu(x_3) + \tau(x_1, x_2)W(x_4)$	800	10	**1.490 (1.420, 1.563)**	*0.814 (0.725, 0.914)*	**1.295 (1.233, 1.360)**	**1.476 (1.374, 1.586)**	**1.597 (1.466, 1.738)**
		20	**1.706 (1.625, 1.792)**	1.019 (0.898, 1.156)	**1.272 (1.219, 1.329)**	**1.701 (1.581, 1.831)**	**1.810 (1.661, 1.972)**
	1600	10	**1.268 (1.193, 1.348)**	1.026 (0.873, 1.205)	**1.381 (1.292, 1.477)**	**1.604 (1.451, 1.774)**	**1.782 (1.585, 2.003)**
		20	**1.467 (1.375, 1.565)**	1.154 (0.967, 1.377)	**1.427 (1.336, 1.524)**	**2.160 (1.927, 2.422)**	**2.323 (2.033, 2.653)**
$\mu(x_3) + 0 \cdot (W(x_3) - 0.5)$	800	10	1.100 (0.995, 1.215)	1.000 (0.897, 1.115)	**1.211 (1.068, 1.374)**	1.007 (0.852, 1.190)	0.985 (0.787, 1.233)
		20	**1.194 (1.059, 1.345)**	0.956 (0.839, 1.091)	**1.352 (1.147, 1.594)**	1.017 (0.827, 1.250)	0.987 (0.746, 1.306)
	1600	10	**1.309 (1.160, 1.478)**	**1.161 (1.003, 1.345)**	**1.372 (1.167, 1.613)**	1.232 (0.989, 1.535)	1.203 (0.893, 1.619)
		20	**1.395 (1.181, 1.647)**	1.119 (0.932, 1.344)	**1.526 (1.193, 1.951)**	1.245 (0.949, 1.634)	1.227 (0.852, 1.768)
$\tau(x_1, x_2)(W - 0.5)$	800	10	0.994 (0.931, 1.061)	*0.843 (0.794, 0.895)*	0.999 (0.946, 1.055)	*0.791 (0.725, 0.862)*	*0.682 (0.618, 0.752)*
		20	0.975 (0.913, 1.040)	*0.871 (0.814, 0.931)*	1.017 (0.956, 1.082)	*0.746 (0.675, 0.825)*	*0.668 (0.597, 0.747)*
	1600	10	0.965 (0.888, 1.048)	0.969 (0.893, 1.052)	0.994 (0.924, 1.071)	0.894 (0.790, 1.012)	*0.826 (0.720, 0.949)*
		20	0.974 (0.893, 1.062)	0.966 (0.881, 1.060)	0.997 (0.912, 1.090)	0.868 (0.751, 1.004)	*0.813 (0.688, 0.962)*
$\mu(x_1) + \tau(x_1, x_2)(W(x_1) - 0.5)$	800	10	1.054 (0.996, 1.116)	0.998 (0.946, 1.053)	0.953 (0.906, 1.002)	*0.853 (0.789, 0.921)*	0.947 (0.866, 1.036)
		20	1.035 (0.976, 1.097)	*0.881 (0.828, 0.937)*	*0.878 (0.834, 0.924)*	1.019 (0.943, 1.102)	1.091 (0.996, 1.196)
	1600	10	1.022 (0.950, 1.100)	**1.104 (1.031, 1.182)**	1.029 (0.964, 1.098)	0.933 (0.835, 1.042)	0.937 (0.822, 1.067)
		20	1.061 (0.983, 1.146)	**1.113 (1.024, 1.210)**	1.020 (0.944, 1.103)	0.960 (0.846, 1.090)	1.063 (0.927, 1.220)
$\mu(x_1) + \tau(x_1, x_2)(W - 0.5)$	800	10	0.986 (0.926, 1.048)	0.952 (0.899, 1.009)	**1.128 (1.068, 1.192)**	*0.849 (0.782, 0.923)*	*0.843 (0.770, 0.922)*
		20	**1.066 (1.003, 1.134)**	*0.875 (0.818, 0.935)*	1.057 (0.999, 1.120)	*0.856 (0.784, 0.935)*	*0.811 (0.735, 0.895)*
	1600	10	1.004 (0.928, 1.087)	**1.084 (1.005, 1.169)**	**1.078 (1.001, 1.162)**	0.934 (0.831, 1.051)	0.904 (0.791, 1.035)
		20	1.038 (0.952, 1.131)	1.091 (0.993, 1.200)	**1.144 (1.051, 1.246)**	0.993 (0.865, 1.141)	0.964 (0.827, 1.123)
$\mu(x_3) + \tau(x_1, x_2)(W - 0.5)$	800	10	**1.237 (1.175, 1.302)**	1.003 (0.948, 1.062)	**1.260 (1.197, 1.326)**	**1.175 (1.086, 1.271)**	**1.151 (1.047, 1.266)**
		20	**1.421 (1.348, 1.498)**	**1.167 (1.095, 1.243)**	**1.391 (1.321, 1.464)**	**1.414 (1.302, 1.535)**	**1.342 (1.217, 1.480)**
	1600	10	**1.192 (1.108, 1.283)**	**1.124 (1.036, 1.218)**	**1.250 (1.161, 1.345)**	**1.168 (1.036, 1.317)**	1.132 (0.983, 1.303)
		20	**1.261 (1.171, 1.358)**	**1.216 (1.113, 1.328)**	**1.393 (1.291, 1.504)**	**1.320 (1.156, 1.508)**	**1.319 (1.127, 1.543)**
$\mu(x_3) + \tau(x_1, x_2)(W(x_3) - 0.5)$	800	10	1.028 (0.973, 1.087)	0.967 (0.913, 1.024)	1.003 (0.954, 1.055)	*0.821 (0.755, 0.893)*	*0.857 (0.772, 0.951)*
		20	**1.067 (1.007, 1.131)**	0.982 (0.921, 1.047)	0.999 (0.947, 1.055)	*0.845 (0.776, 0.921)*	*0.859 (0.775, 0.952)*
	1600	10	1.027 (0.955, 1.105)	**1.129 (1.050, 1.214)**	1.044 (0.974, 1.118)	0.902 (0.809, 1.006)	0.973 (0.850, 1.113)
		20	1.074 (0.997, 1.157)	1.074 (0.981, 1.177)	1.024 (0.943, 1.111)	0.994 (0.874, 1.129)	1.038 (0.886, 1.215)
$\tau(x_1, x_2)(W(x_3) - 0.5)$	800	10	**1.078 (1.022, 1.137)**	1.007 (0.957, 1.058)	**1.115 (1.060, 1.174)**	*0.914 (0.850, 0.982)*	0.925 (0.846, 1.011)
		20	**1.204 (1.140, 1.271)**	1.001 (0.947, 1.058)	**1.088 (1.037, 1.142)**	0.987 (0.913, 1.066)	1.006 (0.919, 1.101)
	1600	10	**1.152 (1.075, 1.236)**	**1.111 (1.039, 1.188)**	**1.140 (1.068, 1.216)**	0.983 (0.886, 1.089)	1.024 (0.903, 1.161)
		20	**1.192 (1.106, 1.286)**	**1.116 (1.031, 1.208)**	**1.192 (1.105, 1.285)**	1.044 (0.928, 1.174)	1.063 (0.929, 1.216)
$\mu(x_3) + \tau(x_1, x_2)(W(x_4) - 0.5)$	800	10	**1.908 (1.823, 1.997)**	**1.124 (1.066, 1.184)**	**1.438 (1.374, 1.506)**	**1.613 (1.505, 1.728)**	**1.774 (1.627, 1.934)**
		20	**2.266 (2.167, 2.370)**	**1.147 (1.085, 1.212)**	**1.390 (1.332, 1.452)**	**1.724 (1.622, 1.831)**	**1.811 (1.681, 1.951)**
	1600	10	**1.484 (1.391, 1.583)**	**1.291 (1.206, 1.381)**	**1.482 (1.389, 1.581)**	**1.587 (1.442, 1.746)**	**1.700 (1.516, 1.906)**
		20	**1.728 (1.622, 1.840)**	**1.325 (1.222, 1.437)**	**1.686 (1.573, 1.808)**	**1.935 (1.754, 2.136)**	**2.129 (1.888, 2.402)**

Table S. 10: Results of **RQ 3** for the experimental setups in Section B. Comparison of mean squared errors for $\hat{\tau}(\boldsymbol{x})$ in the different scenarios. Estimates and simultaneous 95 % confidence intervals were obtained from a normal linear mixed model with log-link. Cells printed in bold font correspond to a superior reference of $Robinson_{\hat{W}}$, cells printed in italics indicate an inferior reference of $Robinson_{\hat{W}}$.

				Mean squared error ratio for RQ 3: Robinson vs. $\text{Robinson}_{\hat{W}}$				
Part	DGP	N	P	Normal	Binomial	Multinomial	Weibull	Cox
A	$\mu(x_3) + 0 \cdot W(x_3)$	800	10	*0.846 (0.770, 0.929)*	0.988 (0.692, 1.410)	*0.779 (0.671, 0.905)*	0.902 (0.749, 1.085)	0.941 (0.735, 1.204)
			20	*0.843 (0.753, 0.944)*	1.090 (0.710, 1.673)	*0.767 (0.628, 0.935)*	0.982 (0.792, 1.218)	1.013 (0.763, 1.345)
		1600	10	*0.783 (0.697, 0.880)*	0.884 (0.555, 1.406)	*0.727 (0.591, 0.896)*	0.808 (0.631, 1.034)	0.817 (0.590, 1.132)
			20	*0.719 (0.618, 0.835)*	0.923 (0.518, 1.644)	*0.699 (0.536, 0.913)*	0.831 (0.614, 1.125)	0.834 (0.559, 1.244)
	$\tau(x_1, x_2)W$	800	10	**1.095 (1.026, 1.168)**	**1.863 (1.732, 2.004)**	1.002 (0.942, 1.065)	**1.274 (1.155, 1.404)**	**1.271 (1.141, 1.416)**
			20	**1.096 (1.024, 1.172)**	**1.238 (1.066, 1.438)**	1.049 (0.987, 1.114)	1.103 (0.998, 1.220)	**1.305 (1.172, 1.453)**
		1600	10	**1.086 (1.007, 1.173)**	**1.217 (1.041, 1.423)**	0.943 (0.869, 1.024)	1.138 (0.983, 1.317)	**1.225 (1.051, 1.428)**
			20	**1.126 (1.032, 1.228)**	1.066 (0.859, 1.323)	1.012 (0.925, 1.106)	1.140 (0.979, 1.328)	**1.211 (1.023, 1.434)**
	$\mu(x_1) + \tau(x_1, x_2)W(x_1)$	800	10	1.055 (0.996, 1.116)	1.000 (0.867, 1.153)	1.042 (0.995, 1.092)	**1.114 (1.022, 1.214)**	1.014 (0.918, 1.121)
			20	0.963 (0.906, 1.023)	1.062 (0.929, 1.214)	**1.225 (1.171, 1.281)**	**1.090 (1.001, 1.187)**	1.063 (0.961, 1.175)
		1600	10	1.052 (0.982, 1.127)	0.994 (0.776, 1.273)	0.975 (0.906, 1.049)	1.098 (0.975, 1.235)	1.029 (0.898, 1.180)
			20	0.999 (0.926, 1.077)	1.043 (0.851, 1.279)	**1.082 (1.009, 1.159)**	0.981 (0.859, 1.119)	0.871 (0.754, 1.007)
	$\mu(x_1) + \tau(x_1, x_2)W$	800	10	**1.066 (1.002, 1.135)**	*0.548 (0.478, 0.628)*	*0.903 (0.861, 0.948)*	1.038 (0.949, 1.135)	1.073 (0.968, 1.189)
			20	1.059 (0.996, 1.125)	1.017 (0.883, 1.171)	*0.912 (0.863, 0.964)*	*0.902 (0.824, 0.988)*	0.950 (0.854, 1.057)
		1600	10	1.022 (0.945, 1.106)	*0.486 (0.407, 0.581)*	*0.836 (0.775, 0.902)*	0.965 (0.850, 1.096)	0.952 (0.830, 1.092)
			20	1.025 (0.941, 1.117)	0.983 (0.819, 1.179)	*0.882 (0.810, 0.960)*	0.945 (0.822, 1.088)	0.897 (0.767, 1.048)
	$\mu(x_3) + \tau(x_1, x_2)W$	800	10	0.982 (0.930, 1.038)	**1.215 (1.072, 1.376)**	*0.796 (0.754, 0.839)*	*0.813 (0.746, 0.885)*	*0.904 (0.819, 0.997)*
			20	*0.872 (0.824, 0.922)*	0.940 (0.824, 1.072)	*0.789 (0.752, 0.828)*	*0.679 (0.624, 0.738)*	*0.697 (0.631, 0.770)*
		1600	10	0.953 (0.885, 1.026)	0.986 (0.839, 1.159)	*0.784 (0.723, 0.849)*	*0.822 (0.725, 0.933)*	*0.853 (0.738, 0.987)*
			20	*0.903 (0.834, 0.976)*	0.879 (0.716, 1.079)	*0.736 (0.681, 0.796)*	*0.676 (0.589, 0.777)*	*0.697 (0.592, 0.821)*
	$\mu(x_3) + \tau(x_1, x_2)W(x_3)$	800	10	1.030 (0.974, 1.090)	**1.260 (1.114, 1.425)**	0.943 (0.888, 1.002)	**1.118 (1.020, 1.226)**	1.066 (0.957, 1.189)
			20	1.021 (0.963, 1.081)	**1.287 (1.125, 1.472)**	1.057 (0.997, 1.120)	**1.140 (1.037, 1.253)**	1.106 (0.989, 1.236)
		1600	10	1.011 (0.942, 1.085)	**1.222 (1.024, 1.458)**	*0.888 (0.818, 0.963)*	1.040 (0.910, 1.189)	1.004 (0.859, 1.174)
			20	1.028 (0.954, 1.109)	1.187 (0.975, 1.445)	0.998 (0.912, 1.092)	0.979 (0.847, 1.132)	0.994 (0.843, 1.171)
	$\tau(x_1, x_2)W(x_3)$	800	10	0.959 (0.909, 1.012)	**1.756 (1.548, 1.991)**	*0.931 (0.879, 0.986)*	0.974 (0.899, 1.055)	0.948 (0.863, 1.041)
			20	*0.906 (0.858, 0.957)*	**1.386 (1.219, 1.576)**	1.019 (0.965, 1.075)	*0.866 (0.799, 0.939)*	*0.896 (0.818, 0.983)*
		1600	10	0.986 (0.923, 1.055)	**1.291 (1.099, 1.516)**	*0.877 (0.810, 0.950)*	0.933 (0.834, 1.044)	0.947 (0.829, 1.081)
			20	0.979 (0.911, 1.053)	1.133 (0.945, 1.359)	*0.869 (0.803, 0.941)*	*0.864 (0.766, 0.973)*	0.973 (0.852, 1.110)
	$\mu(x_3) + \tau(x_1, x_2)W(x_4)$	800	10	*0.671 (0.640, 0.704)*	**1.229 (1.095, 1.380)**	*0.772 (0.735, 0.811)*	*0.677 (0.630, 0.728)*	*0.626 (0.575, 0.682)*
			20	*0.586 (0.558, 0.615)*	0.981 (0.865, 1.113)	*0.786 (0.753, 0.821)*	*0.588 (0.546, 0.633)*	*0.552 (0.507, 0.602)*
		1600	10	*0.789 (0.742, 0.838)*	0.975 (0.830, 1.146)	*0.724 (0.677, 0.774)*	*0.623 (0.564, 0.689)*	*0.561 (0.499, 0.631)*
			20	*0.682 (0.639, 0.727)*	0.867 (0.726, 1.034)	*0.701 (0.656, 0.748)*	*0.463 (0.413, 0.519)*	*0.431 (0.377, 0.492)*
B	$\mu(x_3) + 0 \cdot (W(x_3) - 0.5)$	800	10	0.909 (0.823, 1.005)	1.000 (0.897, 1.115)	*0.826 (0.728, 0.937)*	0.993 (0.840, 1.173)	1.015 (0.811, 1.270)
			20	*0.838 (0.743, 0.944)*	1.046 (0.917, 1.192)	*0.740 (0.627, 0.872)*	0.984 (0.800, 1.209)	1.013 (0.766, 1.341)
		1600	10	*0.764 (0.677, 0.862)*	*0.861 (0.744, 0.997)*	*0.729 (0.620, 0.857)*	0.812 (0.652, 1.012)	0.832 (0.618, 1.120)
			20	*0.717 (0.607, 0.846)*	0.894 (0.744, 1.073)	*0.655 (0.513, 0.838)*	0.803 (0.612, 1.054)	0.815 (0.566, 1.174)
	$\tau(x_1, x_2)(W - 0.5)$	800	10	1.006 (0.942, 1.075)	**1.186 (1.118, 1.259)**	1.001 (0.948, 1.057)	**1.265 (1.160, 1.379)**	**1.467 (1.331, 1.617)**
			20	1.026 (0.961, 1.096)	**1.149 (1.074, 1.228)**	0.983 (0.925, 1.046)	**1.340 (1.213, 1.482)**	**1.497 (1.338, 1.675)**
		1600	10	1.037 (0.954, 1.126)	1.032 (0.951, 1.120)	1.006 (0.934, 1.082)	1.119 (0.989, 1.266)	**1.210 (1.054, 1.389)**
			20	1.027 (0.942, 1.120)	1.035 (0.944, 1.136)	1.003 (0.917, 1.096)	1.151 (0.996, 1.331)	**1.229 (1.039, 1.454)**
	$\mu(x_1) + \tau(x_1, x_2)(W(x_1) - 0.5)$	800	10	0.948 (0.896, 1.004)	1.002 (0.950, 1.057)	1.050 (0.998, 1.104)	**1.173 (1.086, 1.267)**	1.056 (0.965, 1.154)
			20	0.967 (0.912, 1.025)	**1.135 (1.067, 1.207)**	**1.139 (1.082, 1.199)**	0.981 (0.907, 1.061)	0.916 (0.836, 1.004)
		1600	10	0.978 (0.909, 1.053)	*0.906 (0.846, 0.970)*	0.972 (0.911, 1.038)	1.072 (0.960, 1.197)	1.067 (0.937, 1.216)
			20	0.942 (0.872, 1.018)	*0.898 (0.826, 0.976)*	0.980 (0.907, 1.060)	1.042 (0.917, 1.183)	0.940 (0.819, 1.079)
	$\mu(x_1) + \tau(x_1, x_2)(W - 0.5)$	800	10	1.015 (0.954, 1.079)	1.050 (0.991, 1.113)	*0.887 (0.839, 0.937)*	**1.178 (1.084, 1.280)**	**1.187 (1.084, 1.299)**
			20	*0.938 (0.882, 0.997)*	**1.143 (1.069, 1.222)**	0.946 (0.893, 1.001)	**1.168 (1.070, 1.275)**	**1.233 (1.118, 1.360)**
		1600	10	0.996 (0.920, 1.078)	*0.923 (0.855, 0.995)*	*0.927 (0.861, 0.999)*	1.070 (0.952, 1.203)	1.106 (0.967, 1.265)
			20	0.964 (0.884, 1.050)	0.916 (0.833, 1.007)	*0.874 (0.802, 0.951)*	1.007 (0.876, 1.156)	1.037 (0.890, 1.209)
	$\mu(x_3) + \tau(x_1, x_2)(W - 0.5)$	800	10	*0.809 (0.768, 0.851)*	0.997 (0.941, 1.055)	*0.794 (0.754, 0.835)*	*0.851 (0.787, 0.921)*	*0.869 (0.790, 0.955)*
			20	*0.704 (0.668, 0.742)*	*0.857 (0.804, 0.913)*	*0.719 (0.683, 0.757)*	*0.707 (0.651, 0.768)*	*0.745 (0.676, 0.822)*
		1600	10	*0.839 (0.780, 0.903)*	*0.890 (0.821, 0.965)*	*0.800 (0.743, 0.861)*	*0.856 (0.759, 0.965)*	0.883 (0.767, 1.017)
			20	*0.793 (0.737, 0.854)*	*0.823 (0.753, 0.899)*	*0.718 (0.665, 0.775)*	*0.758 (0.663, 0.865)*	*0.758 (0.648, 0.887)*
	$\mu(x_3) + \tau(x_1, x_2)(W(x_3) - 0.5)$	800	10	0.973 (0.920, 1.028)	1.034 (0.977, 1.095)	0.997 (0.948, 1.048)	**1.218 (1.120, 1.325)**	**1.167 (1.051, 1.296)**
			20	*0.937 (0.884, 0.993)*	1.018 (0.955, 1.085)	1.001 (0.948, 1.056)	**1.183 (1.086, 1.288)**	**1.164 (1.050, 1.291)**
		1600	10	0.974 (0.905, 1.047)	*0.885 (0.823, 0.952)*	0.958 (0.894, 1.027)	1.109 (0.994, 1.236)	1.028 (0.899, 1.176)
			20	0.931 (0.864, 1.003)	0.931 (0.850, 1.020)	0.977 (0.900, 1.060)	1.006 (0.885, 1.144)	0.964 (0.823, 1.128)
	$\tau(x_1, x_2)(W(x_3) - 0.5)$	800	10	*0.928 (0.880, 0.979)*	0.993 (0.945, 1.045)	*0.897 (0.852, 0.944)*	**1.095 (1.018, 1.177)**	1.081 (0.989, 1.182)
			20	*0.831 (0.786, 0.877)*	0.999 (0.946, 1.056)	*0.919 (0.876, 0.964)*	1.014 (0.938, 1.095)	0.994 (0.908, 1.088)
		1600	10	*0.868 (0.809, 0.931)*	*0.900 (0.842, 0.963)*	*0.877 (0.822, 0.936)*	1.018 (0.918, 1.128)	0.977 (0.862, 1.108)
			20	*0.839 (0.778, 0.904)*	*0.896 (0.828, 0.970)*	*0.839 (0.778, 0.905)*	0.958 (0.852, 1.077)	0.941 (0.822, 1.077)
	$\mu(x_3) + \tau(x_1, x_2)(W(x_4) - 0.5)$	800	10	*0.524 (0.501, 0.548)*	*0.890 (0.845, 0.938)*	*0.695 (0.664, 0.728)*	*0.620 (0.579, 0.664)*	*0.564 (0.517, 0.615)*
			20	*0.441 (0.422, 0.461)*	*0.872 (0.825, 0.921)*	*0.719 (0.689, 0.751)*	*0.580 (0.546, 0.616)*	*0.552 (0.513, 0.595)*
		1600	10	*0.674 (0.632, 0.719)*	*0.775 (0.724, 0.829)*	*0.675 (0.632, 0.720)*	*0.630 (0.573, 0.694)*	*0.588 (0.525, 0.659)*
			20	*0.579 (0.543, 0.617)*	*0.754 (0.696, 0.818)*	*0.593 (0.553, 0.636)*	*0.517 (0.468, 0.570)*	*0.470 (0.416, 0.530)*

Overlay of prognostic and predictive effects (Part B compared to Part A) did slightly worsen the performance of all methods in smaller samples (except in the absence of a predictive effect, see first column of both figures).

We also inspected if the performance of model-based forests degrades for the Weibull data when the forests do not take the true underlying model as their base model. We compared the performance of model-based forests when using a Cox model compared to a Weibull model (Last row of Figures S. 4 & S. 5). Although knowledge of the true functional form does not enter the Cox modeling process, it did not lead to a major decrease in performance.

C. Dependence plots

Dependence plots depict the treatment effect τ on the prepartum variables - scatter plots for continuous covariates and boxplots for categorical covariates. For categorical covariates, diamonds display the mean effect per group, and for continuous covariates, we provide the smooth conditional mean effect function calculated by a generalized additive model (GAM) with a single smooth term - the covariate under consideration. This evaluation scheme closely follows Dandl, Hothorn, Seibold, Sverdrup, Wager, and Zeileis (2022).

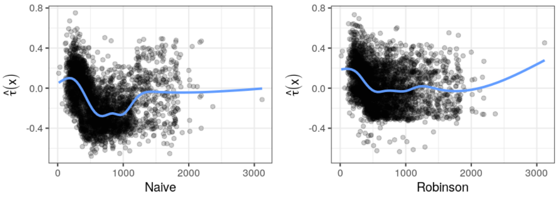

(a) time onset until treatment

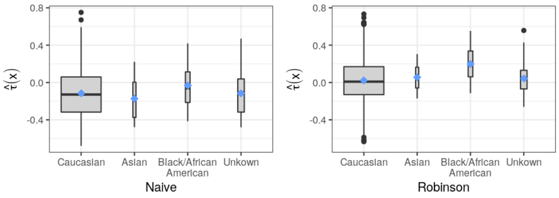

(b) race

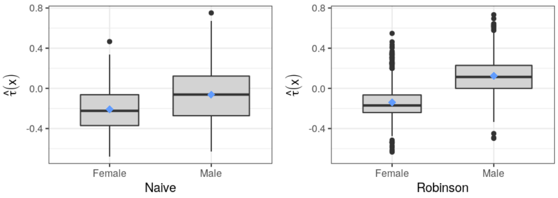

(c) sex

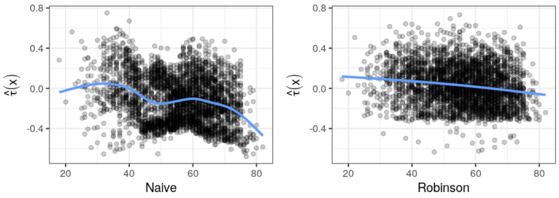

(d) age

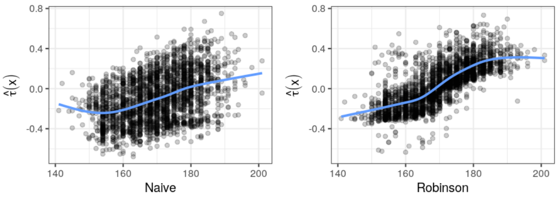

(e) height

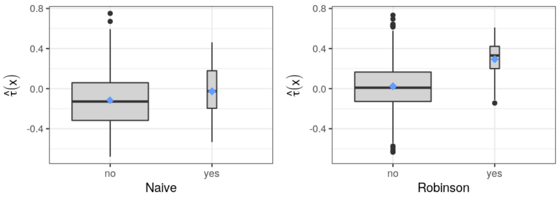

(f) atrophy

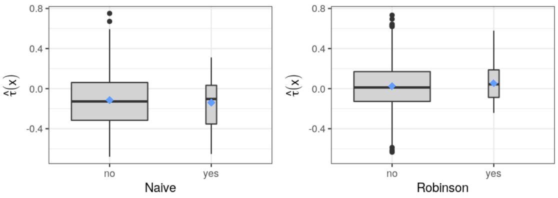

(g) cramps

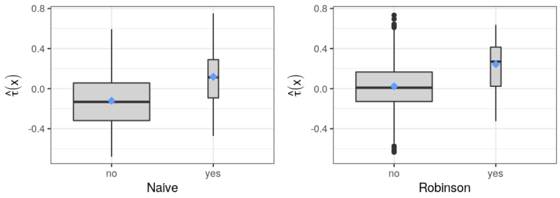

(h) fasciculations

Figure S. 6: Survival time: dependency plot of individual average treatment effects calculated by model-based forest without orthogonalization (left), with Robinson orthogonalization (right). Blue lines and diamond points depict (smooth conditional) mean effects.

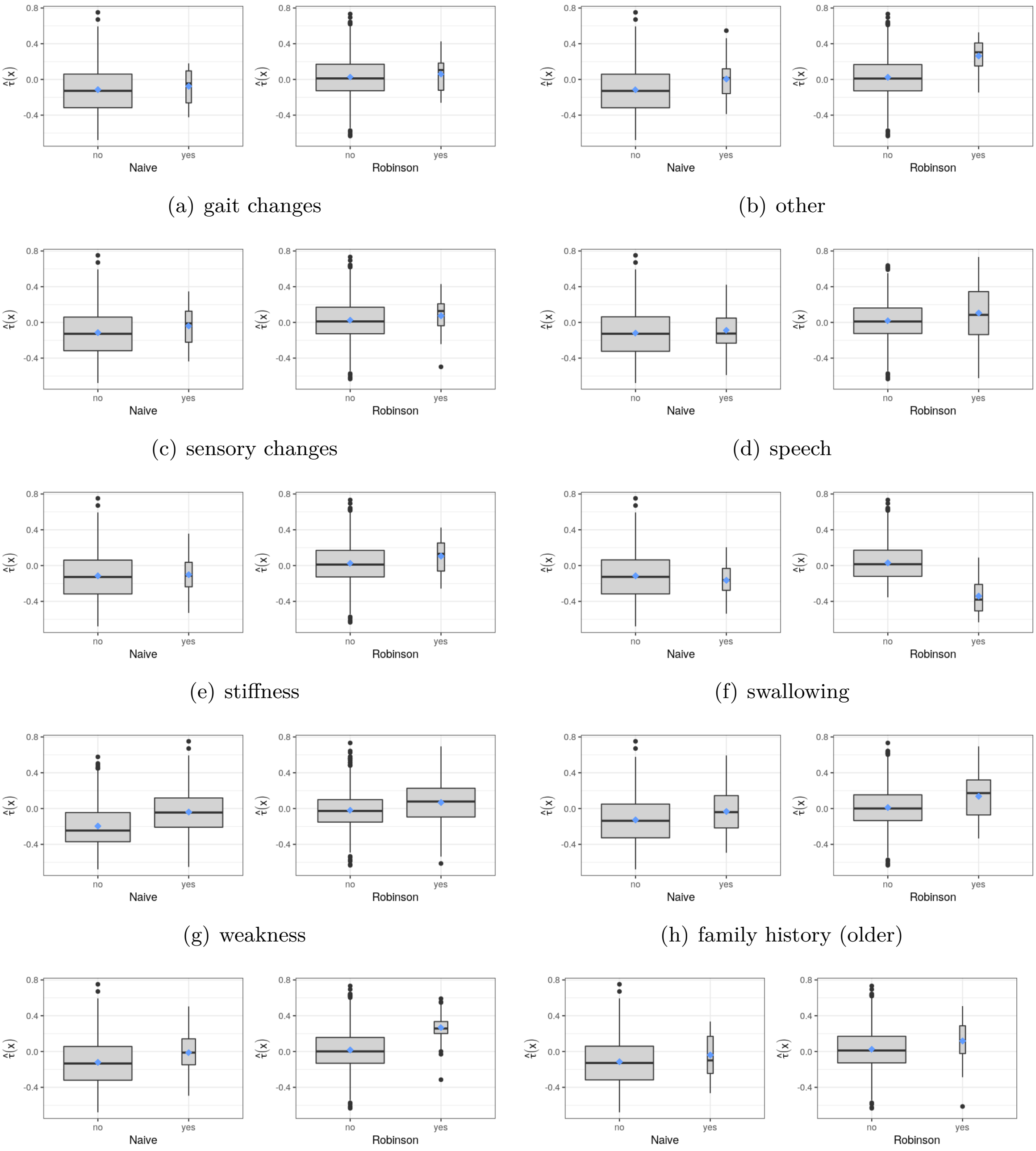

(a) gait changes
(b) other
(c) sensory changes
(d) speech
(e) stiffness
(f) swallowing
(g) weakness
(h) family history (older)
(i) family history (same)
(j) family history (younger)

Figure S. 7: Survival time: dependency plot of individual average treatment effects calculated by model-based forest without orthogonalization (left), with Robinson orthogonalization (right). Blue lines and diamond points depict (smooth conditional) mean effects.

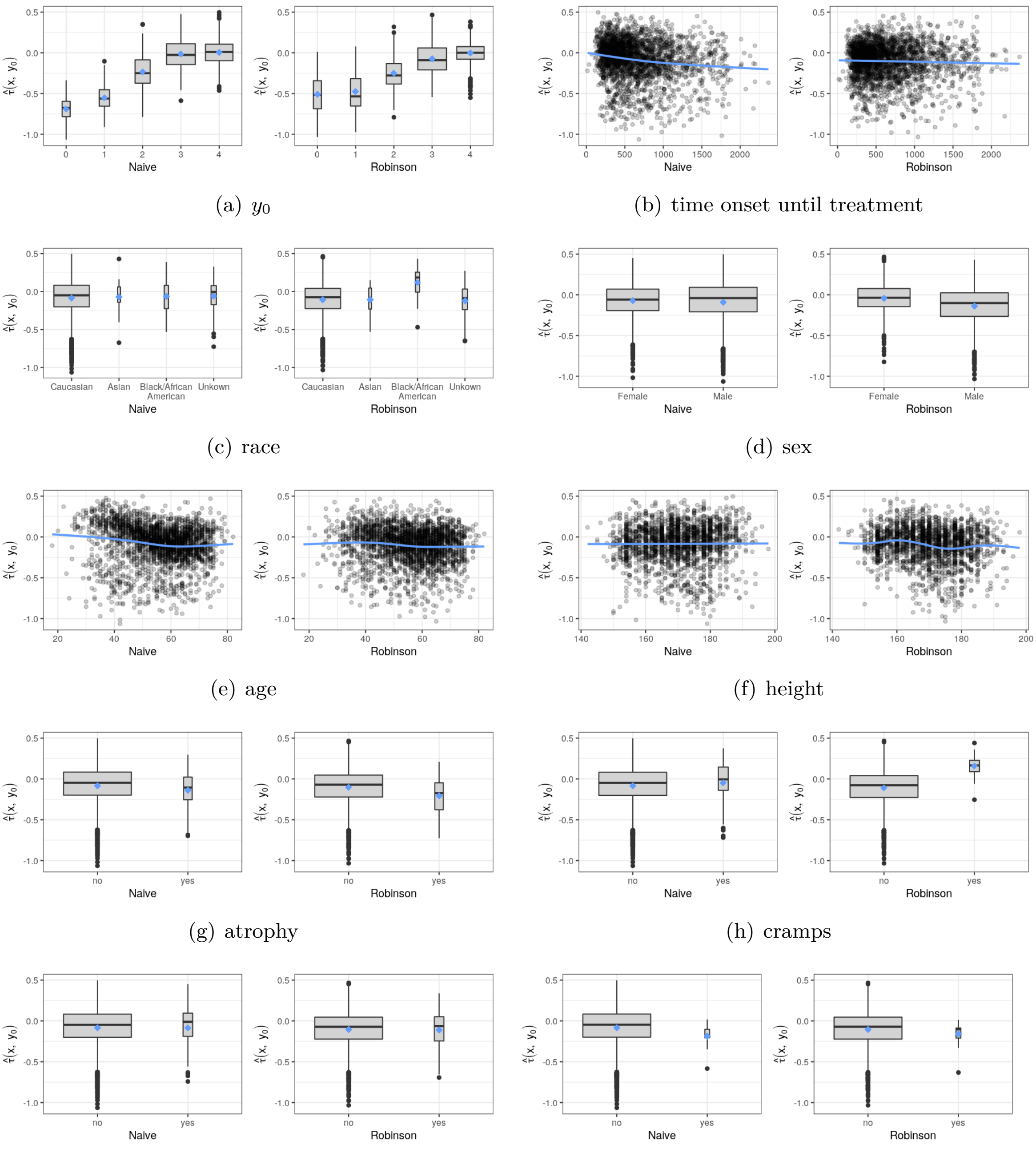

(a) y_0 (b) time onset until treatment

(c) race (d) sex

(e) age (f) height

(g) atrophy (h) cramps

(i) fasciculations (j) gait changes

Figure S. 8: Handwriting ability score: dependency plot of individual average treatment effects calculated by model-based forest without (left) and with Robinson centering (right). Blue lines and diamond points depict (smooth conditional) mean effects.

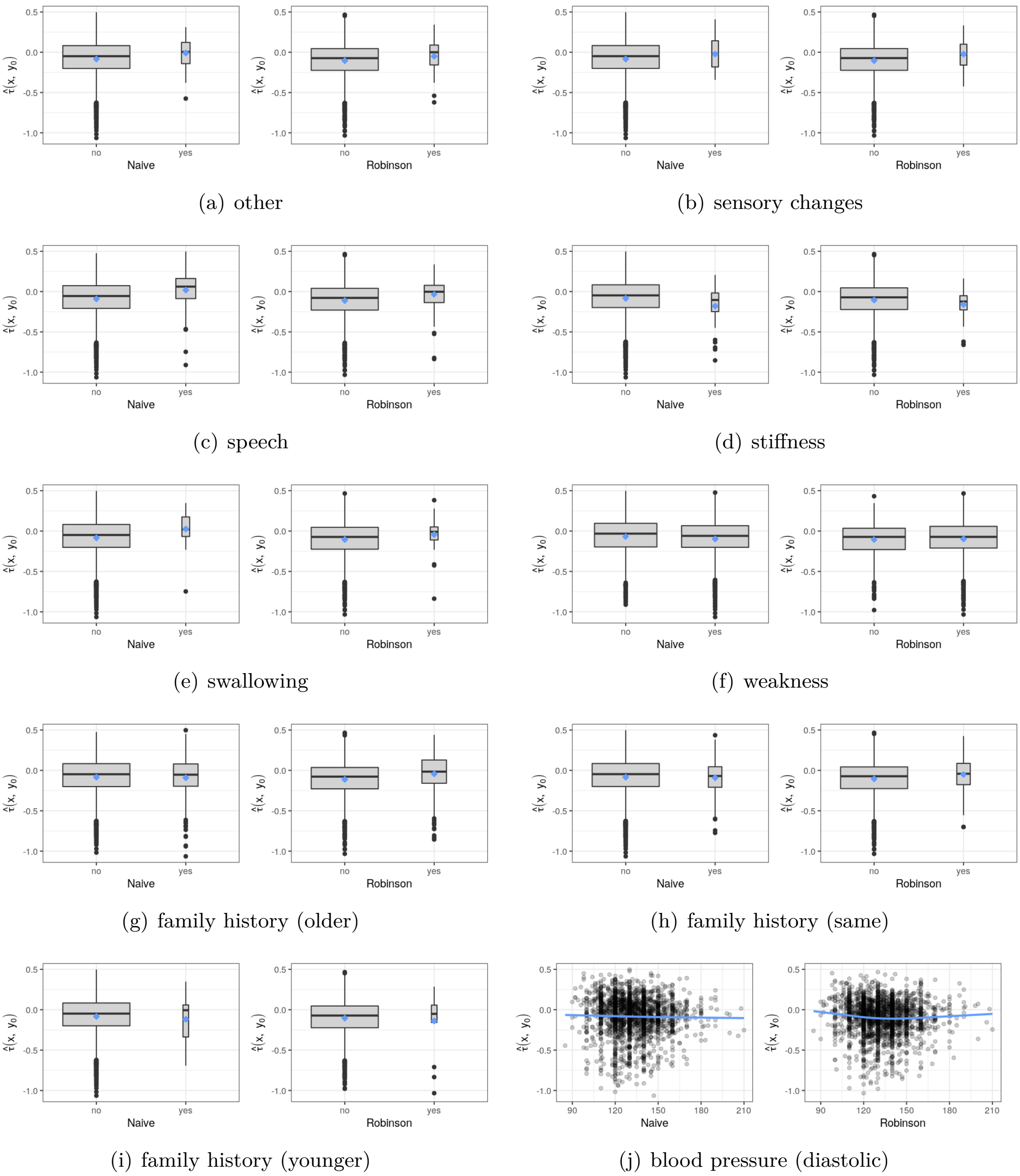

(a) other (b) sensory changes (c) speech (d) stiffness (e) swallowing (f) weakness (g) family history (older) (h) family history (same) (i) family history (younger) (j) blood pressure (diastolic)

Figure S. 9: Handwriting ability score: dependency plot of individual average treatment effects calculated by model-based forest without (left) and with Robinson centering (right). Blue lines and diamond points depict (smooth conditional) mean effects.

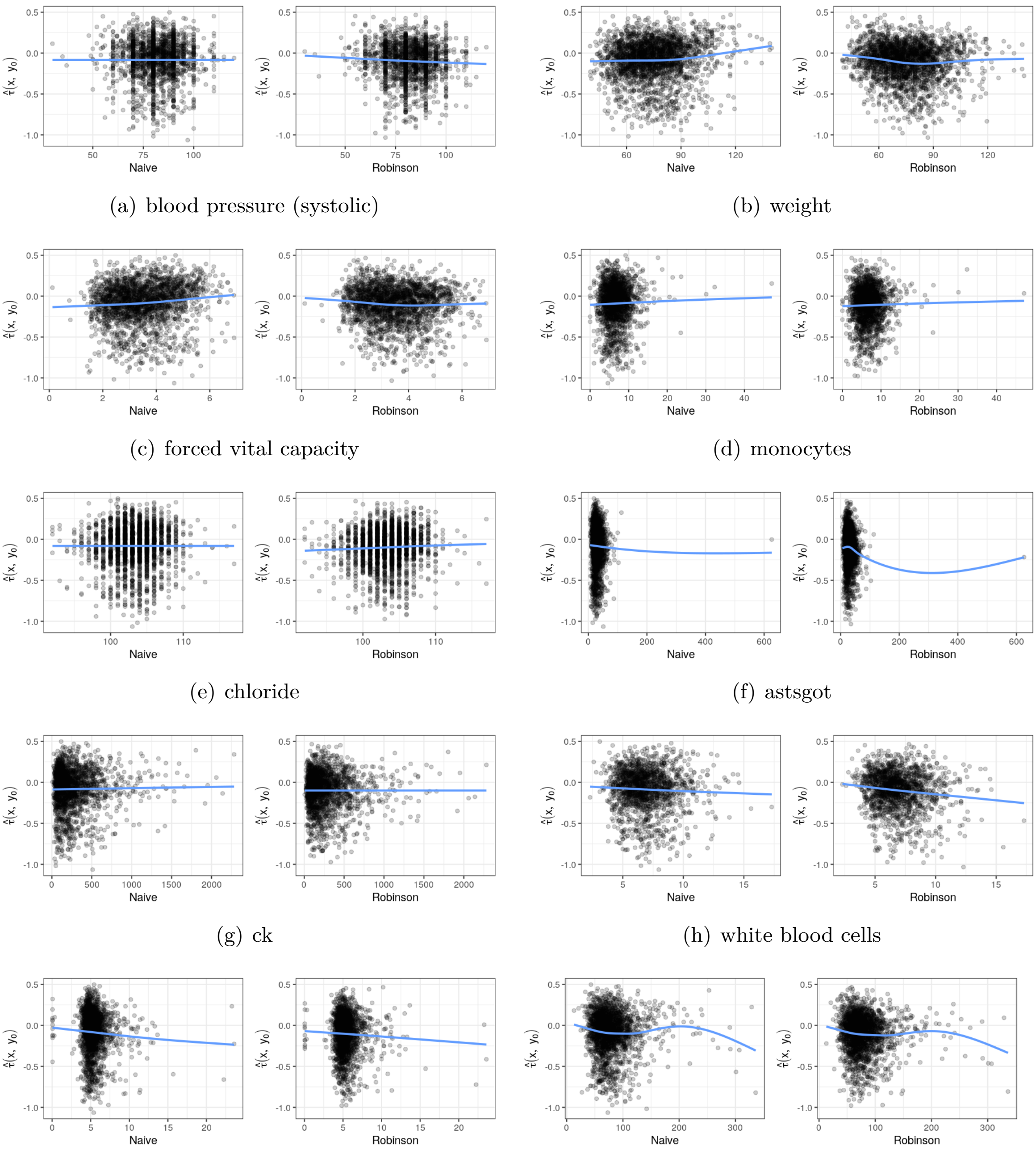

(a) blood pressure (systolic)

(b) weight

(c) forced vital capacity

(d) monocytes

(e) chloride

(f) astsgot

(g) ck

(h) white blood cells

(i) glucose

(j) alkaline phosphatase

Figure S. 10: Handwriting ability score: dependency plot of individual average treatment effects calculated by model-based forest without (left) and with Robinson centering (right). Blue lines and diamond points depict (smooth conditional) mean effects.

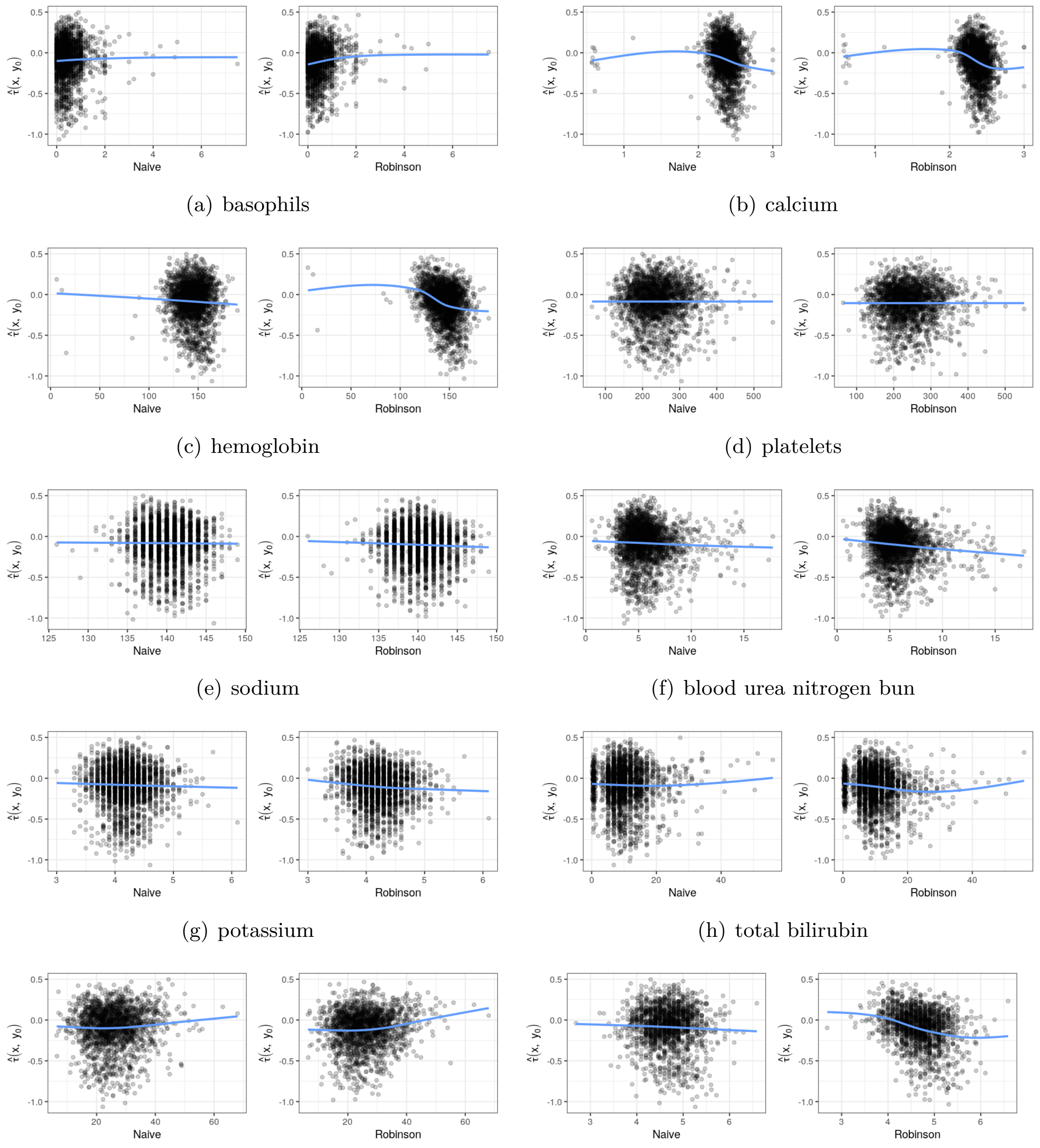

(a) basophils (b) calcium

(c) hemoglobin (d) platelets

(e) sodium (f) blood urea nitrogen bun

(g) potassium (h) total bilirubin

(i) lymphocytes (j) red blood cells

Figure S. 11: Handwriting ability score: dependency plot of individual average treatment effects calculated by model-based forest without (left) and with Robinson centering (right). Blue lines and diamond points depict (smooth conditional) mean effects.

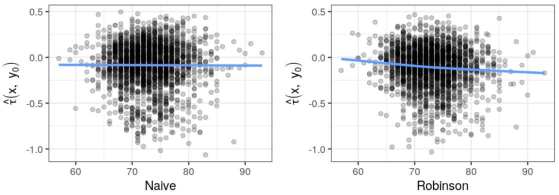

(a) protein

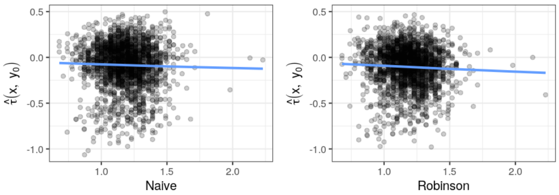

(b) phosphorus

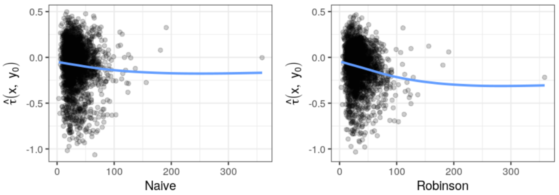

(c) altsgpt

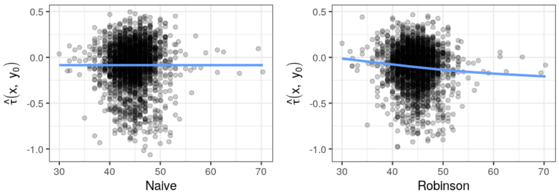

(d) albumin

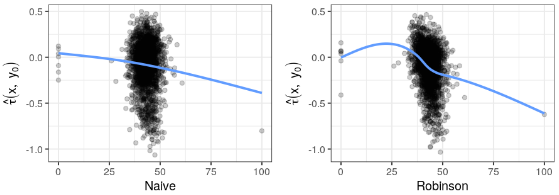

(e) hematocrit

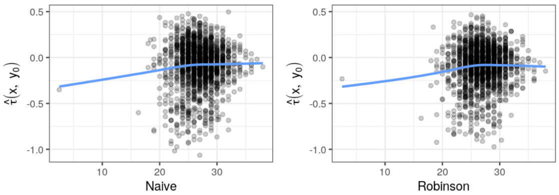

(f) bicarbonate

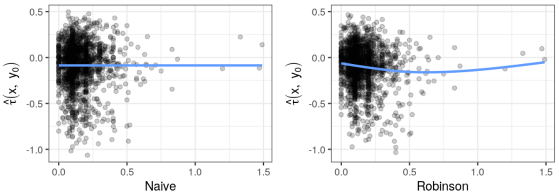

(g) absolute eosinophils count

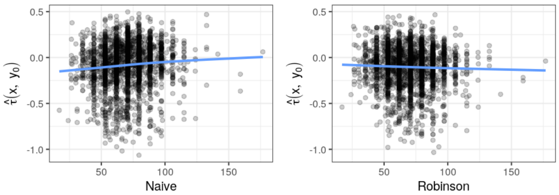

(h) creatinine

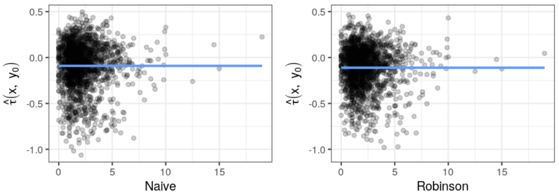

(i) eosinophils

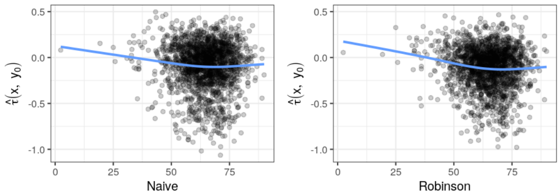

(j) neutrophils

Figure S. 12: Handwriting ability score: dependency plot of individual average treatment effects calculated by model-based forest without (left) and with Robinson centering (right). Blue lines and diamond points depict (smooth conditional) mean effects.

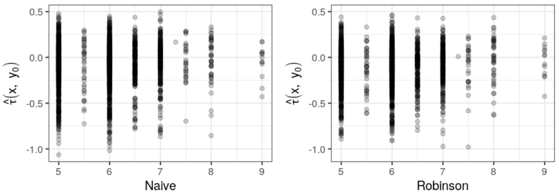

(a) urine ph

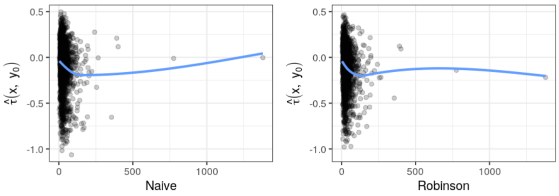

(b) glutamyltransferase

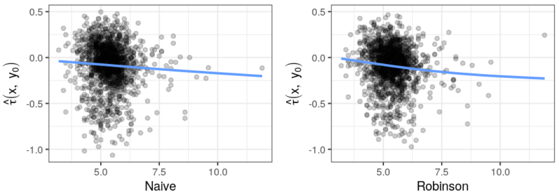

(c) glycated hemoglobin

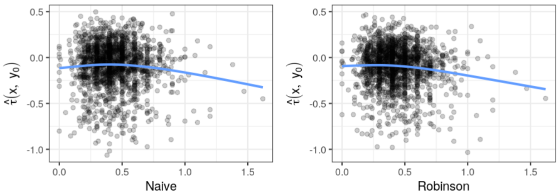

(d) absolute monocyte count

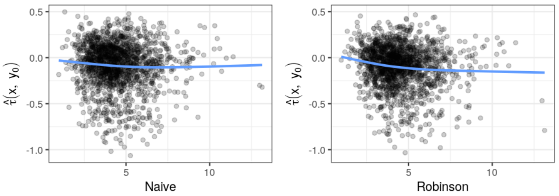

(e) absolute neutrophil count

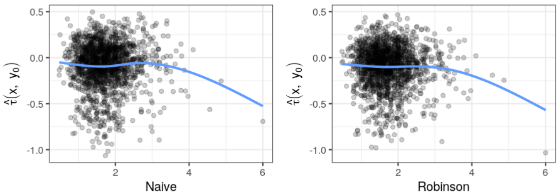

(f) absolute lymphocyte count

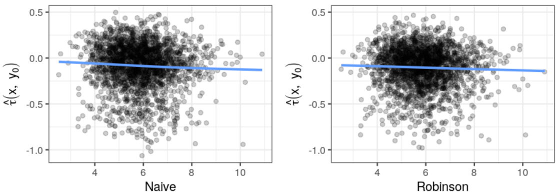

(g) total cholesterol

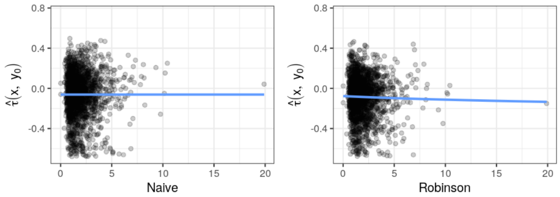

(h) triglycerides

Figure S. 13: Handwriting ability score: dependency plot of individual average treatment effects calculated by model-based forest without (left) and with Robinson centering (right). Blue lines and diamond points depict (smooth conditional) mean effects.

References

Aalen OO, Cook RJ, Røysland K (2015). "Does Cox Analysis of a Randomized Survival Study Yield a Causal Treatment Effect?" *Lifetime Data Analysis*, **21**(4), 579–593. doi:10.1007/s10985-015-9335-y.

Athey S, Tibshirani J, Wager S (2019). "Generalized Random Forests." *The Annals of Statistics*, **47**(2), 1148–1178. doi:10.1214/18-aos1709.

Dandl S, Hothorn T, Seibold H, Sverdrup E, Wager S, Zeileis A (2022). "What Makes Forest-Based Heterogeneous Treatment Effect Estimators Work?" *Technical report*, arXiv 2206.10323. URL https://arxiv.org/abs/2206.10323.

Daniel R, Zhang J, Farewell D (2021). "Making Apples from Oranges: Comparing Non-collapsible Effect Estimators and Their Standard Errors after Adjustment for Different Covariate Sets." *Biometrical Journal*, **63**(3), 528–557. doi:https://doi.org/10.1002/bimj.201900297.

Gao Z, Hastie T (2022). "Estimating Heterogeneous Treatment Effects for General Responses." *Technical report*, arXiv 2103.04277 v4. URL https://arxiv.org/abs/2103.04277.

Greenland S (1996). "Absence of Confounding Does Not Correspond to Collapsibility of the Rate Ratio or Rate Difference." *Epidemiology*, **7**, 498–501.

Greenland S, Pearl J, Robins JM (1999). "Confounding and Collapsibility in Causal Inference." *Statistical Science*, **14**(1), 29–46. doi:10.1214/ss/1009211805.

Robinson PM (1988). "Root-N-Consistent Semiparametric Regression." *Econometrica*, **56**(4), 931–954. doi:10.2307/1912705.

Wager S, Athey S (2018). "Estimation and Inference of Heterogeneous Treatment Effects Using Random Forests." *Journal of the American Statistical Association*, **113**(523), 1228–1242. doi:10.1080/01621459.2017.1319839.

Affiliation:

Susanne Dandl, Andreas Bender
Institut für Statistik, Ludwig-Maximilians-Universität München, Germany
Munich Center for Machine Learning (MCML), Germany

Torsten Hothorn
Institut für Epidemiologie, Biostatistik und Prävention, Universität Zürich
Hirschengraben 84, CH-8001 Zürich, Switzerland
E-mail: Torsten.Hothorn@R-project.org
